# Supplementary material for: Synaptic targets of photoreceptors specialized to detect color and skylight polarization in Drosophila
Source: eLife. 2021 Dec 16;10:e71858. doi: 10.7554/eLife.71858 (PMC8789284; doi:10.7554/eLife.71858)
Supplement: Supplementary file 1. [file elife-71858-supp1.pdf]

Supplementary File 1: Tables of all seed column R7, R8, R7-DRA and R8-DRA target cells by type

Contents

R7 and R8 outgoing Dm9 3
R7 and R8 outgoing Dm8 3
R7 and R8 outgoing MeTu 3
R7 and R8 outgoing R7 4
R7 and R8 outgoing Tm5c 4
R7 and R8 outgoing Tm20 4
R7 and R8 outgoing Mi15 4
R7 and R8 outgoing Mi4 4
R7 and R8 outgoing ML1 5
R7 and R8 outgoing Dm2 5
R7 and R8 outgoing Dm11 5
R7 and R8 outgoing L3 5
R7 and R8 outgoing Mi1 5
R7 and R8 outgoing R8 5
R7 and R8 outgoing Tm5a 6
R7 and R8 outgoing Tm5b 6
R7 and R8 outgoing Tm 6
R7 and R8 outgoing Tm5b-like 6
R7 and R8 outgoing Mi9 6
R7 and R8 outgoing L1 7
R7 and R8 outgoing aMe12 7
R7 and R8 outgoing Dm 7
R7 and R8 outgoing ML-VPN1 7
R7 and R8 outgoing C2 7
R7 and R8 outgoing Mt-VPN 7
R7 and R8 outgoing Mti 8
R7 and R8 outgoing Tm5a-like 8
R7 and R8 outgoing TmY10 8
R7 and R8 outgoing Mi10 8

R7 and R8 outgoing Mi 8
R7 and R8 outgoing C3 8
R7 and R8 outgoing Identified-<3 9
R7 and R8 outgoing Unidentified->=3 9
R7 and R8 outgoing Unidentified-<3 10
R7 and R8 incoming Dm9 11
R7 and R8 incoming R8 11
R7 and R8 incoming R7 11
R7 and R8 incoming Mt-VPN 11
R7 and R8 incoming C2 11
R7 and R8 incoming L3 11
R7 and R8 incoming Identified-<3 12
R7 and R8 incoming Unidentified-<3 12
R7-DRA and R8-DRA outgoing Dm-DRA1 12
R7-DRA and R8-DRA outgoing Dm9 13
R7-DRA and R8-DRA outgoing MeTu-DRA 13
R7-DRA and R8-DRA outgoing R7-DRA 13
R7-DRA and R8-DRA outgoing Dm-DRA2 14
R7-DRA and R8-DRA outgoing Dm2 14
R7-DRA and R8-DRA outgoing R8-DRA 14
R7-DRA and R8-DRA outgoing Mi15 14
R7-DRA and R8-DRA outgoing Mti-DRA-1 14
R7-DRA and R8-DRA outgoing MeMe-DRA 15
R7-DRA and R8-DRA outgoing L3 15
R7-DRA and R8-DRA outgoing VPN-DRA 15
R7-DRA and R8-DRA outgoing L1 15
R7-DRA and R8-DRA outgoing Tm20 15
R7-DRA and R8-DRA outgoing Mti-DRA-2 15
R7-DRA and R8-DRA outgoing Mi1 16
R7-DRA and R8-DRA outgoing MeTu 16
R7-DRA and R8-DRA outgoing Tm5-like 16
R7-DRA and R8-DRA outgoing Mi9 16
R7-DRA and R8-DRA outgoing Dm11 16
R7-DRA and R8-DRA outgoing aMe12 16
R7-DRA and R8-DRA outgoing TmY 16
R7-DRA and R8-DRA outgoing ML-VPN2 17
R7-DRA and R8-DRA outgoing C2 17
R7-DRA and R8-DRA outgoing Identified-<3 17

R7-DRA and R8-DRA outgoing Unidentified->=3 17
R7-DRA and R8-DRA outgoing Unidentified-<3 18
R7-DRA and R8-DRA incoming Dm9 19
R7-DRA and R8-DRA incoming R8-DRA 19
R7-DRA and R8-DRA incoming R7-DRA 19
R7-DRA and R8-DRA incoming C2 19
R7-DRA and R8-DRA incoming Mi15 19
R7-DRA and R8-DRA incoming Identified-<3 19
R7-DRA and R8-DRA incoming Unidentified-<3 20

R7 and R8 outgoing Dm9

name	skid	pR7a	pR7b	yR7a	yR7b	pR8a	pR8b	yR8a	yR8b	total	%R7	%R8	%p	%y
Putative Dm9 11452428 MF	11452427	29	37	26	23	70	65	52	66	368	31.2	68.8	54.6	45.4
Putative Dm9 11447062 CL	11447061	16	0	11	0	0	0	12	0	39	69.2	30.8	41.0	59.0
Putative Dm9 11450496 CL	11450495	0	0	0	4	0	0	6	5	15	26.7	73.3	0.0	100.0
Putative Dm9 11484680 MF	11484679	8	0	0	0	0	0	0	0	8	100.0	0.0	100.0	0.0
Putative Dm9 11444387 HL	11444386	0	4	0	0	0	4	0	0	8	50.0	50.0	100.0	0.0
Putative Dm9 11454715 CL	11454714	0	0	0	2	0	3	0	2	7	28.6	71.4	42.9	57.1
Total		53	41	37	29	70	72	70	73	445	36.0	64.0	53.0	47.0

R7 and R8 outgoing Dm8

name	skid	pR7a	pR7b	yR7a	yR7b	pR8a	pR8b	yR8a	yR8b	total	%R7	%R8	%p	%y
Putative Dm8 11453022 CL	11453021	4	4	19	35	0	0	0	0	62	100	0	12.9	87.1
Putative Dm8 10411812 MF	10411811	21	40	0	0	0	0	0	0	61	100	0	100.0	0.0
Putative Dm8 10208776 MF	10208775	15	3	35	6	0	0	0	0	59	100	0	30.5	69.5
Putative Dm8 11453312 CL	11453311	9	17	8	15	0	0	0	0	49	100	0	53.1	46.9
Putative Dm8 10109587 MF	10109586	39	4	0	0	0	0	0	0	43	100	0	100.0	0.0
Putative Dm8 11500072 HL	11500071	1	17	0	0	0	0	0	0	18	100	0	100.0	0.0
Putative Dm8 11448877 HL	11523807	0	17	0	0	0	0	0	0	17	100	0	100.0	0.0
Putative Dm8 10419715 MF	10419714	14	0	0	2	0	0	0	0	16	100	0	87.5	12.5
Putative Dm8 11447521 MF	11447520	0	0	9	5	0	0	0	0	14	100	0	0.0	100.0
Putative Dm8 10410433 MF	10410432	10	3	0	0	0	0	0	0	13	100	0	100.0	0.0
Putative Dm8 10196190 MF	10196189	10	0	3	0	0	0	0	0	13	100	0	76.9	23.1
Putative Dm8 10245572 MF	10245571	0	0	2	9	0	0	0	0	11	100	0	0.0	100.0
Putative Dm8 10971699 MF	10971698	0	0	0	10	0	0	0	0	10	100	0	0.0	100.0
Putative Dm8 11445682 MF	11445681	7	0	0	0	0	0	0	0	7	100	0	100.0	0.0
Putative Dm8 10995249 MF	10995248	0	0	0	3	0	0	0	0	3	100	0	0.0	100.0
Total		130	105	76	85	0	0	0	0	396	100	0	59.3	40.7

R7 and R8 outgoing MeTu

name	skid	pR7a	pR7b	yR7a	yR7b	pR8a	pR8b	yR8a	yR8b	total	%R7	%R8	%p	%y
Putative MeTu 10409864 MF	10409863	22	13	17	12	0	0	0	0	64	100	0	54.7	45.3
Putative MeTu 11455123 CL	11455122	0	21	4	14	0	0	0	0	39	100	0	53.8	46.2
Putative MeTu 11455157 CL	11455156	6	9	9	12	0	0	0	0	36	100	0	41.7	58.3
Putative MeTu 10409693 MF	10409692	11	0	6	0	0	0	0	0	17	100	0	64.7	35.3
Putative MeTu 11448396 MF	11448395	0	0	8	4	0	0	0	0	12	100	0	0.0	100.0
Putative MeTu 11455113 CL	11455112	0	0	0	4	0	0	0	0	4	100	0	0.0	100.0
Putative MeTu 11499694 CL	11499693	0	0	3	0	0	0	0	0	3	100	0	0.0	100.0
Total		39	43	47	46	0	0	0	0	175	100	0	46.9	53.1

R7 and R8 outgoing R7

name	skid	pR7a	pR7b	yR7a	yR7b	pR8a	pR8b	yR8a	yR8b	total	%R7	%R8	%p	%y
Putative R7 col G1 10585941 MF	10585940	0	0	0	0	0	0	46	0	46	0.0	100.0	0.0	100.0
Putative R7 col A1 10082583 MF	10082582	0	0	0	0	39	0	0	0	39	0.0	100.0	100.0	0.0
Putative R7 col I1 10538511 MF	10538510	0	0	0	0	0	36	0	0	36	0.0	100.0	100.0	0.0
Putative R7 col H1 10653594 MF	10653593	0	0	0	0	0	0	0	36	36	0.0	100.0	0.0	100.0
Putative R7 10653781 MF	10653780	0	0	0	3	0	0	0	0	3	100.0	0.0	0.0	100.0
Total		0	0	0	3	39	36	46	36	160	1.9	98.1	46.9	53.1

R7 and R8 outgoing Tm5c

name	skid	pR7a	pR7b	yR7a	yR7b	pR8a	pR8b	yR8a	yR8b	total	%R7	%R8	%p	%y
Putative Tm5c 11450506 CL	11450505	0	0	0	2	0	0	21	22	45	4.4	95.6	0.0	100.0
Putative Tm5c 11470103 MF	11470102	0	0	1	0	0	0	23	0	24	4.2	95.8	0.0	100.0
Putative Tm5c 11449561 MF	11449560	2	5	0	0	6	4	3	4	24	29.2	70.8	70.8	29.2
Putative Tm5c 11574444 MF	11574443	0	0	2	0	0	0	16	0	18	11.1	88.9	0.0	100.0
Putative Tm5c 11473479 CL	11473478	0	0	0	4	0	0	0	14	18	22.2	77.8	0.0	100.0
Putative Tm5c 11485095 MF	11485094	1	0	0	0	10	0	0	0	11	9.1	90.9	100.0	0.0
Total		3	5	3	6	16	4	63	40	140	12.1	87.9	20.0	80.0

R7 and R8 outgoing Tm20

name	skid	pR7a	pR7b	yR7a	yR7b	pR8a	pR8b	yR8a	yR8b	total	%R7	%R8	%p	%y
Putative Tm20 col I1 11444393 HL	11444392	0	2	0	0	0	35	0	0	37	5.4	94.6	100.0	0.0
Putative Tm20 col H1 11450553 CL	11450552	0	0	0	2	0	0	0	35	37	5.4	94.6	0.0	100.0
Putative Tm20 col A1 10423775 MF	10423774	3	0	0	0	32	0	0	0	35	8.6	91.4	100.0	0.0
Putative Tm20 col G1 11473669 MF	11473668	0	0	1	0	0	0	30	0	31	3.2	96.8	0.0	100.0
Total		3	2	1	2	32	35	30	35	140	5.7	94.3	51.4	48.6

R7 and R8 outgoing Mi15

name	skid	pR7a	pR7b	yR7a	yR7b	pR8a	pR8b	yR8a	yR8b	total	%R7	%R8	%p	%y
Putative Mi15 11445262 MF	11445261	5	0	2	0	13	0	22	0	42	16.7	83.3	42.9	57.1
Putative Mi15 11450568 CL	11450567	0	0	0	3	0	0	0	32	35	8.6	91.4	0.0	100.0
Putative Mi15 11453819 CL	11453818	0	0	2	0	0	0	25	2	29	6.9	93.1	0.0	100.0
Putative Mi15 11484381 HL	11484380	0	1	0	0	0	25	0	0	26	3.8	96.2	100.0	0.0
Total		5	1	4	3	13	25	47	34	132	9.8	90.2	33.3	66.7

R7 and R8 outgoing Mi4

name	skid	pR7a	pR7b	yR7a	yR7b	pR8a	pR8b	yR8a	yR8b	total	%R7	%R8	%p	%y
Putative Mi4 col G1 11481479 MF	11481478	0	0	0	0	0	0	30	0	30	0	100	0	100
Putative Mi4 col I1 11467500 HL	11467499	0	0	0	0	0	29	0	0	29	0	100	100	0
Putative Mi4 col A1 11465885 CL	11465884	0	0	0	0	29	0	0	0	29	0	100	100	0
Putative Mi4 col H1 11473654 CL	11473653	0	0	0	0	0	0	0	28	28	0	100	0	100
Total		0	0	0	0	29	29	30	28	116	0	100	50	50

R7 and R8 outgoing ML1

name	skid	pR7a	pR7b	yR7a	yR7b	pR8a	pR8b	yR8a	yR8b	total	%R7	%R8	%p	%y
Putative ML1 11472158 CL	11472157	0	0	0	0	11	4	13	13	41	0	100	36.6	63.4
Putative ML1 11458491 CL	11458490	0	0	0	0	13	12	4	0	29	0	100	86.2	13.8
Putative ML1 11471220 CL	11471219	0	0	0	0	0	13	4	7	24	0	100	54.2	45.8
Putative ML1 11458827 CL	11458826	0	0	0	0	5	0	0	0	5	0	100	100.0	0.0
Total		0	0	0	0	29	29	21	20	99	0	100	58.6	41.4

R7 and R8 outgoing Dm2

name	skid	pR7a	pR7b	yR7a	yR7b	pR8a	pR8b	yR8a	yR8b	total	%R7	%R8	%p	%y
Putative Dm2 11448823 HL	11448822	0	12	0	0	0	24	0	0	36	33.3	66.7	100.0	0.0
Putative Dm2 10499250 MF	10499249	6	0	0	0	29	0	0	0	35	17.1	82.9	100.0	0.0
Putative Dm2 11453278 CL	11453277	0	0	0	3	0	0	0	15	18	16.7	83.3	0.0	100.0
Putative Dm2 11466217 CL	11466216	0	0	0	0	7	0	0	0	7	0.0	100.0	100.0	0.0
Total		6	12	0	3	36	24	0	15	96	21.9	78.1	81.2	18.8

R7 and R8 outgoing Dm11

name	skid	pR7a	pR7b	yR7a	yR7b	pR8a	pR8b	yR8a	yR8b	total	%R7	%R8	%p	%y
Putative Dm11 11450454 CL	11450453	15	4	9	18	2	0	3	1	52	88.5	11.5	40.4	59.6
Putative Dm11 11444399 HL	11444398	0	9	0	0	0	2	0	0	11	81.8	18.2	100.0	0.0
Total		15	13	9	18	2	2	3	1	63	87.3	12.7	50.8	49.2

R7 and R8 outgoing L3

name	skid	pR7a	pR7b	yR7a	yR7b	pR8a	pR8b	yR8a	yR8b	total	%R7	%R8	%p	%y
Putative L3 col H1 11450470 CL	11450469	0	0	0	12	0	0	0	7	19	63.2	36.8	0.0	100.0
Putative L3 col A1 11445252 MF	11445251	10	0	0	0	6	0	0	0	16	62.5	37.5	100.0	0.0
Putative L3 col I1 11448597 HL	11448596	0	9	0	0	0	6	0	0	15	60.0	40.0	100.0	0.0
Putative L3 col G1 11453914 CL	11453913	0	0	5	0	0	0	6	0	11	45.5	54.5	0.0	100.0
Total		10	9	5	12	6	6	6	7	61	59.0	41.0	50.8	49.2

R7 and R8 outgoing Mi1

name	skid	pR7a	pR7b	yR7a	yR7b	pR8a	pR8b	yR8a	yR8b	total	%R7	%R8	%p	%y
Putative Mi1 col I1 11471702 HL	11471701	0	0	0	0	0	17	0	0	17	0	100	100.0	0.0
Putative Mi1 col H1 11472781 CL	11472780	0	0	0	0	0	0	0	14	14	0	100	0.0	100.0
Putative Mi1 col G1 11470015 MF	11470014	0	0	0	0	0	0	14	0	14	0	100	0.0	100.0
Putative Mi1 col A1 11458128 CL	11458127	0	0	0	0	14	0	0	0	14	0	100	100.0	0.0
Total		0	0	0	0	14	17	14	14	59	0	100	52.5	47.5

R7 and R8 outgoing R8

name	skid	pR7a	pR7b	yR7a	yR7b	pR8a	pR8b	yR8a	yR8b	total	%R7	%R8	%p	%y
Putative R8 col A1 10086692 MF	10086691	20	0	0	0	1	0	0	0	21	95.2	4.8	100.0	0.0
Putative R8 col H1 11468319 CL	11468318	0	0	0	19	0	0	0	0	19	100.0	0.0	0.0	100.0
Putative R8 col I1 11466409 HL	11466408	0	11	0	0	0	0	0	0	11	100.0	0.0	100.0	0.0
Putative R8 col G1 10629255 MF	10629254	0	0	7	0	0	0	0	0	7	100.0	0.0	0.0	100.0
Total		20	11	7	19	1	0	0	0	58	98.3	1.7	55.2	44.8

R7 and R8 outgoing Tm5a

name	skid	pR7a	pR7b	yR7a	yR7b	pR8a	pR8b	yR8a	yR8b	total	%R7	%R8	%p	%y
Putative Tm5a 11447184 MF	11447183	0	0	29	0	0	0	0	0	29	100	0	0	100
Putative Tm5a 11453107 CL	11453106	0	0	0	24	0	0	0	0	24	100	0	0	100
Total		0	0	29	24	0	0	0	0	53	100	0	0	100

R7 and R8 outgoing Tm5b

name	skid	pR7a	pR7b	yR7a	yR7b	pR8a	pR8b	yR8a	yR8b	total	%R7	%R8	%p	%y
Putative Tm5b 11448828 HL	11448827	0	19	0	0	0	1	0	6	26	73.1	26.9	76.9	23.1
Putative Tm5b 10356413 MF	10356412	20	0	0	0	2	0	0	0	22	90.9	9.1	100.0	0.0
Total		20	19	0	0	2	1	0	6	48	81.2	18.8	87.5	12.5

R7 and R8 outgoing Tm

name	skid	pR7a	pR7b	yR7a	yR7b	pR8a	pR8b	yR8a	yR8b	total	%R7	%R8	%p	%y
Putative Tm 11544671 HL	11544670	0	10	0	0	0	0	0	0	10	100.0	0.0	100.0	0.0
Putative Tm 11671250 MF	11671249	0	0	0	0	0	0	0	7	7	0.0	100.0	0.0	100.0
Putative Tm 10692408 HL	10692407	0	0	0	0	0	0	3	2	5	0.0	100.0	0.0	100.0
Putative Tm 11474296 CL	11474295	0	0	0	0	0	0	0	4	4	0.0	100.0	0.0	100.0
Putative Tm 11458217 CL	11458216	0	0	0	0	4	0	0	0	4	0.0	100.0	100.0	0.0
Putative Tm 11455044 MF	11455043	0	0	0	4	0	0	0	0	4	100.0	0.0	0.0	100.0
Putative Tm 11445921 MF	11445920	3	0	0	0	1	0	0	0	4	75.0	25.0	100.0	0.0
Putative Tm 11459161 CL	11459160	1	0	0	0	2	0	0	0	3	33.3	66.7	100.0	0.0
Putative Tm 11450248 HL	11450247	0	3	0	0	0	0	0	0	3	100.0	0.0	100.0	0.0
Total		4	13	0	4	7	0	3	13	44	47.7	52.3	54.5	45.5

R7 and R8 outgoing Tm5b-like

name	skid	pR7a	pR7b	yR7a	yR7b	pR8a	pR8b	yR8a	yR8b	total	%R7	%R8	%p	%y
Putative Tm5b-like 11447921 MF	11447920	0	0	7	0	0	0	16	0	23	30.4	69.6	0	100
Putative Tm5b-like 11447511 CL	11447510	0	0	4	0	0	0	7	0	11	36.4	63.6	0	100
Putative Tm5b-like 11468647 CL	11468646	0	0	2	1	0	0	0	4	7	42.9	57.1	0	100
Total		0	0	13	1	0	0	23	4	41	34.1	65.9	0	100

R7 and R8 outgoing Mi9

name	skid	pR7a	pR7b	yR7a	yR7b	pR8a	pR8b	yR8a	yR8b	total	%R7	%R8	%p	%y
Putative Mi9 col A1 10422285 MF	10422284	3	0	0	0	12	0	0	0	15	20.0	80.0	100.0	0.0
Putative Mi9 col I1 11467629 HL	11467628	0	0	0	0	0	14	0	0	14	0.0	100.0	100.0	0.0
Putative Mi9 col G1 11447431 CL	11447430	0	0	5	0	0	0	1	0	6	83.3	16.7	0.0	100.0
Putative Mi9 col H1 11453112 CL	11453111	0	0	0	4	0	0	0	0	4	100.0	0.0	0.0	100.0
Total		3	0	5	4	12	14	1	0	39	30.8	69.2	74.4	25.6

R7 and R8 outgoing L1

name	skid	pR7a	pR7b	yR7a	yR7b	pR8a	pR8b	yR8a	yR8b	total	%R7	%R8	%p	%y
Putative L1 col I1 11472941 HL	11472940	0	0	0	0	0	13	0	0	13	0.0	100.0	100.0	0.0
Putative L1 col A1 10108986 MF	10108985	2	0	0	0	8	0	0	0	10	20.0	80.0	100.0	0.0
Putative L1 col H1 11472771 CL	11472770	0	0	0	1	0	0	0	8	9	11.1	88.9	0.0	100.0
Putative L1 col G1 11470179 MF	11470178	0	0	0	0	0	0	6	0	6	0.0	100.0	0.0	100.0
Total		2	0	0	1	8	13	6	8	38	7.9	92.1	60.5	39.5

R7 and R8 outgoing aMe12

name	skid	pR7a	pR7b	yR7a	yR7b	pR8a	pR8b	yR8a	yR8b	total	%R7	%R8	%p	%y
Putative aMe12 VPN ME.R to vACA OLCT bilateral 7038036 JS ECM	7038035	2	0	0	0	15	0	0	0	17	11.8	88.2	100	0
Putative aMe12 VPN ME.R to vACA OLCT bilateral 28842 AA GA	28841	1	1	0	0	0	11	0	0	13	15.4	84.6	100	0
Total		3	1	0	0	15	11	0	0	30	13.3	86.7	100	0

R7 and R8 outgoing Dm

name	skid	pR7a	pR7b	yR7a	yR7b	pR8a	pR8b	yR8a	yR8b	total	%R7	%R8	%p	%y
Putative Dm 11511058 MF	11511057	0	4	0	0	0	4	0	0	8	50.0	50.0	100.0	0.0
Putative Dm 11448963 MF	11448962	2	4	0	0	0	2	0	0	8	75.0	25.0	100.0	0.0
Putative Dm 11474157 CL	11474156	0	0	0	1	0	0	0	5	6	16.7	83.3	0.0	100.0
Putative Dm 10106711 MF	10106710	1	0	3	0	0	0	0	0	4	100.0	0.0	25.0	75.0
Putative Dm 11455002 MF	11455001	0	0	0	3	0	0	0	0	3	100.0	0.0	0.0	100.0
Total		3	8	3	4	0	6	0	5	29	62.1	37.9	58.6	41.4

R7 and R8 outgoing ML-VPN1

name	skid	pR7a	pR7b	yR7a	yR7b	pR8a	pR8b	yR8a	yR8b	total	%R7	%R8	%p	%y
Putative ML_VPN1 11458373 CL	11458372	0	0	0	0	14	0	0	1	15	0	100	93.3	6.7
Putative ML_VPN1 11467871 HL	11467870	0	0	0	0	0	7	0	0	7	0	100	100.0	0.0
Putative ML_VPN1 11466212 CL	11466211	0	0	0	0	5	0	0	0	5	0	100	100.0	0.0
Total		0	0	0	0	19	7	0	1	27	0	100	96.3	3.7

R7 and R8 outgoing C2

name	skid	pR7a	pR7b	yR7a	yR7b	pR8a	pR8b	yR8a	yR8b	total	%R7	%R8	%p	%y
Putative C2 11453788 CL	11453787	0	6	9	4	0	0	0	0	19	100.0	0.0	31.6	68.4
Putative C2 11456777 CL	11456776	1	0	0	0	6	0	0	0	7	14.3	85.7	100.0	0.0
Total		1	6	9	4	6	0	0	0	26	76.9	23.1	50.0	50.0

R7 and R8 outgoing Mt-VPN

name	skid	pR7a	pR7b	yR7a	yR7b	pR8a	pR8b	yR8a	yR8b	total	%R7	%R8	%p	%y
Putative Mt_VPN 11453465 CL	11453464	0	0	5	6	0	0	0	0	11	100.0	0.0	0	100
Putative Mt_VPN 11469482 HL	11469481	0	0	0	0	0	0	4	1	5	0.0	100.0	0	100
Putative Mt_VPN 3401517 MR	14286406	0	0	1	0	0	0	3	0	4	25.0	75.0	0	100
Putative Mt_VPN LP neuron 3509521 CPM	3509520	0	0	0	0	0	3	0	0	3	0.0	100.0	100	0
Total		0	0	6	6	0	3	7	1	23	52.2	47.8	13	87

R7 and R8 outgoing Mti

name	skid	pR7a	pR7b	yR7a	yR7b	pR8a	pR8b	yR8a	yR8b	total	%R7	%R8	%p	%y
Putative Mti 10289206 MF	11666155	0	2	0	0	2	5	0	0	9	22.2	77.8	100.0	0.0
Putative Mti 11481396 CL	11481395	0	0	0	0	0	0	7	0	7	0.0	100.0	0.0	100.0
Putative Mti 11466227 CL	11466226	0	0	0	0	3	0	0	0	3	0.0	100.0	100.0	0.0
Total		0	2	0	0	5	5	7	0	19	10.5	89.5	63.2	36.8

R7 and R8 outgoing Tm5a-like

name	skid	pR7a	pR7b	yR7a	yR7b	pR8a	pR8b	yR8a	yR8b	total	%R7	%R8	%p	%y
Putative Tm5a-like 11481725 MF	11481724	0	0	1	0	0	0	16	0	17	5.9	94.1	0	100
Total		0	0	1	0	0	0	16	0	17	5.9	94.1	0	100

R7 and R8 outgoing TmY10

name	skid	pR7a	pR7b	yR7a	yR7b	pR8a	pR8b	yR8a	yR8b	total	%R7	%R8	%p	%y
Putative TmY10 11449412 HL	11449411	0	1	0	0	0	6	0	0	7	14.3	85.7	100	0
Total		0	1	0	0	0	6	0	0	7	14.3	85.7	100	0

R7 and R8 outgoing Mi10

name	skid	pR7a	pR7b	yR7a	yR7b	pR8a	pR8b	yR8a	yR8b	total	%R7	%R8	%p	%y
Putative Mi10 11481717 MF	11481716	0	0	0	0	0	0	5	0	5	0	100	0	100
Total		0	0	0	0	0	0	5	0	5	0	100	0	100

R7 and R8 outgoing Mi

name	skid	pR7a	pR7b	yR7a	yR7b	pR8a	pR8b	yR8a	yR8b	total	%R7	%R8	%p	%y
Putative Mi 11453950 HL	11453949	0	2	0	1	0	0	0	0	3	100	0	66.7	33.3
Total		0	2	0	1	0	0	0	0	3	100	0	66.7	33.3

R7 and R8 outgoing C3

name	skid	pR7a	pR7b	yR7a	yR7b	pR8a	pR8b	yR8a	yR8b	total	%R7	%R8	%p	%y
Putative C3 11471582 CL	11471581	0	0	0	0	0	0	0	3	3	0	100	0	100
Total		0	0	0	0	0	0	0	3	3	0	100	0	100

R7 and R8 outgoing Identified-<3

name	skid	pR7a	pR7b	yR7a	yR7b	pR8a	pR8b	yR8a	yR8b	total	%R7	%R8	%p	%y
Putative Tm 11469206 HL	11469205	0	0	0	0	0	2	0	0	2	0.0	100.0	100.0	0.0
Putative Tm 11458353 MF	11458352	0	0	0	0	2	0	0	0	2	0.0	100.0	100.0	0.0
Putative Tm 11448401 MF	11448400	0	0	2	0	0	0	0	0	2	100.0	0.0	0.0	100.0
Putative Tm 10649077 MF	10649076	0	0	0	0	0	0	2	0	2	0.0	100.0	0.0	100.0
Putative Mt_VPN 4711709 ME	4711708	0	0	0	0	0	2	0	0	2	0.0	100.0	100.0	0.0
Putative ML_VPN2 11474247 CL	11671334	0	0	0	0	0	0	0	2	2	0.0	100.0	0.0	100.0
Putative MeTu 14838260 AT	14838259	0	0	2	0	0	0	0	0	2	100.0	0.0	0.0	100.0
Putative Dm2 11449695 HL	11449694	0	2	0	0	0	0	0	0	2	100.0	0.0	100.0	0.0
Putative Dm11 11455072 HL	11455071	0	0	0	2	0	0	0	0	2	100.0	0.0	0.0	100.0
Putative Dm 10562975 MF	10562974	0	2	0	0	0	0	0	0	2	100.0	0.0	100.0	0.0
Putative C2 11472486 HL	11472485	0	0	0	0	0	2	0	0	2	0.0	100.0	100.0	0.0
Putative Tm3 14359935 CL	14359934	0	0	1	0	0	0	0	0	1	100.0	0.0	0.0	100.0
Putative Tm1 8942830690 CL	14653782	0	0	0	0	1	0	0	0	1	0.0	100.0	100.0	0.0
Putative Tm 9725279989 CL	14767205	0	0	0	1	0	0	0	0	1	100.0	0.0	0.0	100.0
Putative Tm 15805232 HL	15805231	0	0	1	0	0	0	0	0	1	100.0	0.0	0.0	100.0
Putative Tm 11749586 HL	11749585	0	0	1	0	0	0	0	0	1	100.0	0.0	0.0	100.0
Putative Tm 10657485 HL	10657484	0	0	1	0	0	0	0	0	1	100.0	0.0	0.0	100.0
Putative Tm 10547529 MF	10547528	0	0	1	0	0	0	0	0	1	100.0	0.0	0.0	100.0
Putative T1 11454504 MF	11454503	0	0	0	0	1	0	0	0	1	0.0	100.0	100.0	0.0
Putative Mi14 8421422362 CL	14746351	0	0	0	0	1	0	0	0	1	0.0	100.0	100.0	0.0
Putative Mi1 8809115577 CL	14880472	0	0	0	0	1	0	0	0	1	0.0	100.0	100.0	0.0
Putative MeTu 10656251 MF	10656250	0	0	0	1	0	0	0	0	1	100.0	0.0	0.0	100.0
Putative LaWF1 8286417189 CL	14624795	0	0	0	0	0	1	0	0	1	0.0	100.0	100.0	0.0
Putative Dm 11723760 MF	11723759	0	0	0	0	1	0	0	0	1	0.0	100.0	100.0	0.0
Putative Dm 10638023 HL	10638022	0	0	1	0	0	0	0	0	1	100.0	0.0	0.0	100.0
Putative Dm 10537971 MF	10537970	0	1	0	0	0	0	0	0	1	100.0	0.0	100.0	0.0
Total		0	5	10	4	7	7	2	2	37	51.4	48.6	51.4	48.6

R7 and R8 outgoing Unidentified->=3

name	skid	pR7a	pR7b	yR7a	yR7b	pR8a	pR8b	yR8a	yR8b	total	%R7	%R8	%p	%y
Neuron 15934960 HL	15934959	0	0	0	0	0	5	0	0	5	0	100	100.0	0.0
neuron 11652951 HL	11469649	0	0	0	0	0	0	2	1	3	0	100	0.0	100.0
Total		0	0	0	0	0	5	2	1	8	0	100	62.5	37.5

R7 and R8 outgoing Unidentified-<3

name	skid	pR7a	pR7b	yR7a	yR7b	pR8a	pR8b	yR8a	yR8b	total	%R7	%R8	%p	%y
neuron 8674787766 HL	15942569	0	0	0	0	0	0	0	2	2	0.0	100.0	0.0	100.0
neuron 8454892 SMA	8454891	0	0	0	0	0	0	0	2	2	0.0	100.0	0.0	100.0
Neuron 217388042 HL	15984291	0	2	0	0	0	0	0	0	2	100.0	0.0	100.0	0.0
neuron 17131131 MF	17131130	0	0	0	0	0	0	2	0	2	0.0	100.0	0.0	100.0
neuron 11483242 HL	11483241	0	0	0	0	0	0	2	0	2	0.0	100.0	0.0	100.0
neuron 11470831 HL	11470830	0	0	0	0	0	2	0	0	2	0.0	100.0	100.0	0.0
neuron 11326962 CL	11326961	0	0	1	0	0	0	1	0	2	50.0	50.0	0.0	100.0
neuron 10563217 MF	10563216	0	2	0	0	0	0	0	0	2	100.0	0.0	100.0	0.0
neuron 17159865	17159864	0	0	0	0	1	0	0	0	1	0.0	100.0	100.0	0.0
neuron 17131177 MF	17131176	1	0	0	0	0	0	0	0	1	100.0	0.0	100.0	0.0
Neuron 15901693	15901692	0	0	1	0	0	0	0	0	1	100.0	0.0	0.0	100.0
Neuron 11598593	11598592	0	0	0	0	1	0	0	0	1	0.0	100.0	100.0	0.0
neuron 11512294 HL	11512293	0	0	0	0	0	1	0	0	1	0.0	100.0	100.0	0.0
neuron 11511048	11511047	0	1	0	0	0	0	0	0	1	100.0	0.0	100.0	0.0
neuron 11481388	11481387	0	0	0	0	0	0	1	0	1	0.0	100.0	0.0	100.0
neuron 11480446	11480445	0	0	0	0	0	0	1	0	1	0.0	100.0	0.0	100.0
neuron 11475567	11475566	0	0	0	0	0	0	1	0	1	0.0	100.0	0.0	100.0
neuron 11474876	11474875	0	0	0	0	0	0	1	0	1	0.0	100.0	0.0	100.0
neuron 11474415	11474414	0	0	0	0	0	0	0	1	1	0.0	100.0	0.0	100.0
neuron 11474213 CL	11474212	0	0	0	0	0	0	0	1	1	0.0	100.0	0.0	100.0
neuron 11474053	11474052	0	0	0	0	0	0	1	0	1	0.0	100.0	0.0	100.0
neuron 11474033	11474032	0	0	0	0	0	0	1	0	1	0.0	100.0	0.0	100.0
neuron 11474028	11474027	0	0	0	0	0	0	1	0	1	0.0	100.0	0.0	100.0
neuron 11473958	11473957	0	0	0	0	0	0	1	0	1	0.0	100.0	0.0	100.0
neuron 11473474 CL	11473473	0	0	0	0	0	0	0	1	1	0.0	100.0	0.0	100.0
neuron 11472913	11472912	0	0	0	0	0	0	1	0	1	0.0	100.0	0.0	100.0
neuron 11472423	11472422	0	0	0	0	0	0	0	1	1	0.0	100.0	0.0	100.0
neuron 11471813 HL	11471812	0	0	0	0	0	1	0	0	1	0.0	100.0	100.0	0.0
neuron 11471528	11471527	0	0	0	0	0	1	0	0	1	0.0	100.0	100.0	0.0
neuron 11469592	11469591	0	0	0	0	0	1	0	0	1	0.0	100.0	100.0	0.0
neuron 11469561	11469560	0	0	0	0	0	0	1	0	1	0.0	100.0	0.0	100.0
neuron 11468662	11468661	0	0	0	0	0	0	0	1	1	0.0	100.0	0.0	100.0
neuron 11467899	11467898	0	0	0	0	0	1	0	0	1	0.0	100.0	100.0	0.0
neuron 11467356	11467355	0	0	0	0	0	1	0	0	1	0.0	100.0	100.0	0.0
neuron 11459213	11459212	0	0	0	0	1	0	0	0	1	0.0	100.0	100.0	0.0
neuron 11459079	11459078	0	0	0	0	1	0	0	0	1	0.0	100.0	100.0	0.0
neuron 11458409	11458408	0	0	0	0	1	0	0	0	1	0.0	100.0	100.0	0.0
neuron 11458393 CL	11458392	0	0	0	0	1	0	0	0	1	0.0	100.0	100.0	0.0
neuron 11458262	11458261	0	0	0	0	1	0	0	0	1	0.0	100.0	100.0	0.0
neuron 11458257	11458256	0	0	0	0	1	0	0	0	1	0.0	100.0	100.0	0.0
neuron 11458247	11458246	0	0	0	0	1	0	0	0	1	0.0	100.0	100.0	0.0
neuron 11458187	11458186	0	0	0	0	1	0	0	0	1	0.0	100.0	100.0	0.0
neuron 11458100	11458099	0	0	0	0	1	0	0	0	1	0.0	100.0	100.0	0.0
neuron 11458095	11458094	0	0	0	0	1	0	0	0	1	0.0	100.0	100.0	0.0
neuron 11457627 MF	11457626	0	0	0	0	1	0	0	0	1	0.0	100.0	100.0	0.0
neuron 11457032 MF	11457031	0	0	0	0	1	0	0	0	1	0.0	100.0	100.0	0.0
neuron 11455152	11455151	0	0	0	1	0	0	0	0	1	100.0	0.0	0.0	100.0
neuron 11454942	11454941	0	0	0	1	0	0	0	0	1	100.0	0.0	0.0	100.0
neuron 11454742	11454741	0	0	0	1	0	0	0	0	1	100.0	0.0	0.0	100.0
neuron 11453858	11453857	0	0	0	1	0	0	0	0	1	100.0	0.0	0.0	100.0
neuron 11453555	11453554	0	0	0	1	0	0	0	0	1	100.0	0.0	0.0	100.0
neuron 11453195	11453194	0	0	0	1	0	0	0	0	1	100.0	0.0	0.0	100.0
neuron 11452412	11452411	0	0	1	0	0	0	0	0	1	100.0	0.0	0.0	100.0
neuron 11449753	11449752	0	1	0	0	0	0	0	0	1	100.0	0.0	100.0	0.0
neuron 11448908	11448907	0	1	0	0	0	0	0	0	1	100.0	0.0	100.0	0.0
neuron 11448383	11448382	0	0	1	0	0	0	0	0	1	100.0	0.0	0.0	100.0
neuron 11448211	11448210	0	0	1	0	0	0	0	0	1	100.0	0.0	0.0	100.0
neuron 11448038	11448037	0	0	1	0	0	0	0	0	1	100.0	0.0	0.0	100.0
neuron 11447605	11447604	0	0	1	0	0	0	0	0	1	100.0	0.0	0.0	100.0
neuron 11447258 HL	11447257	0	0	1	0	0	0	0	0	1	100.0	0.0	0.0	100.0
neuron 11446424 HL	11446423	1	0	0	0	0	0	0	0	1	100.0	0.0	100.0	0.0
Google: 9334377939 HL	15901648	0	0	1	0	0	0	0	0	1	100.0	0.0	0.0	100.0
Total		2	7	9	6	14	8	15	9	70	34.3	65.7	44.3	55.7

R7 and R8 incoming Dm9

name	skid	pR7a	pR7b	yR7a	yR7b	pR8a	pR8b	yR8a	yR8b	total	%R7	%R8	%p	%y
Putative Dm9 11452428 MF	11452427	45	35	39	35	33	36	29	29	281	54.8	45.2	53.0	47.0
Putative Dm9 11447062 CL	11447061	2	0	9	0	0	0	7	0	18	61.1	38.9	11.1	88.9
Putative Dm9 11450496 CL	11450495	0	0	3	4	0	0	2	3	12	58.3	41.7	0.0	100.0
Putative Dm9 11484680 MF	11484679	4	0	0	0	5	0	0	0	9	44.4	55.6	100.0	0.0
Putative Dm9 11454715 CL	11454714	0	0	0	2	0	3	0	2	7	28.6	71.4	42.9	57.1
Putative Dm9 11444387 HL	11444386	0	4	0	0	0	2	0	0	6	66.7	33.3	100.0	0.0
Total		51	39	51	41	38	41	38	34	333	54.7	45.3	50.8	49.2

R7 and R8 incoming R8

name	skid	pR7a	pR7b	yR7a	yR7b	pR8a	pR8b	yR8a	yR8b	total	%R7	%R8	%p	%y
Putative R8 col G1 10629255 MF	10629254	0	0	46	0	0	0	0	0	46	100.0	0.0	0.0	100.0
Putative R8 col A1 10086692 MF	10086691	39	0	0	0	1	0	0	0	40	97.5	2.5	100.0	0.0
Putative R8 col I1 11466409 HL	11466408	0	36	0	0	0	0	0	0	36	100.0	0.0	100.0	0.0
Putative R8 col H1 11468319 CL	11468318	0	0	0	36	0	0	0	0	36	100.0	0.0	0.0	100.0
Total		39	36	46	36	1	0	0	0	158	99.4	0.6	48.1	51.9

R7 and R8 incoming R7

name	skid	pR7a	pR7b	yR7a	yR7b	pR8a	pR8b	yR8a	yR8b	total	%R7	%R8	%p	%y
Putative R7 col A1 10082583 MF	10082582	0	0	0	0	20	0	0	0	20	0	100	100.0	0.0
Putative R7 col H1 10653594 MF	10653593	0	0	0	0	0	0	0	19	19	0	100	0.0	100.0
Putative R7 col I1 10538511 MF	10538510	0	0	0	0	0	11	0	0	11	0	100	100.0	0.0
Putative R7 col G1 10585941 MF	10585940	0	0	0	0	0	0	7	0	7	0	100	0.0	100.0
Total		0	0	0	0	20	11	7	19	57	0	100	54.4	45.6

R7 and R8 incoming Mt-VPN

name	skid	pR7a	pR7b	yR7a	yR7b	pR8a	pR8b	yR8a	yR8b	total	%R7	%R8	%p	%y
Putative Mt_VPN 3401517 MR	14286406	0	0	2	0	0	0	3	0	5	40	60	0	100
Total		0	0	2	0	0	0	3	0	5	40	60	0	100

R7 and R8 incoming C2

name	skid	pR7a	pR7b	yR7a	yR7b	pR8a	pR8b	yR8a	yR8b	total	%R7	%R8	%p	%y
Putative C2 11453788 CL	11453787	0	2	1	2	0	0	0	0	5	100	0	40	60
Total		0	2	1	2	0	0	0	0	5	100	0	40	60

R7 and R8 incoming L3

name	skid	pR7a	pR7b	yR7a	yR7b	pR8a	pR8b	yR8a	yR8b	total	%R7	%R8	%p	%y
Putative L3 col A1 11445252 MF	11445251	0	0	0	0	4	0	0	0	4	0	100	100	0
Total		0	0	0	0	4	0	0	0	4	0	100	100	0

R7 and R8 incoming Identified-<3

name	skid	pR7a	pR7b	yR7a	yR7b	pR8a	pR8b	yR8a	yR8b	total	%R7	%R8	%p	%y
Putative Dm8 10109587 MF	10109586	2	0	0	0	0	0	0	0	2	100.0	0.0	100.0	0.0
Putative R7 10653781 MF	10653780	0	0	0	2	0	0	0	0	2	100.0	0.0	0.0	100.0
Putative L3 col I1 11448597 HL	11448596	0	0	0	0	0	2	0	0	2	0.0	100.0	100.0	0.0
Putative C2 11472486 HL	11472485	0	0	0	0	0	2	0	0	2	0.0	100.0	100.0	0.0
Putative Mi15 11453819 CL	11453818	0	0	0	0	0	0	2	0	2	0.0	100.0	0.0	100.0
Putative L3 col H1 11450470 CL	11450469	0	0	0	0	0	0	0	2	2	0.0	100.0	0.0	100.0
Putative Mi15 11450568 CL	11450567	0	0	0	0	0	0	0	2	2	0.0	100.0	0.0	100.0
Putative C2 11456777 CL	11456776	1	0	0	0	0	0	0	0	1	100.0	0.0	100.0	0.0
Putative Mi15 11484381 HL	11484380	0	1	0	0	0	0	0	0	1	100.0	0.0	100.0	0.0
Putative Tm5b-like 11447921 MF	11447920	0	0	0	0	0	0	1	0	1	0.0	100.0	0.0	100.0
Putative Dm11 11450454 CL	11450453	0	0	0	0	0	0	0	1	1	0.0	100.0	0.0	100.0
Total		3	1	0	2	0	4	3	5	18	33.3	66.7	44.4	55.6

R7 and R8 incoming Unidentified-<3

name	skid	pR7a	pR7b	yR7a	yR7b	pR8a	pR8b	yR8a	yR8b	total	%R7	%R8	%p	%y
neuron 11456493	11456492	0	1	0	0	0	0	0	0	1	100	0	100	0
neuron 11679982	11679981	0	0	1	0	0	0	0	0	1	100	0	0	100
neuron 11472423	11472422	0	0	0	1	0	0	0	0	1	100	0	0	100
neuron 11466245	11466244	0	0	0	0	1	0	0	0	1	0	100	100	0
Total		0	1	1	1	1	0	0	0	4	75	25	50	50

R7-DRA and R8-DRA outgoing Dm-DRA1

name	skid	R7-DRA-a	R7-DRA-b	R7-DRA-c	R8-DRA-a	R8-DRA-b	R8-DRA-c	total	%R7-DRA	%R8-DRA
Putative Dm-DRA1 10440161 TO	10440160	0	0	21	0	0	0	21	100	0
Putative Dm-DRA1 11896102 EK	11896101	0	18	0	0	0	0	18	100	0
Putative Dm-DRA1 16766813 EK	16766812	0	0	16	0	0	0	16	100	0
Putative Dm-DRA1 12106450 TO	12106449	16	0	0	0	0	0	16	100	0
Putative Dm-DRA1 10247371 TO	10247370	5	10	0	0	0	0	15	100	0
Putative Dm-DRA1 17156428 EK	17156427	0	0	14	0	0	0	14	100	0
Putative Dm-DRA1 17155322 EK	17155321	0	0	13	0	0	0	13	100	0
Putative Dm-DRA1 14065299 GS-EK	14065298	4	9	0	0	0	0	13	100	0
Putative Dm-DRA1 10265807 TO EK	10265806	8	5	0	0	0	0	13	100	0
Putative Dm-DRA1 11903983 GS	11903982	0	10	0	0	0	0	10	100	0
Putative Dm-DRA1 11992844 EK	11992843	9	0	0	0	0	0	9	100	0
Putative Dm-DRA1 11141230 GS	11141229	0	9	0	0	0	0	9	100	0
Putative Dm-DRA1 13979722 EK	13979721	6	2	0	0	0	0	8	100	0
Putative Dm-DRA1 11993799 EK	11993798	7	0	0	0	0	0	7	100	0
Putative Dm-DRA1 11993077 EK	11993076	7	0	0	0	0	0	7	100	0
Putative Dm-DRA1 13607721 EK	13607720	6	0	0	0	0	0	6	100	0
Putative Dm-DRA1 11769715 TO EK	11769714	6	0	0	0	0	0	6	100	0
Putative Dm-DRA1 17170764 EK	17170763	0	0	5	0	0	0	5	100	0
Putative Dm-DRA1 15976818 EK	15976817	0	3	0	0	0	0	3	100	0
Putative Dm-DRA1 11993696 EK	11993695	3	0	0	0	0	0	3	100	0
Total		77	66	69	0	0	0	212	100	0

R7-DRA and R8-DRA outgoing Dm9

name	skid	R7-DRA-a	R7-DRA-b	R7-DRA-c	R8-DRA-a	R8-DRA-b	R8-DRA-c	total	%R7-DRA	%R8-DRA
Putative Dm9 12013130 EK	12013129	0	0	31	0	0	36	67	46.3	53.7
Putative Dm9 11916196 EK	11916195	26	0	0	36	0	0	62	41.9	58.1
Putative Dm9 10657501 GS	10657500	0	17	0	0	35	0	52	32.7	67.3
Putative Dm9 14310275 GS	14310274	0	16	0	0	5	0	21	76.2	23.8
Putative Dm9 14933803 EK	14933802	0	3	0	0	1	0	4	75.0	25.0
Putative Dm9 10655889 EK	10655888	0	2	0	0	2	0	4	50.0	50.0
Total		26	38	31	36	43	36	210	45.2	54.8

R7-DRA and R8-DRA outgoing MeTu-DRA

name	skid	R7-DRA-a	R7-DRA-b	R7-DRA-c	R8-DRA-a	R8-DRA-b	R8-DRA-c	total	%R7-DRA	%R8-DRA
Putative MeTu_DRA 11995074 EK	11995073	14	0	0	0	0	0	14	100	0
Putative MeTu_DRA 11994449 EK	11994448	12	0	0	0	0	0	12	100	0
Putative MeTu_DRA 11908711 GS	11908710	0	10	0	0	0	0	10	100	0
Putative MeTu_DRA 11695294 PA	11695293	0	0	10	0	0	0	10	100	0
Putative MeTu_DRA 10737979 TO	15749047	10	0	0	0	0	0	10	100	0
Putative MeTu_DRA 12106384 EK	12106383	0	0	9	0	0	0	9	100	0
Putative MeTu_DRA 11994564 EK	11994563	8	0	0	0	0	0	8	100	0
Putative MeTu_DRA 10438340 TO	10438339	0	0	8	0	0	0	8	100	0
Putative MeTu_DRA 7749081264 EK	15952007	0	0	7	0	0	0	7	100	0
Putative MeTu_DRA 13136157 EK	13136156	0	7	0	0	0	0	7	100	0
Putative MeTu_DRA 11995103 EK	11995102	7	0	0	0	0	0	7	100	0
Putative MeTu_DRA 15616478 EK	15616477	0	6	0	0	0	0	6	100	0
Putative MeTu_DRA 15600899 EK	15600898	0	6	0	0	0	0	6	100	0
Putative MeTu_DRA 11993031 EK	11993030	6	0	0	0	0	0	6	100	0
Putative MeTu_DRA 10479162 TO	15698953	0	6	0	0	0	0	6	100	0
Putative MeTu_DRA 12127370 TO	12127369	5	0	0	0	0	0	5	100	0
Putative MeTu_DRA 11993383 EK	11993382	5	0	0	0	0	0	5	100	0
Putative MeTu_DRA 11992933 EK	11992932	5	0	0	0	0	0	5	100	0
Putative MeTu_DRA 6844453075 EK	15949451	0	0	4	0	0	0	4	100	0
Putative MeTu_DRA 17250637 EK	17250636	0	0	4	0	0	0	4	100	0
Putative MeTu_DRA 14889232 EK	14889231	0	0	4	0	0	0	4	100	0
Putative MeTu_DRA 11995359 EK	11995358	4	0	0	0	0	0	4	100	0
Putative MeTu_DRA 11993353 EK	11993352	4	0	0	0	0	0	4	100	0
Putative MeTu_DRA 11993315 EK	11993314	2	2	0	0	0	0	4	100	0
Putative MeTu_DRA 11908744 GS	11908743	0	4	0	0	0	0	4	100	0
Putative MeTu_DRA 9706730253 EK	14864504	0	3	0	0	0	0	3	100	0
Putative MeTu_DRA 14936635 EK	14936634	0	0	3	0	0	0	3	100	0
Putative MeTu_DRA 11993065 EK	11993064	3	0	0	0	0	0	3	100	0
Putative MeTu_DRA 11908669 GS	11908668	0	3	0	0	0	0	3	100	0
Putative MeTu_DRA 11903993 EK	11903992	0	3	0	0	0	0	3	100	0
Total		85	50	49	0	0	0	184	100	0

R7-DRA and R8-DRA outgoing R7-DRA

name	skid	R7-DRA-a	R7-DRA-b	R7-DRA-c	R8-DRA-a	R8-DRA-b	R8-DRA-c	total	%R7-DRA	%R8-DRA
Putative R7_DRA 11728780 TO	11728779	0	0	1	0	0	37	38	2.6	97.4
Putative R7_DRA 10300950 TO	10300949	0	0	0	0	38	0	38	0.0	100.0
Putative R7_DRA 10191736 TO	10191735	0	0	0	33	0	0	33	0.0	100.0
Total		0	0	1	33	38	37	109	0.9	99.1

R7-DRA and R8-DRA outgoing Dm-DRA2

name	skid	R7-DRA-a	R7-DRA-b	R7-DRA-c	R8-DRA-a	R8-DRA-b	R8-DRA-c	total	%R7-DRA	%R8-DRA
Putative Dm-DRA2 10411789 TO	10411788	0	1	0	0	31	0	32	3.1	96.9
Putative Dm-DRA2 10483849 TO	10483848	0	0	0	19	0	0	19	0.0	100.0
Putative Dm-DRA2 11710980 TO	11710979	0	0	1	0	0	12	13	7.7	92.3
Putative Dm-DRA2 10449077 TO	10449076	0	0	0	9	0	0	9	0.0	100.0
Putative Dm-DRA2 10484772 TO	10484771	0	0	0	0	8	0	8	0.0	100.0
Putative Dm-DRA2 17163677 EK	17163676	0	0	0	0	0	7	7	0.0	100.0
Putative Dm-DRA2 13262260 EK	13262259	0	0	0	7	0	0	7	0.0	100.0
Putative Dm-DRA2 14311484 GS	14311483	0	1	0	0	5	0	6	16.7	83.3
Putative Dm-DRA2 11918437 EK	11918436	0	0	0	5	0	0	5	0.0	100.0
Total		0	2	1	40	44	19	106	2.8	97.2

R7-DRA and R8-DRA outgoing Dm2

name	skid	R7-DRA-a	R7-DRA-b	R7-DRA-c	R8-DRA-a	R8-DRA-b	R8-DRA-c	total	%R7-DRA	%R8-DRA
Putative Dm2 17162383 EK	17162382	0	0	6	0	0	15	21	28.6	71.4
Putative Dm2 11918131 EK	11918130	5	0	0	7	0	0	12	41.7	58.3
Putative Dm2 14065354 EK	14065353	0	10	0	0	1	0	11	90.9	9.1
Putative Dm2 10655669 TO	10655668	0	0	0	0	4	0	4	0.0	100.0
Total		5	10	6	7	5	15	48	43.8	56.2

R7-DRA and R8-DRA outgoing R8-DRA

name	skid	R7-DRA-a	R7-DRA-b	R7-DRA-c	R8-DRA-a	R8-DRA-b	R8-DRA-c	total	%R7-DRA	%R8-DRA
Putative R8_DRA 10300964 TO	10300963	0	16	0	0	0	0	16	100.0	0.0
Putative R8_DRA 11728828 TO	11728827	0	0	15	0	0	0	15	100.0	0.0
Putative R8_DRA 10190509 TO	10190508	10	0	0	1	0	0	11	90.9	9.1
Total		10	16	15	1	0	0	42	97.6	2.4

R7-DRA and R8-DRA outgoing Mi15

name	skid	R7-DRA-a	R7-DRA-b	R7-DRA-c	R8-DRA-a	R8-DRA-b	R8-DRA-c	total	%R7-DRA	%R8-DRA
Putative Mi15 10655927 TO	10655926	0	8	0	0	10	0	18	44.4	55.6
Putative Mi15 12017151 EK	12017150	0	0	5	0	0	5	10	50.0	50.0
Putative Mi15 11916191 EK	11916190	4	0	0	3	0	0	7	57.1	42.9
Putative Mi15 12002140 EK	12002139	0	0	4	0	0	0	4	100.0	0.0
Total		4	8	9	3	10	5	39	53.8	46.2

R7-DRA and R8-DRA outgoing Mti-DRA-1

name	skid	R7-DRA-a	R7-DRA-b	R7-DRA-c	R8-DRA-a	R8-DRA-b	R8-DRA-c	total	%R7-DRA	%R8-DRA
Putative Mti_DRA_1 11993472 EK	11993471	6	7	4	0	0	0	17	100	0
Putative Mti_DRA_1 14430737 EK	14430736	4	2	1	0	0	0	7	100	0
Putative Mti_DRA_1 11994581 EK	11994580	2	0	2	0	0	0	4	100	0
Putative Mti_DRA_1 11995267 EK	11995266	1	0	2	0	0	0	3	100	0
Putative Mti_DRA_1 11686948 EK	11686947	0	1	2	0	0	0	3	100	0
Putative Mti_DRA_1 10474754 PA	10474753	0	0	3	0	0	0	3	100	0
Total		13	10	14	0	0	0	37	100	0

R7-DRA and R8-DRA outgoing MeMe-DRA

name	skid	R7-DRA-a	R7-DRA-b	R7-DRA-c	R8-DRA-a	R8-DRA-b	R8-DRA-c	total	%R7-DRA	%R8-DRA
Putative MeMe_DRA 10439443 EK	10439442	6	0	21	0	0	0	27	100	0
Putative MeMe_DRA 11993544 EK	11993543	9	0	0	0	0	0	9	100	0
Total		15	0	21	0	0	0	36	100	0

R7-DRA and R8-DRA outgoing L3

name	skid	R7-DRA-a	R7-DRA-b	R7-DRA-c	R8-DRA-a	R8-DRA-b	R8-DRA-c	total	%R7-DRA	%R8-DRA
Putative L3 12018070 EK	12018069	0	0	7	0	0	6	13	53.8	46.2
Putative L3 10653986 EK	10653985	0	5	0	0	7	0	12	41.7	58.3
Putative L3 11917228 EK	11917227	4	0	0	3	0	0	7	57.1	42.9
Total		4	5	7	3	7	6	32	50.0	50.0

R7-DRA and R8-DRA outgoing VPN-DRA

name	skid	R7-DRA-a	R7-DRA-b	R7-DRA-c	R8-DRA-a	R8-DRA-b	R8-DRA-c	total	%R7-DRA	%R8-DRA
Putative VPN_DRA ME.R 11992908 EK	11992907	2	0	6	0	0	0	8	100	0
Putative VPN_DRA ME.R 11904074 GS EK	11904073	5	2	0	0	0	0	7	100	0
Putative VPN_DRA ME.R 4271777 EK	4271776	1	4	0	0	0	0	5	100	0
Putative VPN_DRA ME.R 17165716 EK	17165715	0	0	4	0	0	0	4	100	0
Putative VPN_DRA ME.R 8541155621 EK	15969128	3	0	0	0	0	0	3	100	0
Putative VPN_DRA ME.R 16886431 PA	16886430	0	0	3	0	0	0	3	100	0
Total		11	6	13	0	0	0	30	100	0

R7-DRA and R8-DRA outgoing L1

name	skid	R7-DRA-a	R7-DRA-b	R7-DRA-c	R8-DRA-a	R8-DRA-b	R8-DRA-c	total	%R7-DRA	%R8-DRA
Putative L1 10654563 EK-GS	10654562	0	3	0	0	5	0	8	37.5	62.5
Putative L1 11915726 EK	11915725	3	0	0	4	0	0	7	42.9	57.1
Putative L1 12019445 EK	12019444	0	0	3	0	0	0	3	100.0	0.0
Total		3	3	3	4	5	0	18	50.0	50.0

R7-DRA and R8-DRA outgoing Tm20

name	skid	R7-DRA-a	R7-DRA-b	R7-DRA-c	R8-DRA-a	R8-DRA-b	R8-DRA-c	total	%R7-DRA	%R8-DRA
Putative Tm20 12018770 EK	12018769	0	0	0	0	0	8	8	0	100
Putative Tm20 11918620 EK	11918619	0	0	0	4	0	0	4	0	100
Putative Tm20 10655482 EK	10655481	0	0	0	0	4	0	4	0	100
Total		0	0	0	4	4	8	16	0	100

R7-DRA and R8-DRA outgoing Mti-DRA-2

name	skid	R7-DRA-a	R7-DRA-b	R7-DRA-c	R8-DRA-a	R8-DRA-b	R8-DRA-c	total	%R7-DRA	%R8-DRA
Putative Mti_DRA_2 11995289 EK	11995288	5	0	0	0	0	0	5	100	0
Putative Mti_DRA_2 11693572 PA	11693571	0	0	4	0	0	0	4	100	0
Putative Mti_DRA_2 11993445 EK	11993444	3	0	0	0	0	0	3	100	0
Putative Mti_DRA_2 11903803 GS	11903802	0	3	0	0	0	0	3	100	0
Total		8	3	4	0	0	0	15	100	0

R7-DRA and R8-DRA outgoing Mi1

name	skid	R7-DRA-a	R7-DRA-b	R7-DRA-c	R8-DRA-a	R8-DRA-b	R8-DRA-c	total	%R7-DRA	%R8-DRA
Putative Mi1 11829851 HL	11829850	0	0	1	0	0	5	6	16.7	83.3
Putative Mi1 13294452 EK	13294451	3	0	0	2	0	0	5	60.0	40.0
Putative Mi1 11660604824 CL	14811021	0	2	0	0	2	0	4	50.0	50.0
Total		3	2	1	2	2	5	15	40.0	60.0

R7-DRA and R8-DRA outgoing MeTu

name	skid	R7-DRA-a	R7-DRA-b	R7-DRA-c	R8-DRA-a	R8-DRA-b	R8-DRA-c	total	%R7-DRA	%R8-DRA
Putative MeTu 16730683 AT	16730682	0	0	8	0	0	0	8	100	0
Putative MeTu 14888321 AT	14888320	0	0	5	0	0	0	5	100	0
Total		0	0	13	0	0	0	13	100	0

R7-DRA and R8-DRA outgoing Tm5-like

name	skid	R7-DRA-a	R7-DRA-b	R7-DRA-c	R8-DRA-a	R8-DRA-b	R8-DRA-c	total	%R7-DRA	%R8-DRA
Putative Tm5-like 12016007 EK	12016006	0	0	0	0	0	12	12	0	100
Total		0	0	0	0	0	12	12	0	100

R7-DRA and R8-DRA outgoing Mi9

name	skid	R7-DRA-a	R7-DRA-b	R7-DRA-c	R8-DRA-a	R8-DRA-b	R8-DRA-c	total	%R7-DRA	%R8-DRA
Putative Mi9 12015967 EK	12015966	0	0	0	0	0	9	9	0	100
Putative Mi9 11918591 EK	11918590	0	0	0	3	0	0	3	0	100
Total		0	0	0	3	0	9	12	0	100

R7-DRA and R8-DRA outgoing Dm11

name	skid	R7-DRA-a	R7-DRA-b	R7-DRA-c	R8-DRA-a	R8-DRA-b	R8-DRA-c	total	%R7-DRA	%R8-DRA
Putative Dm11 17161736 EK	17161735	0	0	4	0	0	8	12	33.3	66.7
Total		0	0	4	0	0	8	12	33.3	66.7

R7-DRA and R8-DRA outgoing aMe12

name	skid	R7-DRA-a	R7-DRA-b	R7-DRA-c	R8-DRA-a	R8-DRA-b	R8-DRA-c	total	%R7-DRA	%R8-DRA
Putative aMe12 VPN ME.R to vACA OLCT bilateral 164545 GA ECM	164544	0	1	2	0	1	0	4	75	25
Total		0	1	2	0	1	0	4	75	25

R7-DRA and R8-DRA outgoing TmY

name	skid	R7-DRA-a	R7-DRA-b	R7-DRA-c	R8-DRA-a	R8-DRA-b	R8-DRA-c	total	%R7-DRA	%R8-DRA
Putative TmY 12043438 EK	12043437	0	0	0	3	0	0	3	0	100
Total		0	0	0	3	0	0	3	0	100

R7-DRA and R8-DRA outgoing ML-VPN2

name	skid	R7-DRA-a	R7-DRA-b	R7-DRA-c	R8-DRA-a	R8-DRA-b	R8-DRA-c	total	%R7-DRA	%R8-DRA
Putative ML_VPN2 11995243 EK	11995242	3	0	0	0	0	0	3	100	0
Total		3	0	0	0	0	0	3	100	0

R7-DRA and R8-DRA outgoing C2

name	skid	R7-DRA-a	R7-DRA-b	R7-DRA-c	R8-DRA-a	R8-DRA-b	R8-DRA-c	total	%R7-DRA	%R8-DRA
Putative C2 11906498 GS	11906497	0	2	0	0	1	0	3	66.7	33.3
Total		0	2	0	0	1	0	3	66.7	33.3

R7-DRA and R8-DRA outgoing Identified-<3

name	skid	R7-DRA-a	R7-DRA-b	R7-DRA-c	R8-DRA-a	R8-DRA-b	R8-DRA-c	total	%R7-DRA	%R8-DRA
Putative Tm5-like 12017355 EK	12017354	0	0	2	0	0	0	2	100.0	0.0
Putative Mi9 10655487 EK	10655486	0	0	0	0	2	0	2	0.0	100.0
Putative MeTu_DRA 11993247 EK	15749075	2	0	0	0	0	0	2	100.0	0.0
Putative MeTu 10806612 EK	10806611	2	0	0	0	0	0	2	100.0	0.0
Putative MeTu 10473597 EK	10473596	0	0	2	0	0	0	2	100.0	0.0
Putative L2 12043826 EK	12043825	0	0	2	0	0	0	2	100.0	0.0
Putative L2 11915907 EK	11915906	1	0	0	1	0	0	2	50.0	50.0
Putative Dm2 17254551 EK	17254550	0	0	2	0	0	0	2	100.0	0.0
Putative Dm2 14305319 EK	14305318	0	0	0	2	0	0	2	0.0	100.0
Putative Dm 16110050 EK	16110049	0	2	0	0	0	0	2	100.0	0.0
Putative Dm 11993804 EK	11993803	1	0	0	1	0	0	2	50.0	50.0
Putative Dm 10479098 EK	10479097	2	0	0	0	0	0	2	100.0	0.0
Putative Dm-DRA2 11981476 EK	11981475	0	0	0	2	0	0	2	0.0	100.0
Putative C2 12044213 EK	12044212	0	0	1	0	0	1	2	50.0	50.0
Putative aMe12 VPN ME.R to vACA OLCT bilateral 28842 AA GA	28841	0	2	0	0	0	0	2	100.0	0.0
Putative VPN_DRA ME.R 8656926956 EK	15997281	0	1	0	0	0	0	1	100.0	0.0
Putative VPN_DRA ME.R 16215492 EK	16215491	0	1	0	0	0	0	1	100.0	0.0
Putative VPN_DRA ME.R 12188203 EK	12188202	1	0	0	0	0	0	1	100.0	0.0
Putative VPN_DRA ME.R 10088063089 EK	15984100	1	0	0	0	0	0	1	100.0	0.0
Putative T2 17252542 EK	17252541	0	0	1	0	0	0	1	100.0	0.0
Putative Mt_DRA_2 12109027 EK	12109026	1	0	0	0	0	0	1	100.0	0.0
Putative Mt_DRA_2 11993433 EK	11993432	1	0	0	0	0	0	1	100.0	0.0
Putative Mt_DRA_2 11992771 EK	11992770	1	0	0	0	0	0	1	100.0	0.0
Putative Mi9 11947153 EK	11947152	0	0	0	1	0	0	1	0.0	100.0
Putative Mi4 12045603 EK	12045602	0	0	0	0	1	0	1	0.0	100.0
Putative MeTu_DRA 10820917 EK	10820916	1	0	0	0	0	0	1	100.0	0.0
Putative MeTu_DRA 10755132 EK	10755131	1	0	0	0	0	0	1	100.0	0.0
Putative MeTu 4735813145 EK	15978559	0	1	0	0	0	0	1	100.0	0.0
Putative MeTu 14726393 MF	14726392	1	0	0	0	0	0	1	100.0	0.0
Putative L3 6831613493 EK	15970720	0	1	0	0	0	0	1	100.0	0.0
Putative L3 17253296 EK	17253295	0	0	0	0	0	1	1	0.0	100.0
Putative Dm3 12018854 EK	12018853	0	0	0	0	0	1	1	0.0	100.0
Putative C3 12002623 EK	12002622	0	0	0	0	0	1	1	0.0	100.0
Total		16	8	10	7	3	4	48	70.8	29.2

R7-DRA and R8-DRA outgoing Unidentified->=3

name	skid	R7-DRA-a	R7-DRA-b	R7-DRA-c	R8-DRA-a	R8-DRA-b	R8-DRA-c	total	%R7-DRA	%R8-DRA
Neuron 15007628 (not traceable) EK	15007627	0	5	0	0	0	0	5	100	0
10655594 EK (untraceable)	10655593	0	0	0	0	5	0	5	0	100
Total		0	5	0	0	5	0	10	50	50

R7-DRA and R8-DRA outgoing Unidentified-<3

name	skid	R7-DRA-a	R7-DRA-b	R7-DRA-c	R8-DRA-a	R8-DRA-b	R8-DRA-c	total	%R7-DRA	%R8-DRA
Neuron 15767888	15767887	0	2	0	0	0	0	2	100.0	0.0
Neuron 14867404	14867403	0	2	0	0	0	0	2	100.0	0.0
Neuron 14788296 EK	14788295	0	2	0	0	0	0	2	100.0	0.0
neuron 11994901 EK	11994900	0	0	0	2	0	0	2	0.0	100.0
neuron 11947310 EK (untraceable)	11947309	0	0	0	2	0	0	2	0.0	100.0
neuron 11918119 EK (untraceable)	11918118	0	0	0	2	0	0	2	0.0	100.0
neuron 11918097 EK (untraceable)	11918096	0	0	0	2	0	0	2	0.0	100.0
neuron 11904799 GS (untraceable)	11904798	0	2	0	0	0	0	2	100.0	0.0
neuron 11904043 GS (not traceable)	11904042	0	2	0	0	0	0	2	100.0	0.0
neuron 11903818 GS (not traceable)	11903817	0	2	0	0	0	0	2	100.0	0.0
Neuron 14991389	14991388	0	1	0	0	0	0	1	100.0	0.0
Neuron 14798841	14798840	0	1	0	0	0	0	1	100.0	0.0
Neuron 14315234	14315233	0	0	0	0	1	0	1	0.0	100.0
Neuron 14081457 GS	14081456	0	1	0	0	0	0	1	100.0	0.0
Neuron 13938782	13938781	0	1	0	0	0	0	1	100.0	0.0
Neuron 13433448 EK (untraceable)	13433447	1	0	0	0	0	0	1	100.0	0.0
neuron 13179813 EK	13179812	0	1	0	0	0	0	1	100.0	0.0
neuron 12043433 EK	12043432	0	0	0	1	0	0	1	0.0	100.0
neuron 12019263	12019262	0	0	1	0	0	0	1	100.0	0.0
neuron 12019256	12019255	0	0	0	0	0	1	1	0.0	100.0
neuron 12018755	12018754	0	0	0	0	0	1	1	0.0	100.0
neuron 12017797	12017796	0	0	1	0	0	0	1	100.0	0.0
neuron 12017523	12017522	0	0	1	0	0	0	1	100.0	0.0
neuron 12017236	12017235	0	0	0	0	0	1	1	0.0	100.0
neuron 12017107	12017106	0	0	0	0	0	1	1	0.0	100.0
neuron 12016873	12016872	0	0	0	0	0	1	1	0.0	100.0
neuron 12016687	12016686	0	0	1	0	0	0	1	100.0	0.0
neuron 12016413	12016412	0	0	0	0	0	1	1	0.0	100.0
neuron 12015890	12015889	0	0	0	0	0	1	1	0.0	100.0
neuron 12015018	12015017	0	0	1	0	0	0	1	100.0	0.0
neuron 12014001	12014000	0	0	1	0	0	0	1	100.0	0.0
neuron 12013715	12013714	0	0	1	0	0	0	1	100.0	0.0
neuron 12002539	12002538	0	0	1	0	0	0	1	100.0	0.0
neuron 12002237	12002236	0	0	1	0	0	0	1	100.0	0.0
neuron 12002170	12002169	0	0	1	0	0	0	1	100.0	0.0
neuron 12002160	12002159	0	0	1	0	0	0	1	100.0	0.0
neuron 11995171 EK	11995170	1	0	0	0	0	0	1	100.0	0.0
neuron 11995069 EK	11995068	1	0	0	0	0	0	1	100.0	0.0
neuron 11995028 EK	11995027	1	0	0	0	0	0	1	100.0	0.0
neuron 11994592 EK	11994591	1	0	0	0	0	0	1	100.0	0.0
neuron 11994244 EK	11994243	1	0	0	0	0	0	1	100.0	0.0
neuron 11993824 EK	11993823	1	0	0	0	0	0	1	100.0	0.0
neuron 11992865 EK	11992864	1	0	0	0	0	0	1	100.0	0.0
neuron 11981310 EK	11981309	0	0	0	1	0	0	1	0.0	100.0
neuron 11981299 EK	11981298	0	0	0	1	0	0	1	0.0	100.0
neuron 11908911 GS	11908910	0	0	0	0	1	0	1	0.0	100.0
neuron 11908664	11908663	0	1	0	0	0	0	1	100.0	0.0
neuron 11904702	11904701	0	1	0	0	0	0	1	100.0	0.0
neuron 11904687	11904686	0	1	0	0	0	0	1	100.0	0.0
neuron 11904395	11904394	0	1	0	0	0	0	1	100.0	0.0
neuron 11904241	11904240	0	1	0	0	0	0	1	100.0	0.0
neuron 11904203	11904202	0	1	0	0	0	0	1	100.0	0.0
neuron 11904153	11904152	0	1	0	0	0	0	1	100.0	0.0
neuron 11904033	11904032	0	1	0	0	0	0	1	100.0	0.0
neuron 11903739	11903738	0	1	0	0	0	0	1	100.0	0.0
neuron 11902130	11902129	0	1	0	0	0	0	1	100.0	0.0
neuron 11829995	11829994	0	0	0	0	0	1	1	0.0	100.0
Total		8	27	11	11	2	8	67	68.7	31.3

R7-DRA and R8-DRA incoming Dm9

name	skid	R7-DRA-a	R7-DRA-b	R7-DRA-c	R8-DRA-a	R8-DRA-b	R8-DRA-c	total	%R7-DRA	%R8-DRA
Putative Dm9 11916196 EK	11916195	39	0	0	34	0	0	73	53.4	46.6
Putative Dm9 12013130 EK	12013129	0	0	34	0	0	35	69	49.3	50.7
Putative Dm9 10657501 GS	10657500	0	30	0	0	36	0	66	45.5	54.5
Putative Dm9 10655889 EK	10655888	0	2	0	0	1	0	3	66.7	33.3
Total		39	32	34	34	37	35	211	49.8	50.2

R7-DRA and R8-DRA incoming R8-DRA

name	skid	R7-DRA-a	R7-DRA-b	R7-DRA-c	R8-DRA-a	R8-DRA-b	R8-DRA-c	total	%R7-DRA	%R8-DRA
Putative R8_DRA 10300964 TO	10300963	0	38	0	0	0	0	38	100.0	0.0
Putative R8_DRA 11728828 TO	11728827	0	0	37	0	0	0	37	100.0	0.0
Putative R8_DRA 10190509 TO	10190508	33	0	0	1	0	0	34	97.1	2.9
Total		33	38	37	1	0	0	109	99.1	0.9

R7-DRA and R8-DRA incoming R7-DRA

name	skid	R7-DRA-a	R7-DRA-b	R7-DRA-c	R8-DRA-a	R8-DRA-b	R8-DRA-c	total	%R7-DRA	%R8-DRA
Putative R7_DRA 11728780 TO	11728779	0	0	1	0	0	15	16	6.2	93.8
Putative R7_DRA 10300950 TO	10300949	0	0	0	0	16	0	16	0.0	100.0
Putative R7_DRA 10191736 TO	10191735	0	0	0	10	0	0	10	0.0	100.0
Total		0	0	1	10	16	15	42	2.4	97.6

R7-DRA and R8-DRA incoming C2

name	skid	R7-DRA-a	R7-DRA-b	R7-DRA-c	R8-DRA-a	R8-DRA-b	R8-DRA-c	total	%R7-DRA	%R8-DRA
Putative C2 12044213 EK	12044212	0	0	2	0	0	3	5	40.0	60.0
Putative C2 11906498 GS	11906497	0	2	0	0	1	0	3	66.7	33.3
Total		0	2	2	0	1	3	8	50.0	50.0

R7-DRA and R8-DRA incoming Mi15

name	skid	R7-DRA-a	R7-DRA-b	R7-DRA-c	R8-DRA-a	R8-DRA-b	R8-DRA-c	total	%R7-DRA	%R8-DRA
Putative Mi15 10655927 TO	10655926	0	2	0	0	2	0	4	50	50
Total		0	2	0	0	2	0	4	50	50

R7-DRA and R8-DRA incoming Identified-<3

name	skid	R7-DRA-a	R7-DRA-b	R7-DRA-c	R8-DRA-a	R8-DRA-b	R8-DRA-c	total	%R7-DRA	%R8-DRA
Putative Dm-DRA1 11993077 EK	11993076	1	0	0	0	0	0	1	100.0	0.0
Putative Dm-DRA1 11993696 EK	11993695	1	0	0	0	0	0	1	100.0	0.0
Putative Dm9 14933803 EK	14933802	0	1	0	0	0	0	1	100.0	0.0
Putative L3 12018070 EK	12018069	0	0	1	0	0	0	1	100.0	0.0
Putative Dm-DRA1 16766813 EK	16766812	0	0	1	0	0	0	1	100.0	0.0
Putative Dm-DRA1 17155322 EK	17155321	0	0	1	0	0	0	1	100.0	0.0
Putative L1 11915726 EK	11915725	0	0	0	1	0	0	1	0.0	100.0
Putative Mi15 11916191 EK	11916190	0	0	0	1	0	0	1	0.0	100.0
Putative L3 10653986 EK	10653985	0	0	0	0	1	0	1	0.0	100.0
Total		2	1	3	2	1	0	9	66.7	33.3

R7-DRA and R8-DRA incoming Unidentified-<3

name	skid	R7-DRA-a	R7-DRA-b	R7-DRA-c	R8-DRA-a	R8-DRA-b	R8-DRA-c	total	%R7-DRA	%R8-DRA
neuron 11915809	11915808	1	0	0	1	0	0	2	50.0	50.0
neuron 11903225	11903224	0	1	0	0	0	0	1	100.0	0.0
Total		1	1	0	1	0	0	3	66.7	33.3
