# Supplementary material for: Synaptic targets of photoreceptors specialized to detect color and skylight polarization in Drosophila
Source: eLife. 2021 Dec 16;10:e71858. doi: 10.7554/eLife.71858 (PMC8789284; doi:10.7554/eLife.71858)
Supplement: Supplementary file 2. [file elife-71858-supp2.pdf]

Supplementary File 2: Gallery plots of all seed column R7, R8, R7-DRA and R8-DRA target cells by type

Contents

Central seed column Dm9 5
Central seed column Dm8 6
Central seed column MeTu 9
Central seed column R7 12
Central seed column Tm5c 13
Central seed column Tm20 15
Central seed column Mi15 17
Central seed column Mi4 18
Central seed column ML1 19
Central seed column Dm2 21
Central seed column Dm11 22
Central seed column L3 23
Central seed column Mi1 24

Central seed column R8 25
Central seed column Tm5a 26
Central seed column Tm5b 27
Central seed column Tm 28
Central seed column Tm5b-like 31
Central seed column Mi9 32
Central seed column L1 33
Central seed column aMe12 34
Central seed column Dm 35
Central seed column ML-VPN1 36
Central seed column C2 37
Central seed column Mt-VPN 38
Central seed column Mti 40
Central seed column Tm5a-like 41
Central seed column TmY10 41
Central seed column Mi10 42
Central seed column Mi 42
Central seed column C3 42
Central seed column Identified < 3 synapses 43
Central seed column Unidentified ≥ 3 synapses 47
Central seed column Unidentified < 3 synapses 48
DRA seed column Dm-DRA1 58

DRA seed column Dm9 61
DRA seed column MeTu-DRA 62
DRA seed column R7-DRA 72
DRA seed column Dm-DRA2 73
DRA seed column Dm2 75
DRA seed column R8-DRA 76
DRA seed column Mi15 77
DRA seed column Mti-DRA-1 78
DRA seed column MeMe-DRA 80
DRA seed column L3 81
DRA seed column VPN-DRA 82
DRA seed column L1 84
DRA seed column Tm20 85
DRA seed column Mti-DRA-2 86
DRA seed column Mi1 88
DRA seed column MeTu 89
DRA seed column Tm5-like 90
DRA seed column Mi9 90
DRA seed column Dm11 91
DRA seed column aMe12 91
DRA seed column TmY 92
DRA seed column ML-VPN2 92

DRA seed column C2 93
DRA seed column Identified < 3 synapses. 94
DRA seed column Unidentified ≥ 3 synapses. 100
DRA seed column Unidentified < 3 synapses. 101

Central seed column Dm9 (6 cells)

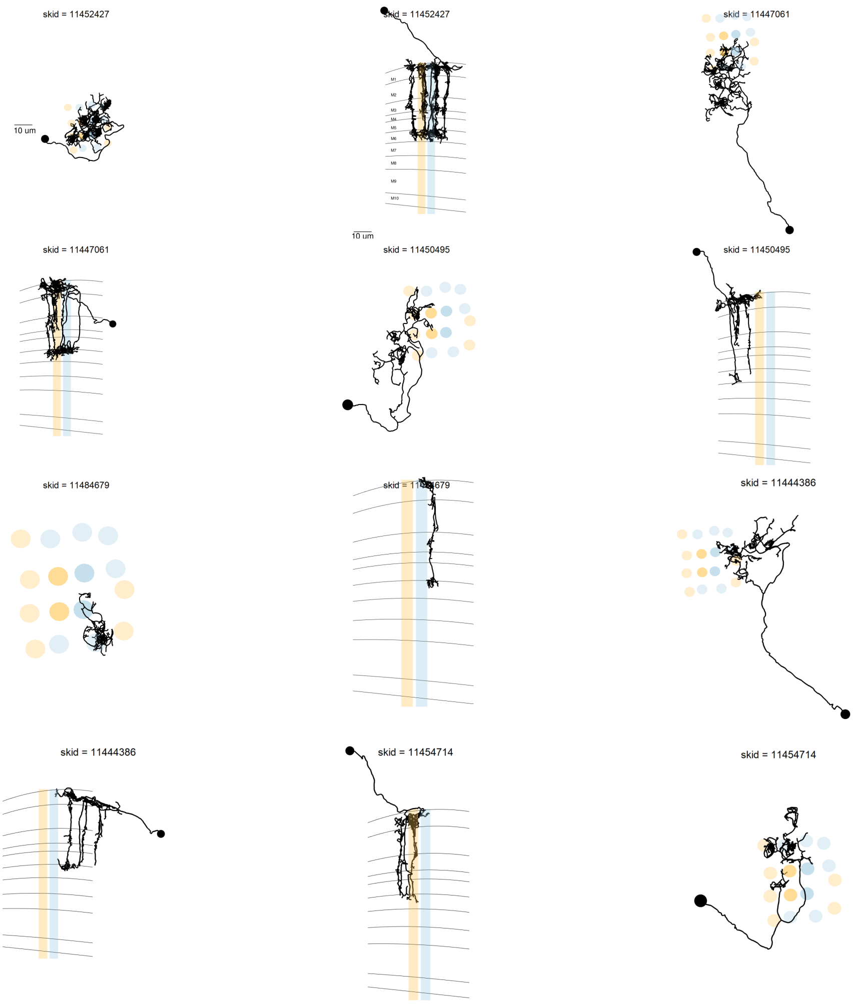

Central seed column Dm8 (15 cells)

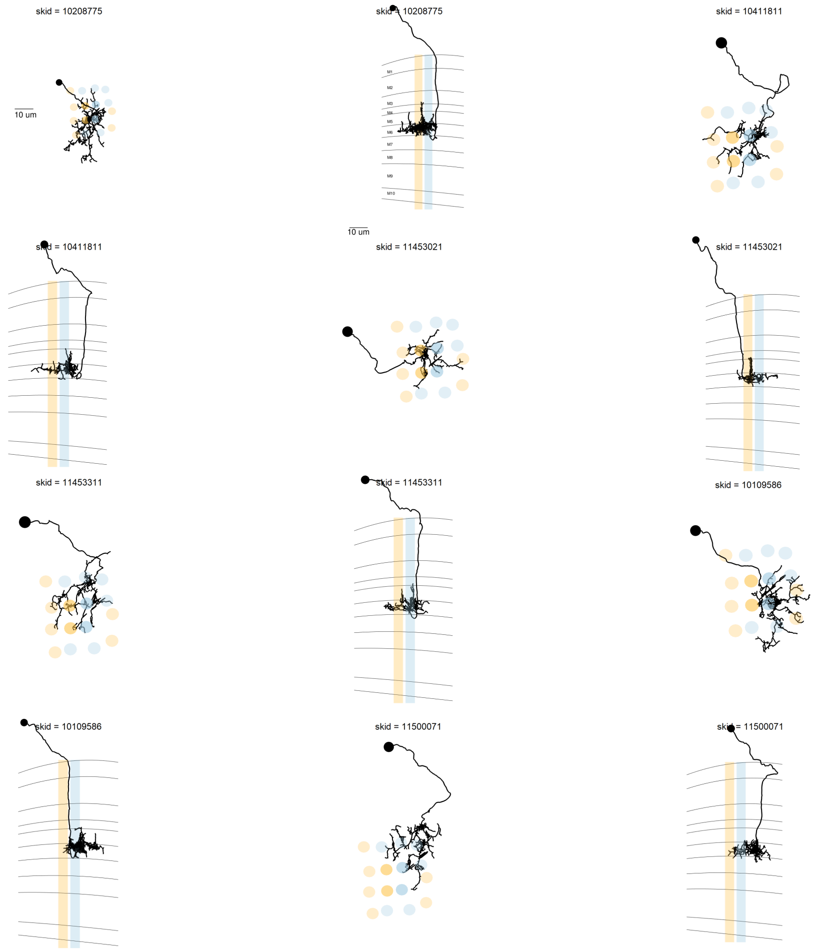

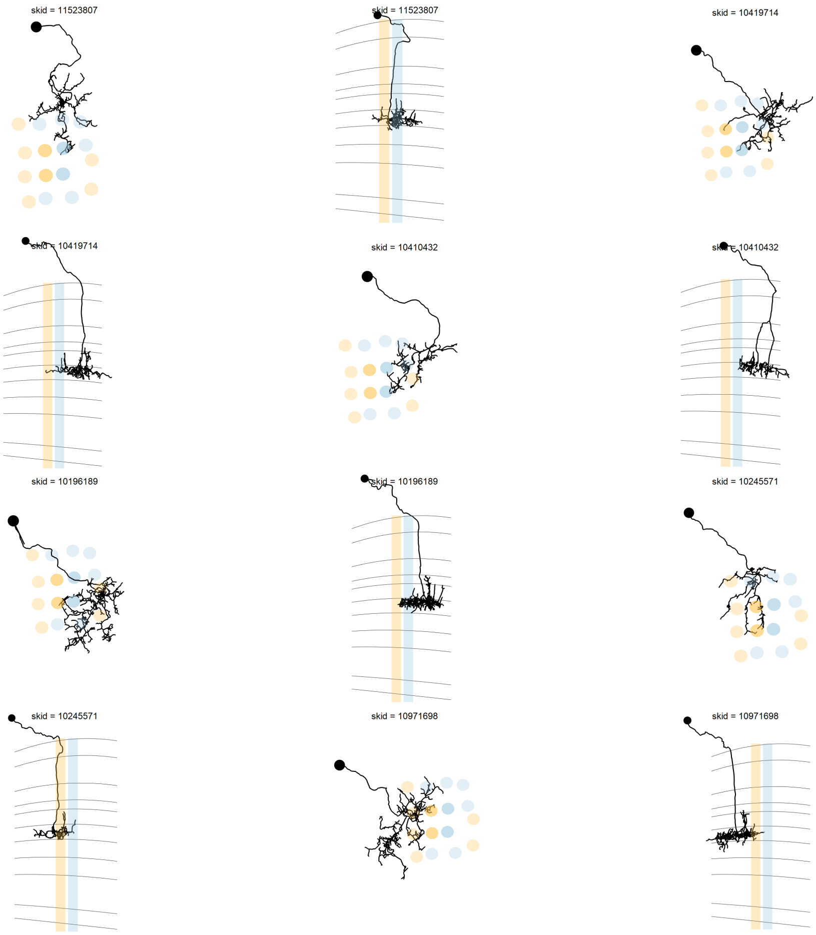
skid = 11523807
skid = 11523807
skid = 10419714
skid = 10419714
skid = 10410432
skid = 10410432
skid = 10196189
skid = 10196189
skid = 10245571
skid = 10245571
skid = 10971698
skid = 10971698

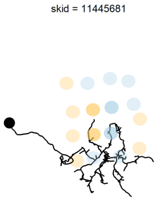
skid = 11445681
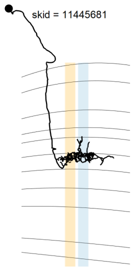
skid = 11445681
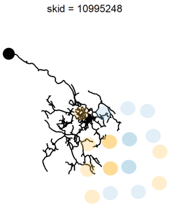
skid = 10995248
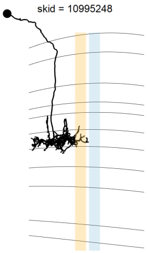
skid = 10995248

Central seed column MeTu (7 cells)

skid = 10409863

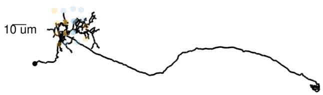

skid = 10409863

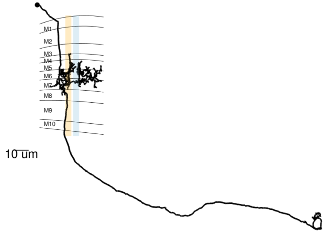

skid = 11455122

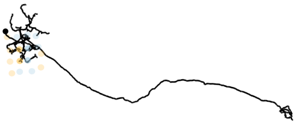

skid = 11455122

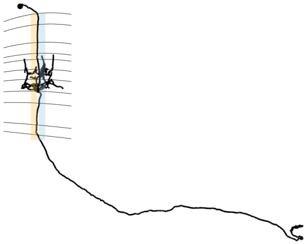

skid = 11455156

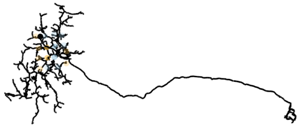

skid = 11455156

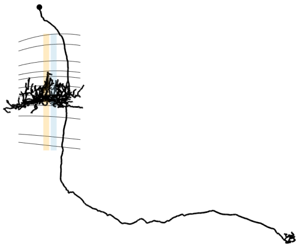

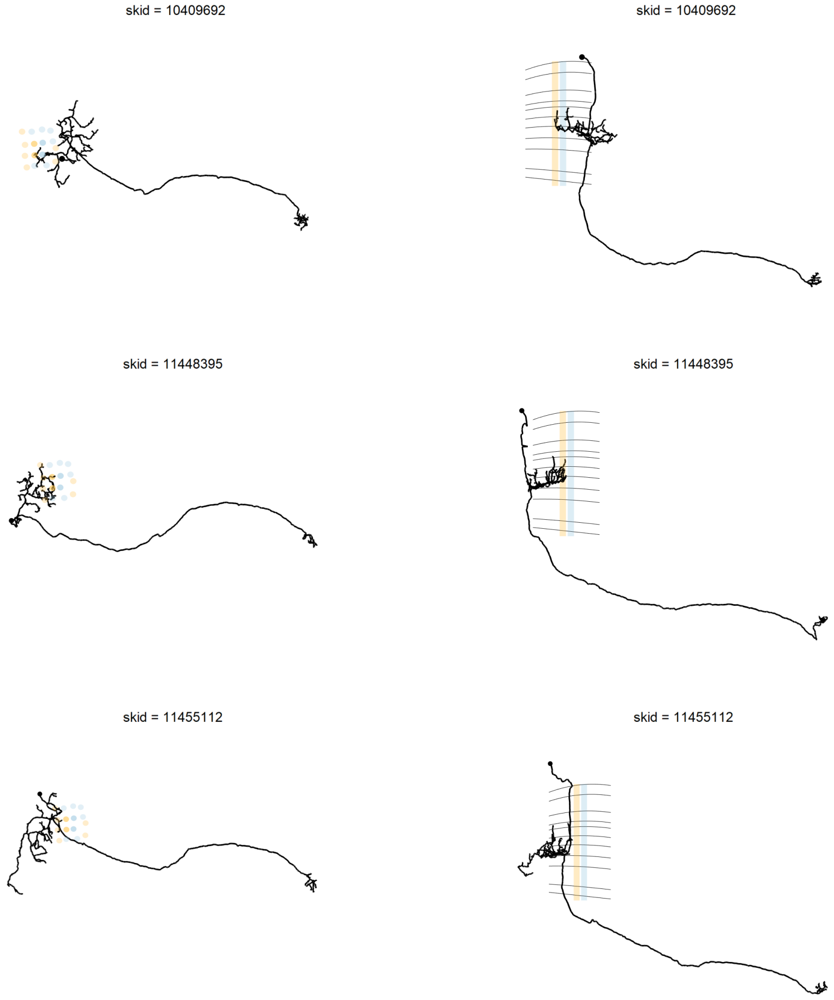
skid = 10409692
skid = 10409692
skid = 11448395
skid = 11448395
skid = 11455112
skid = 11455112

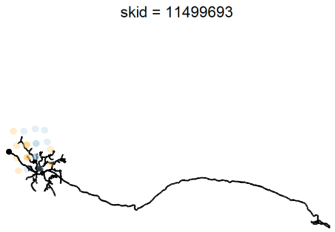
skid = 11499693

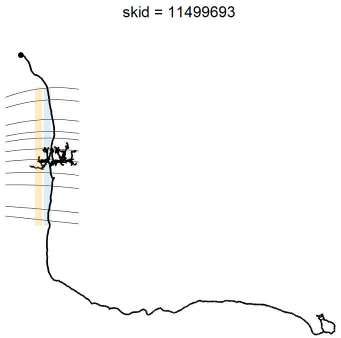
skid = 11499693

Central seed column R7 (5 cells)

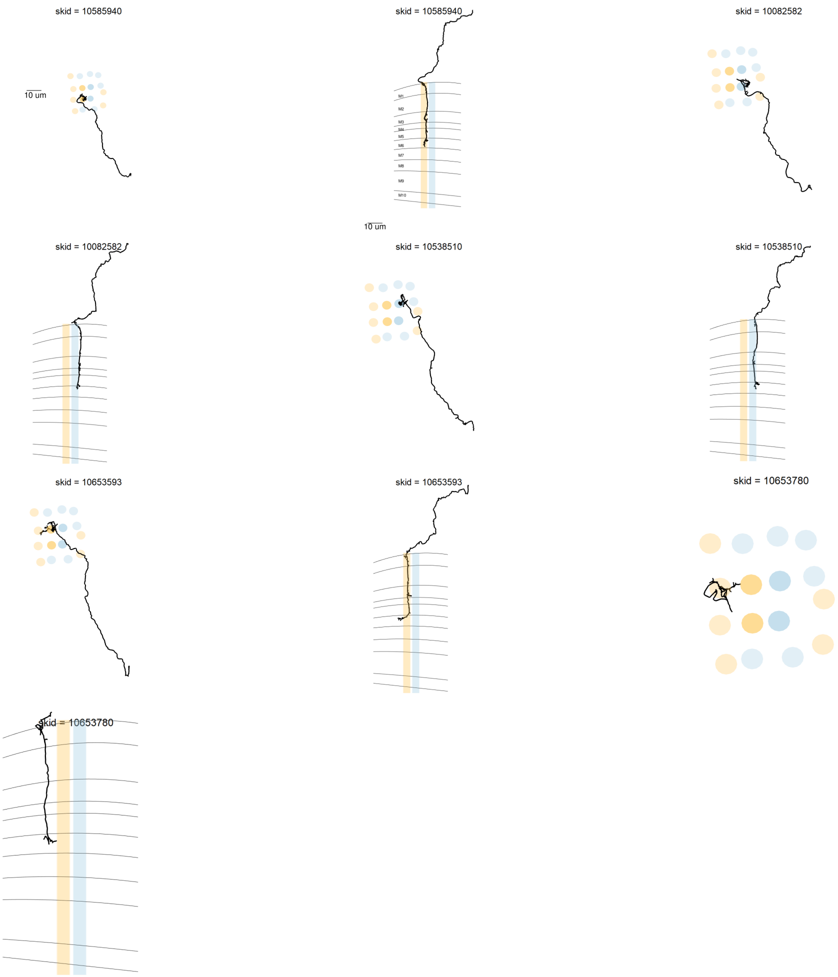

Central seed column Tm5c (6 cells)

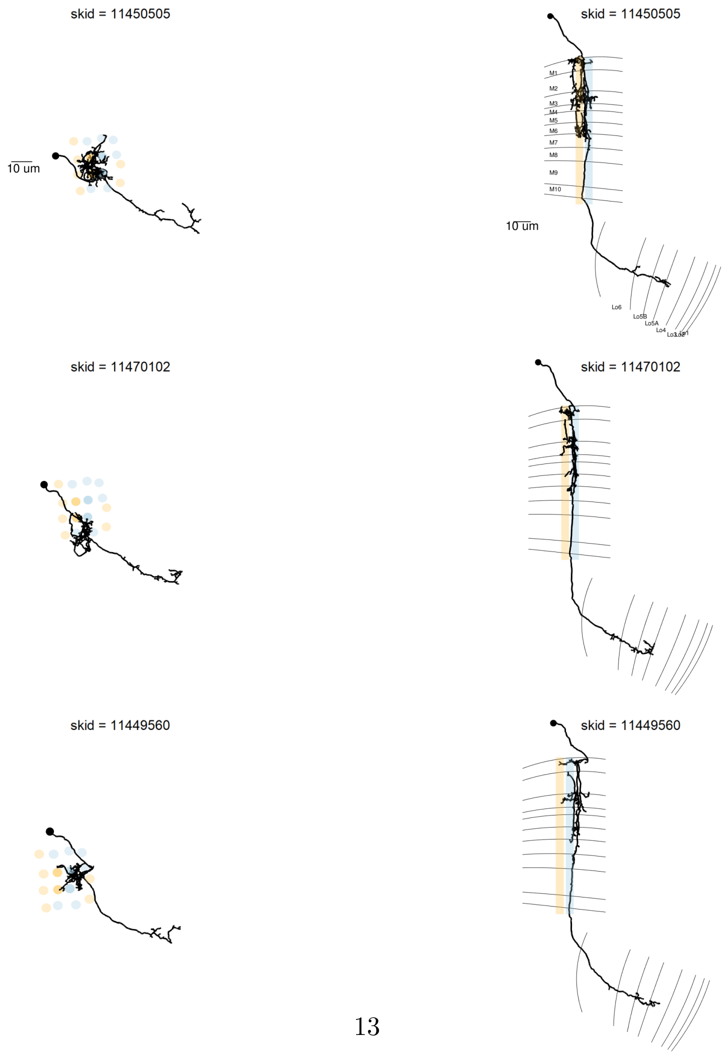

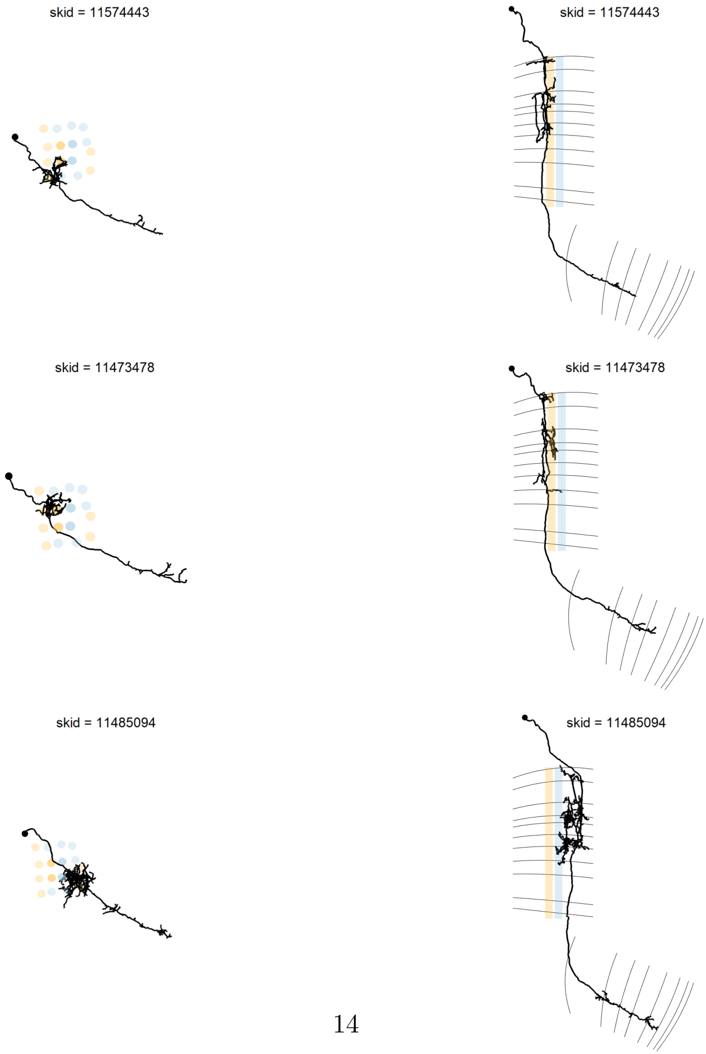
skid = 11574443
skid = 11574443
skid = 11473478
skid = 11473478
skid = 11485094
skid = 11485094

Central seed column Tm20 (4 cells)

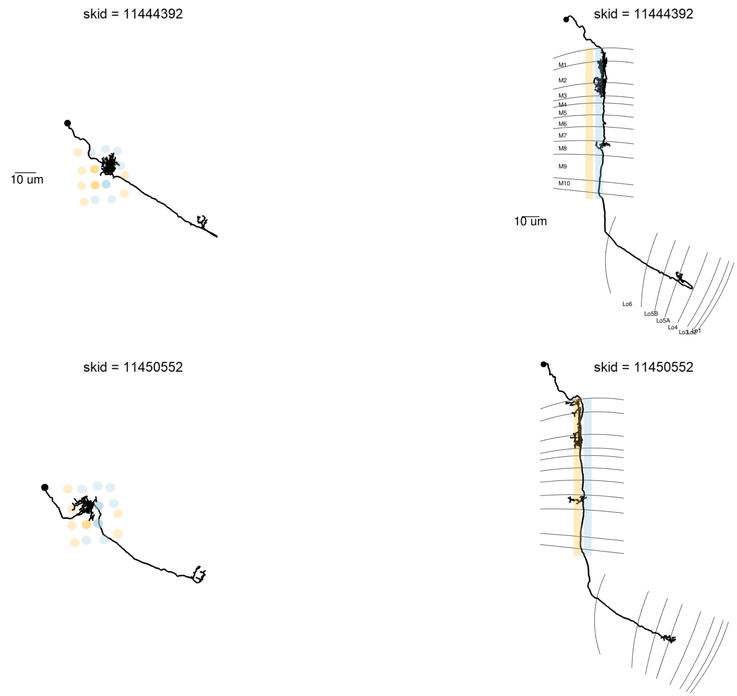

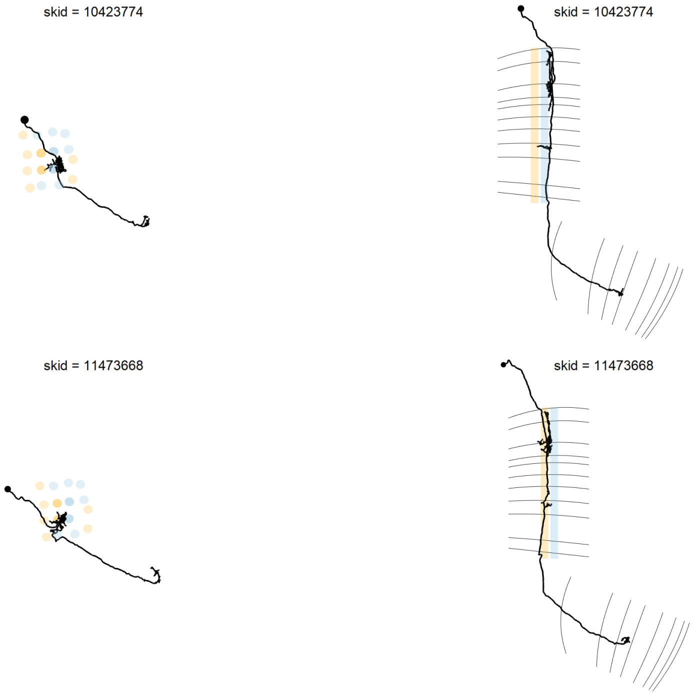
skid = 10423774
skid = 10423774
skid = 11473668
skid = 11473668

Central seed column Mi15 (4 cells)

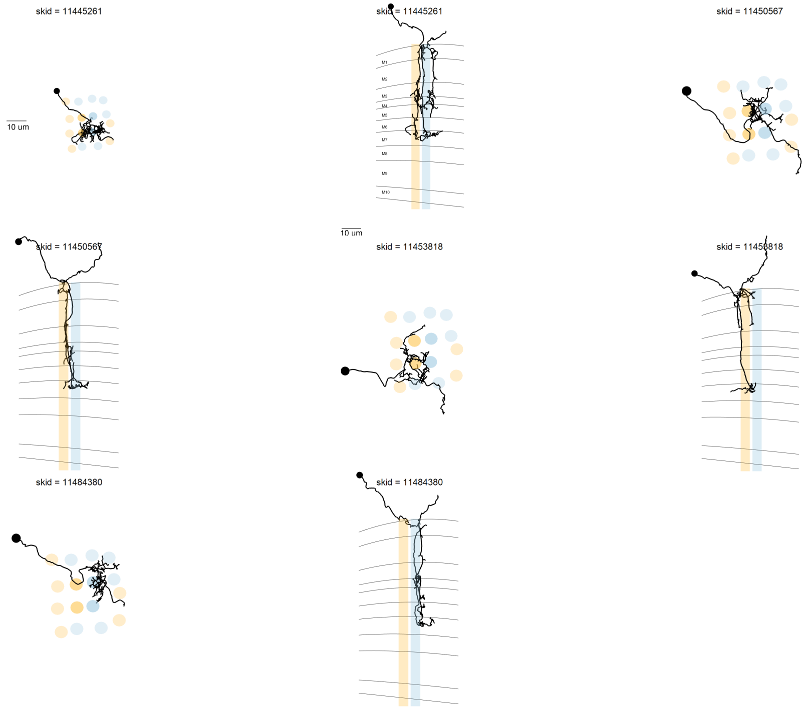

Central seed column Mi4 (4 cells)

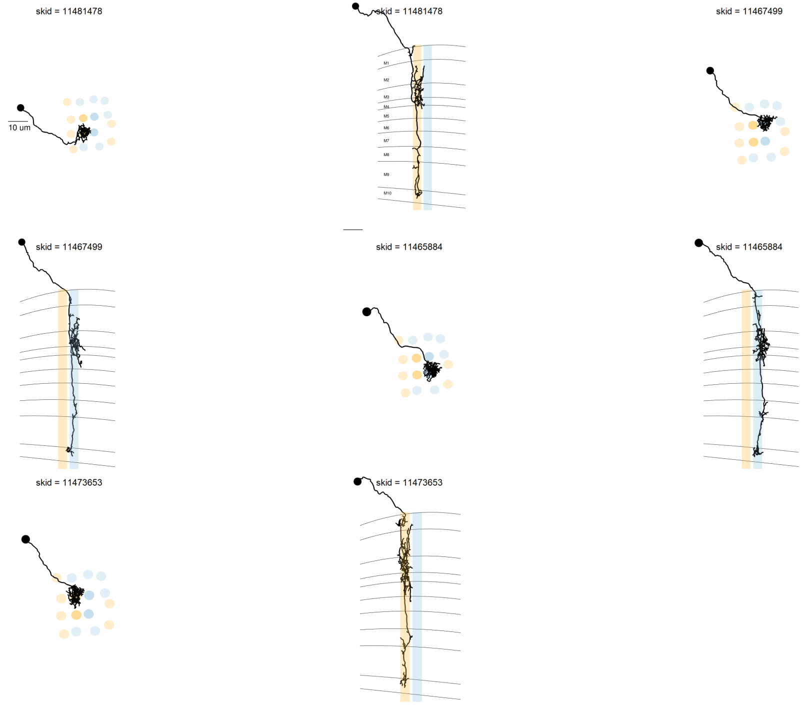

Central seed column ML1 (4 cells)

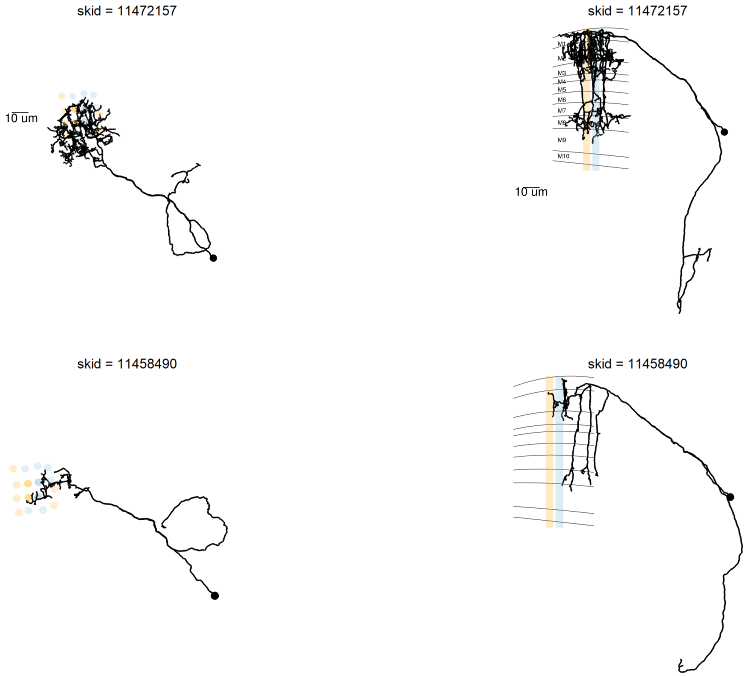

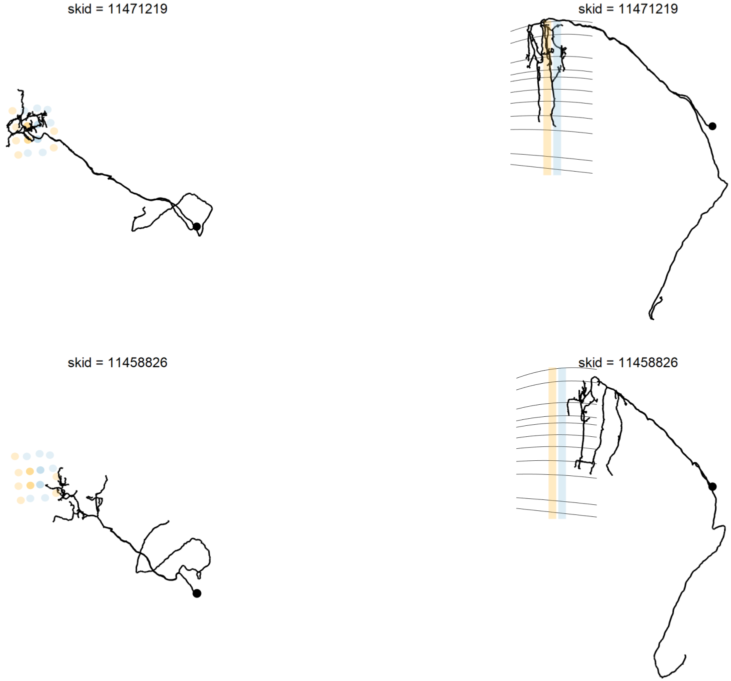
skid = 11471219
skid = 11471219
skid = 11458826
skid = 11458826

Central seed column Dm2 (4 cells)

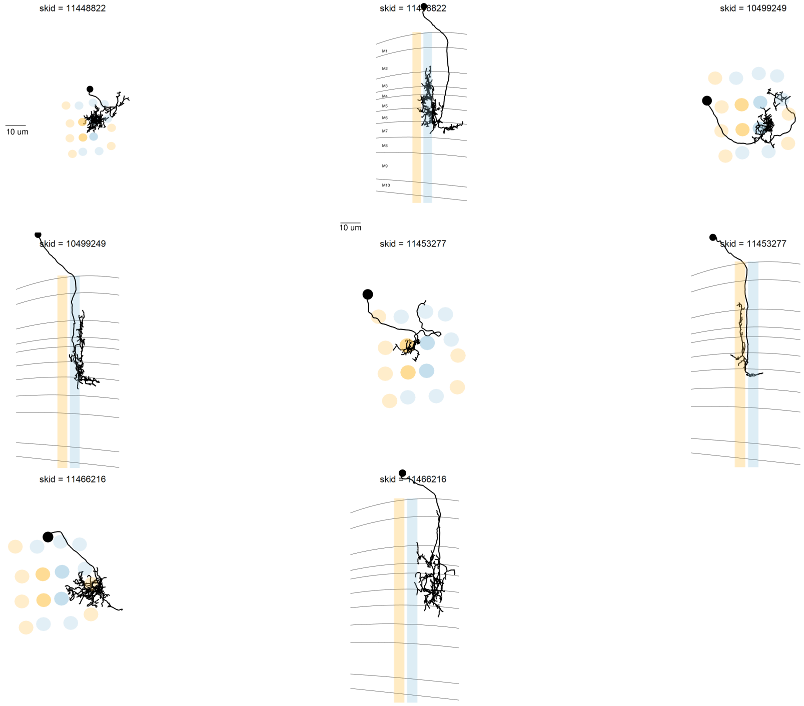

Central seed column Dm11 (2 cells)

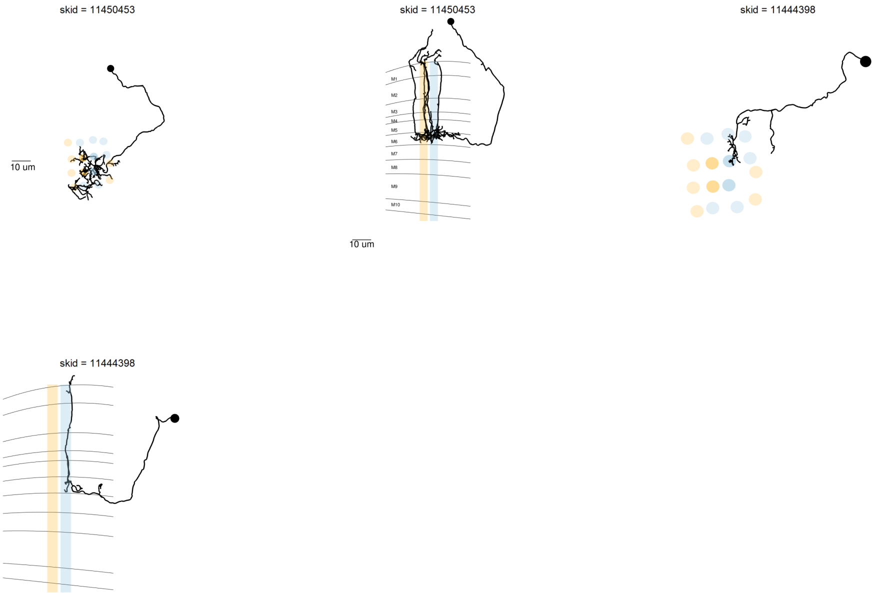

Central seed column L3 (4 cells)

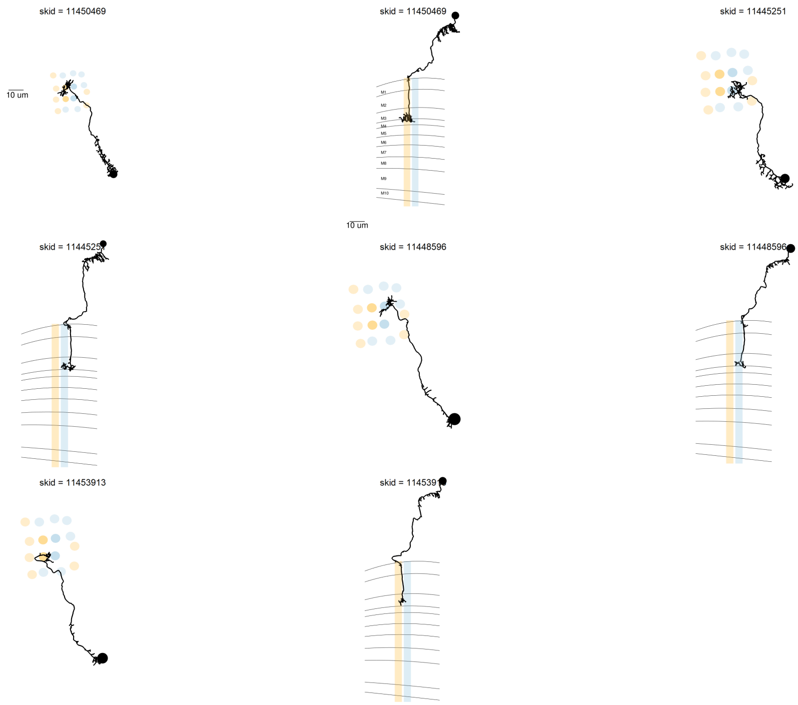

Central seed column Mi1 (4 cells)

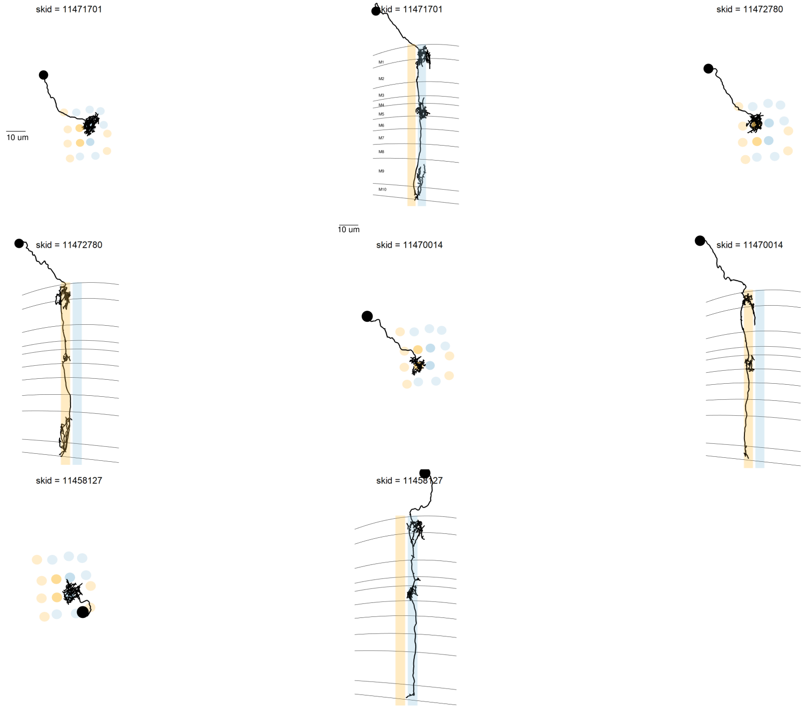

Central seed column R8 (4 cells)

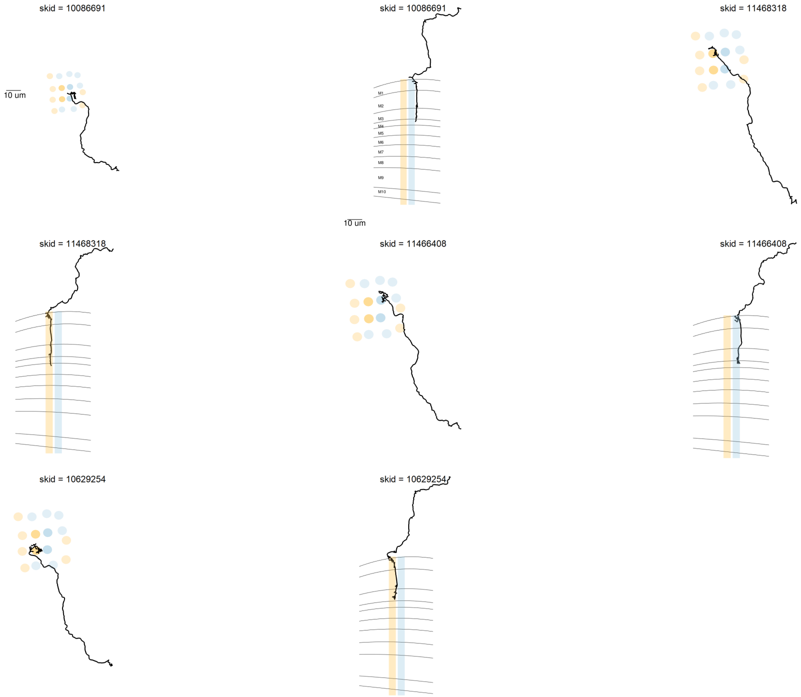

Central seed column Tm5a (2 cells)

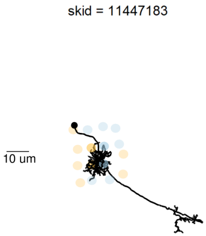

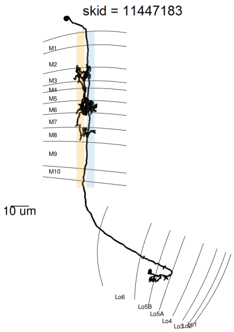

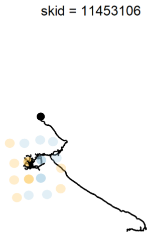

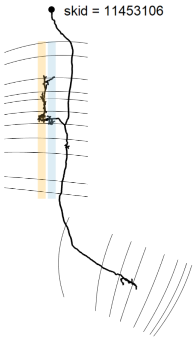

Central seed column Tm5b (2 cells)

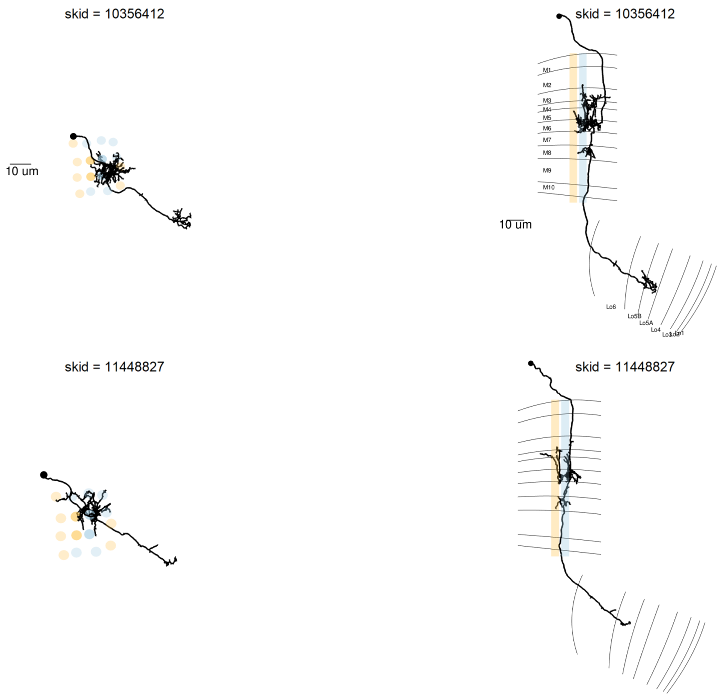

Central seed column Tm (9 cells)

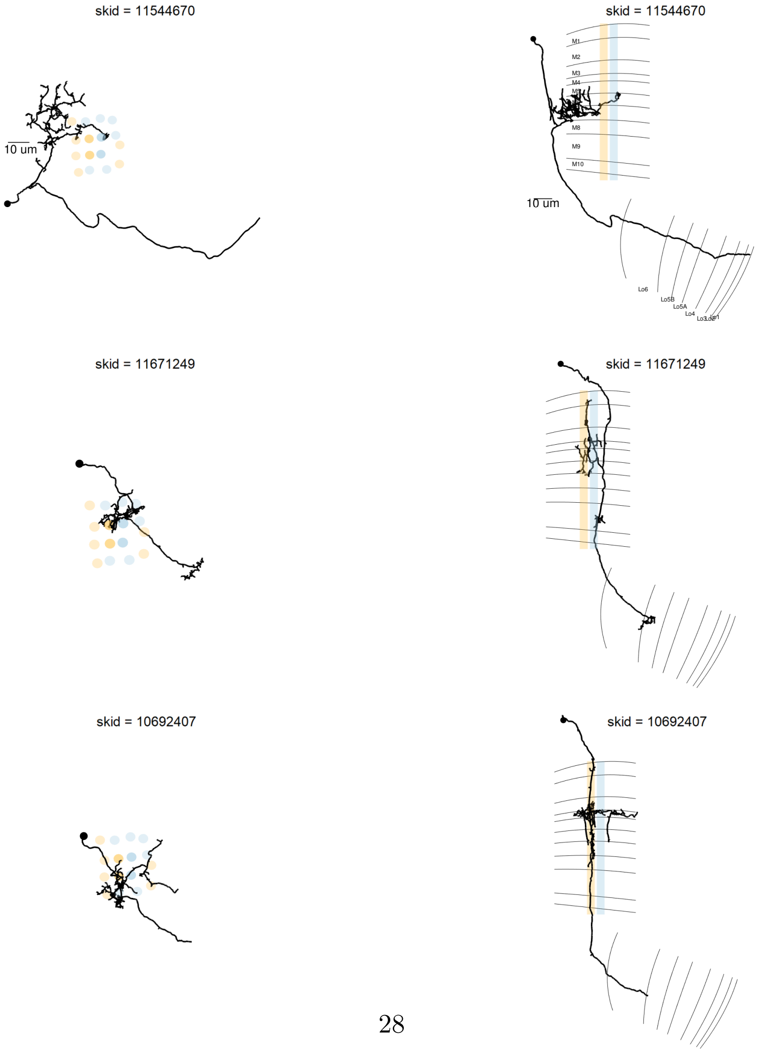

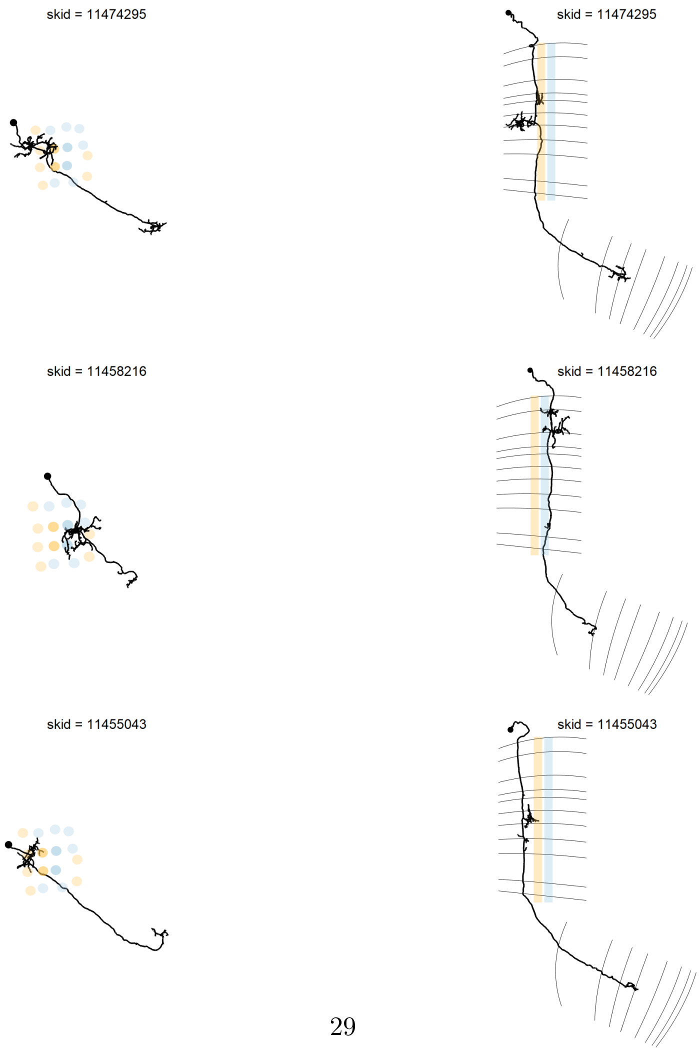
skid = 11474295
skid = 11474295
skid = 11458216
skid = 11458216
skid = 11455043
skid = 11455043

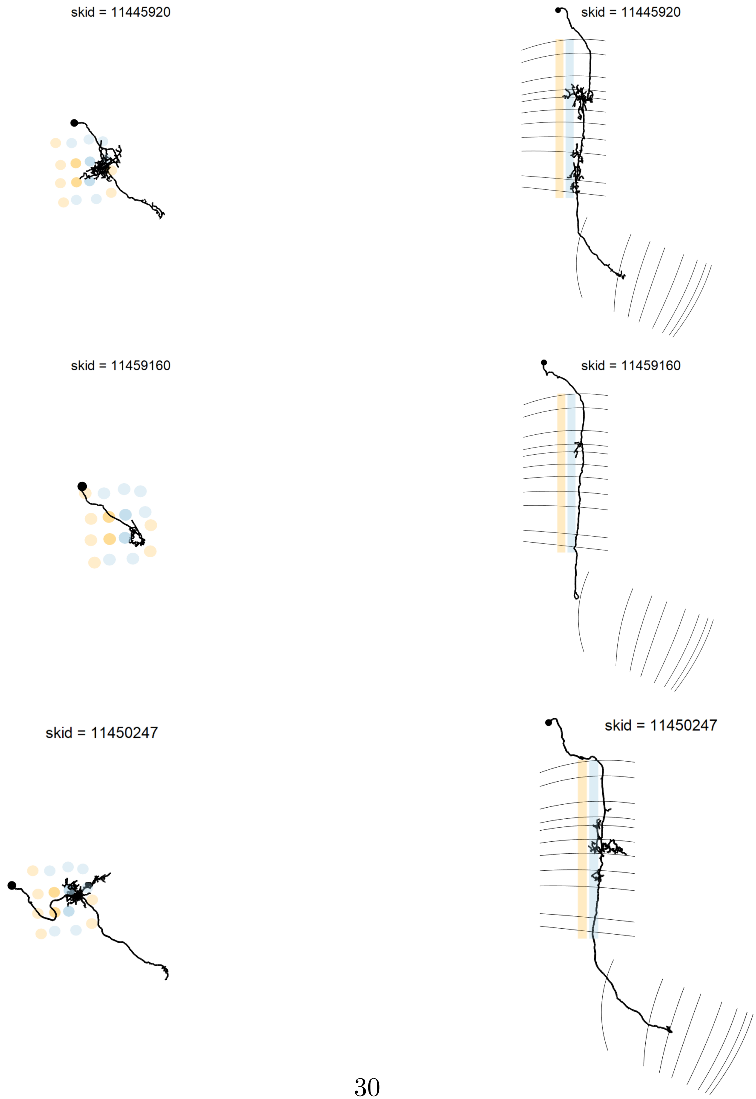
skid = 11445920
skid = 11445920
skid = 11459160
skid = 11459160
skid = 11450247
skid = 11450247

Central seed column Tm5b-like (3 cells)

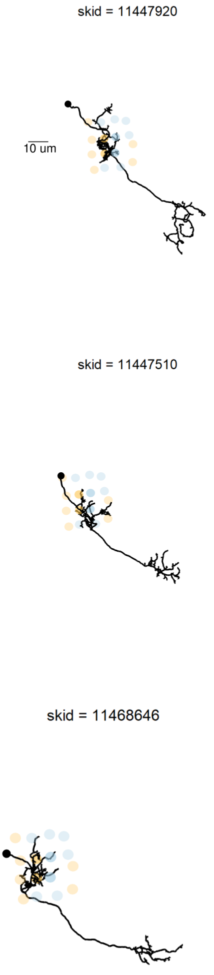

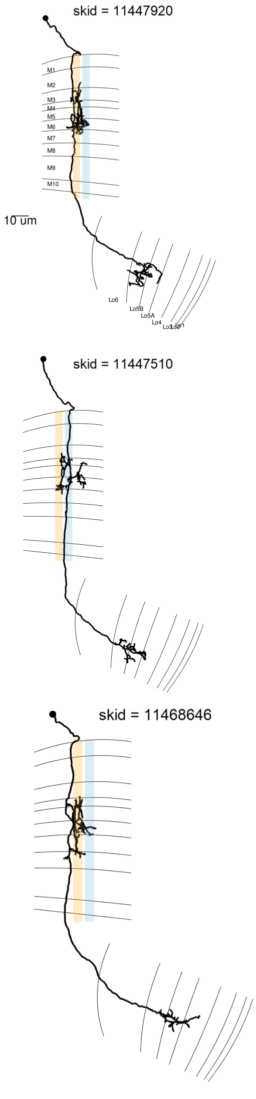

Central seed column Mi9 (4 cells)

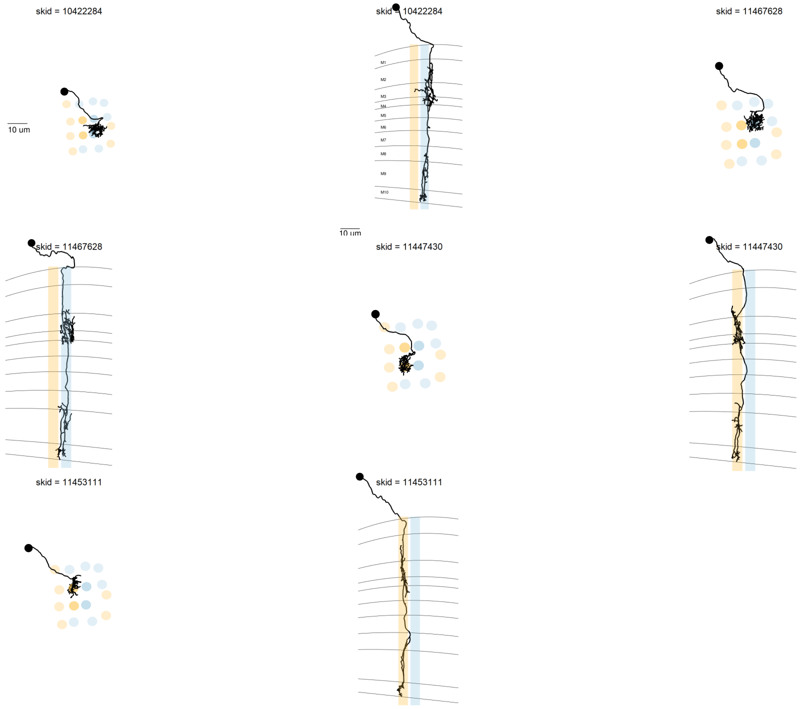

Central seed column L1 (4 cells)

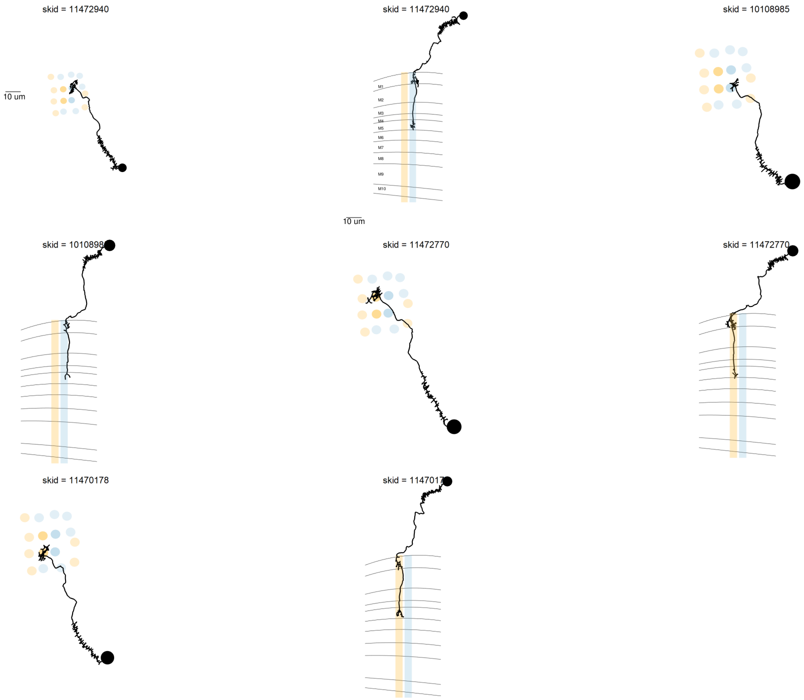

Central seed column aMe12 (3 cells)

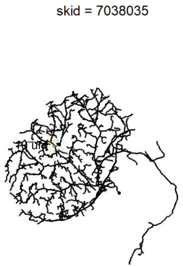

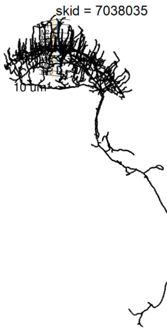

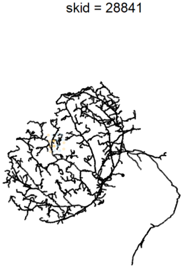

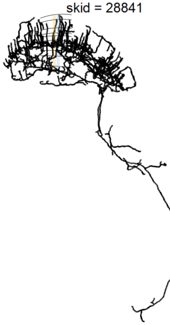

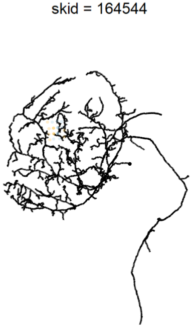

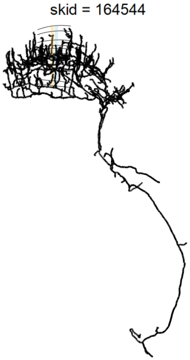

Central seed column Dm (5 cells)

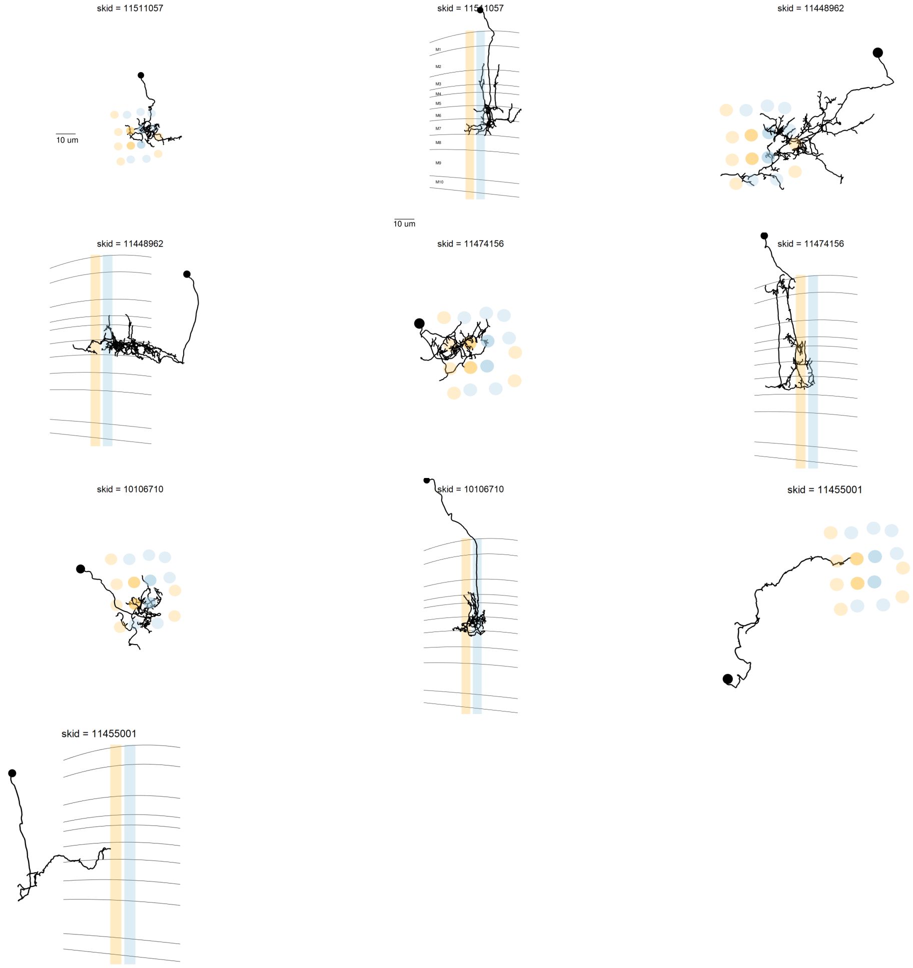

Central seed column ML-VPN1 (3 cells)

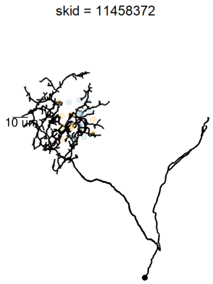

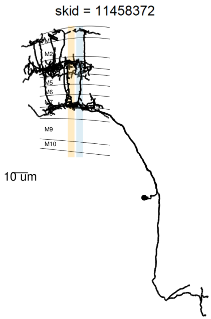

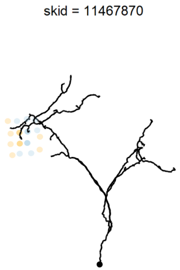

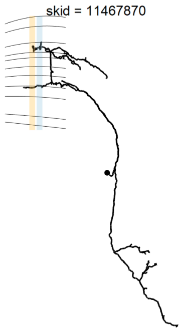

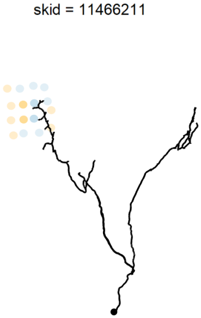

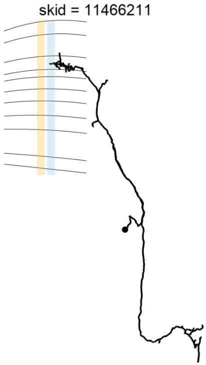

Central seed column C2 (2 cells)

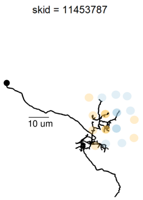

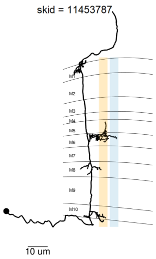

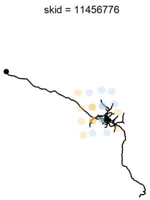

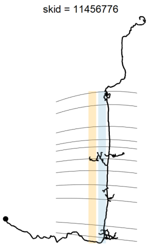

Central seed column Mt-VPN (4 cells)

skid = 11453464

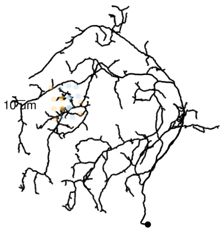

skid = 11453464

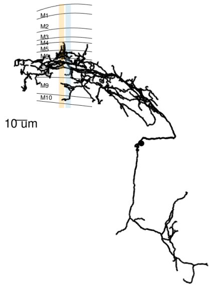

skid = 11469481

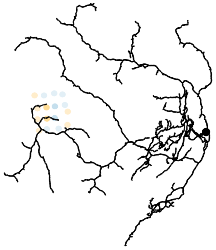

skid = 11469481

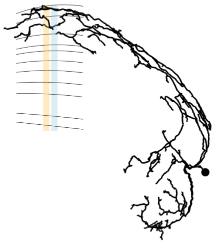

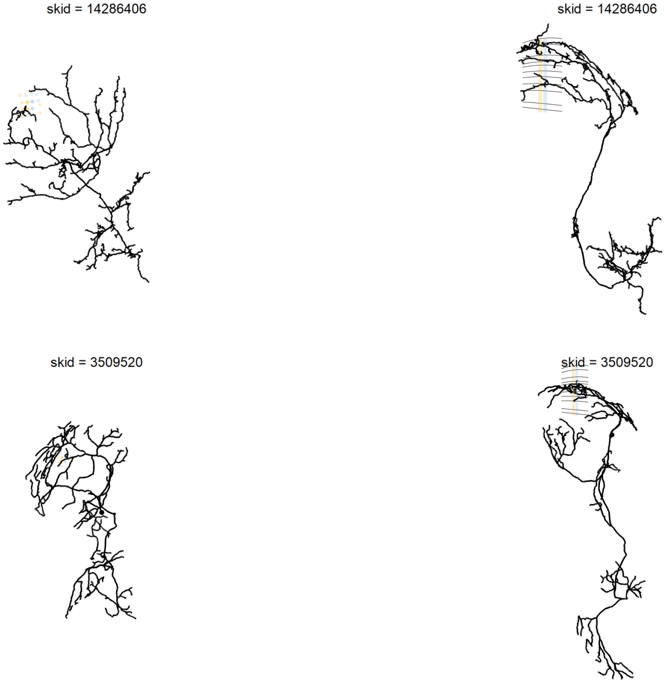
skid = 14286406
skid = 14286406
skid = 3509520
skid = 3509520

Central seed column Mti (3 cells)

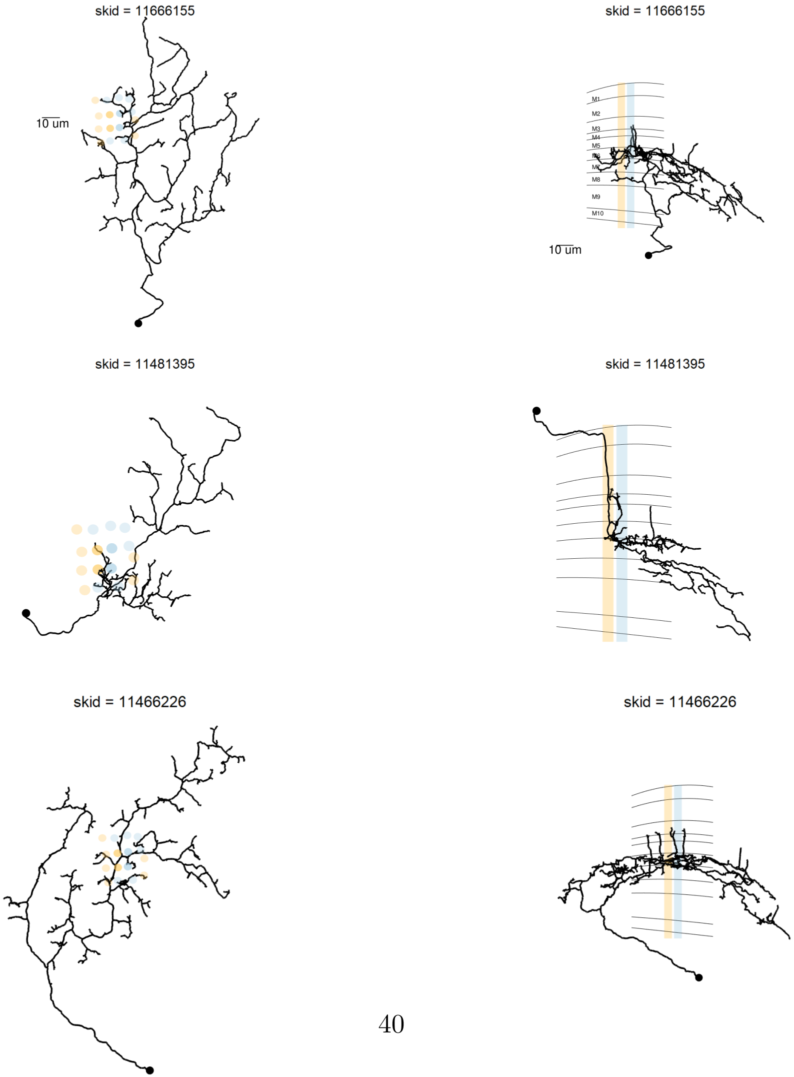

Central seed column Tm5a-like (1 cell)

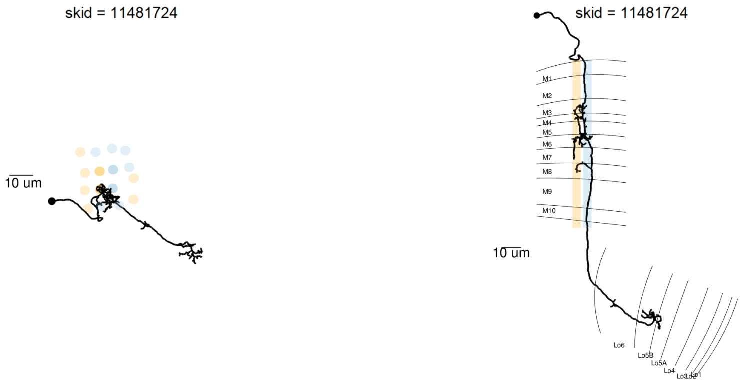

Central seed column TmY10 (1 cell)

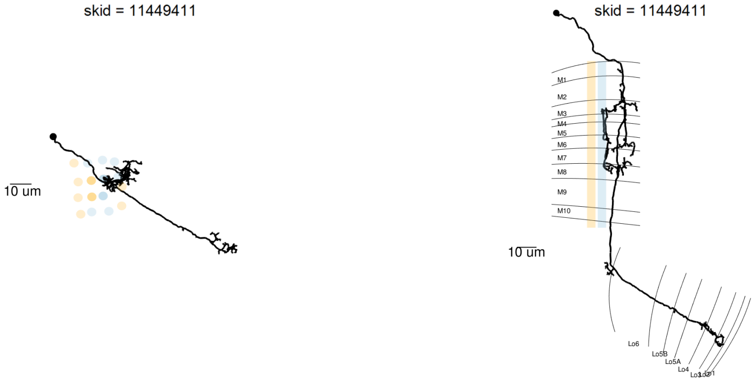

Central seed column Mi10 (1 cell)

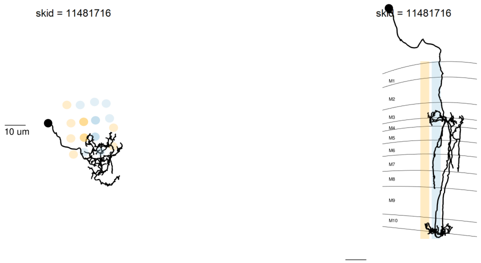

Central seed column Mi (1 cell)

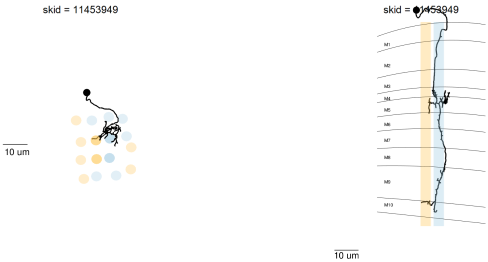

Central seed column C3 (1 cell)

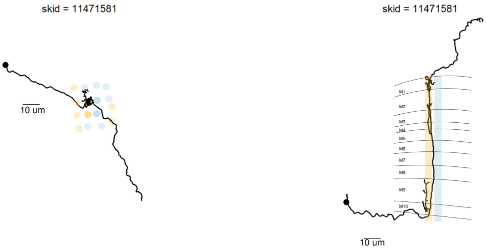

Central seed column Identified < 3 synapses (26 cells)

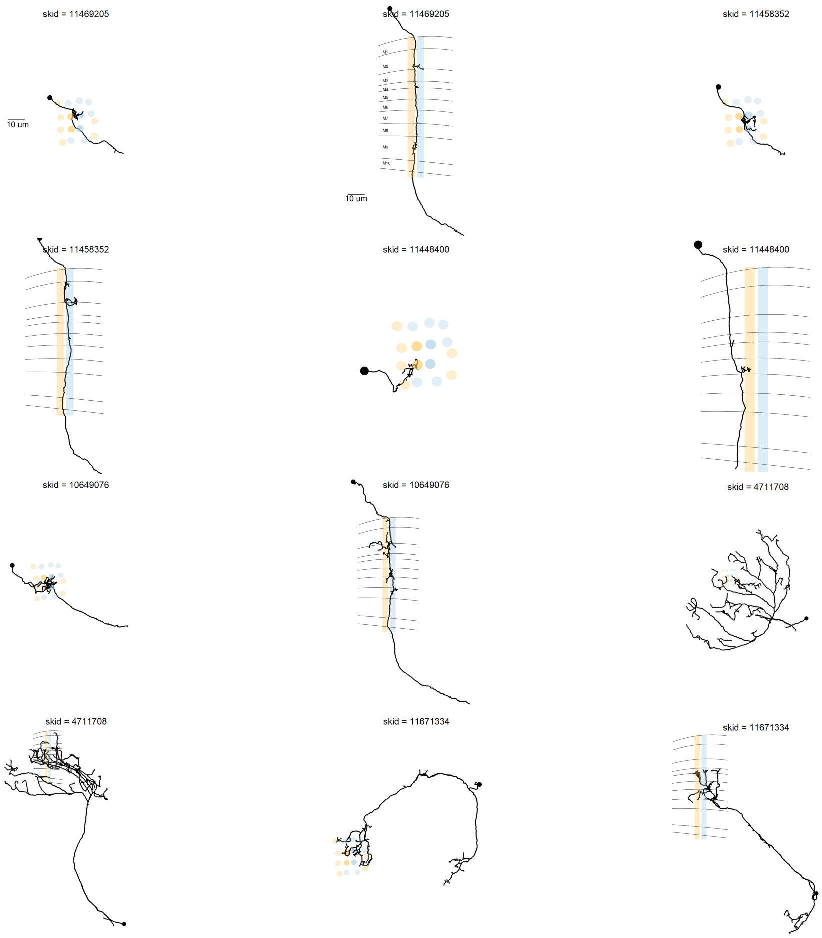

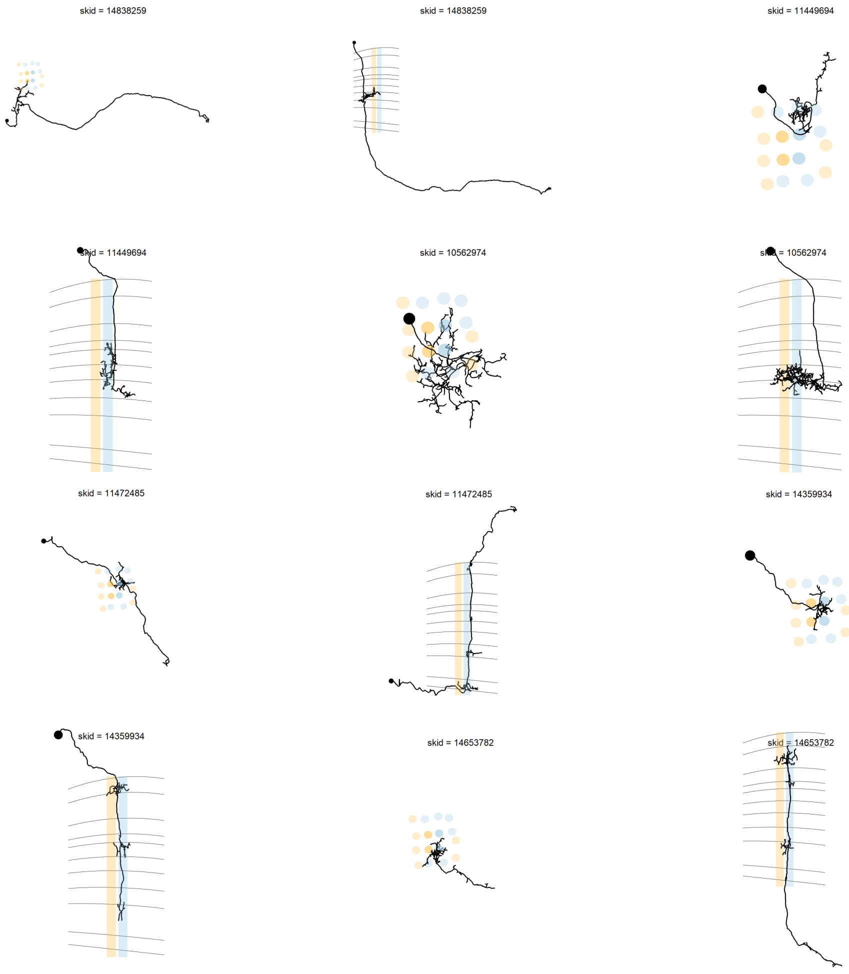
skid = 14838259
skid = 14838259
skid = 11449694
skid = 11449694
skid = 10562974
skid = 10562974
skid = 11472485
skid = 11472485
skid = 14359934
skid = 14359934
skid = 14653782
skid = 14653782

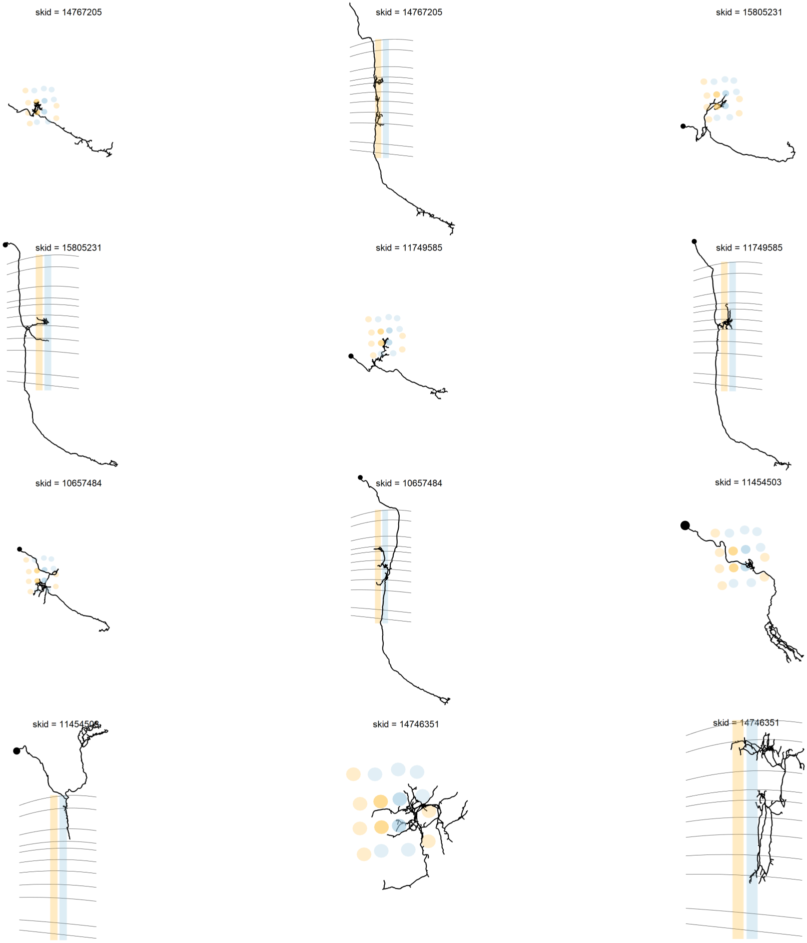
skid = 14767205
skid = 14767205
skid = 15805231
skid = 15805231
skid = 11749585
skid = 11749585
skid = 10657484
skid = 10657484
skid = 11454503
skid = 11454503
skid = 14746351
skid = 14746351

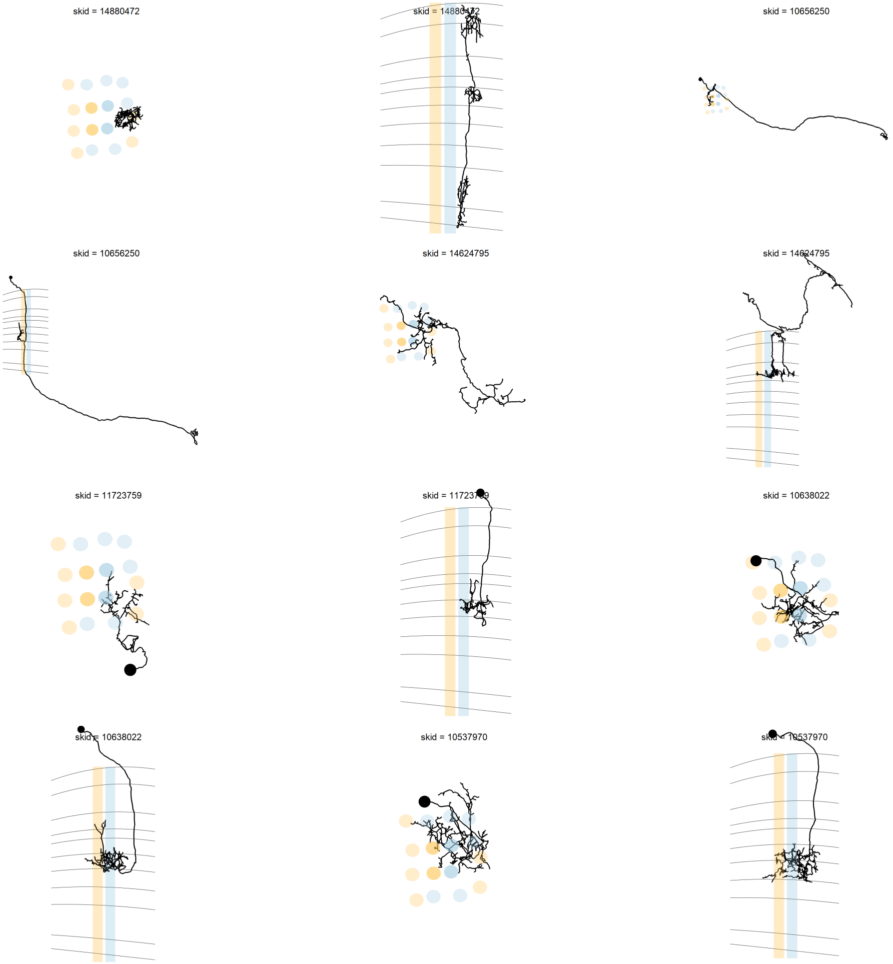
skid = 14880472
skid = 10656250
skid = 10656250
skid = 14624795
skid = 11723759
skid = 10638022
skid = 10537970

Central seed column Unidentified ≥ 3 synapses (2 cells)

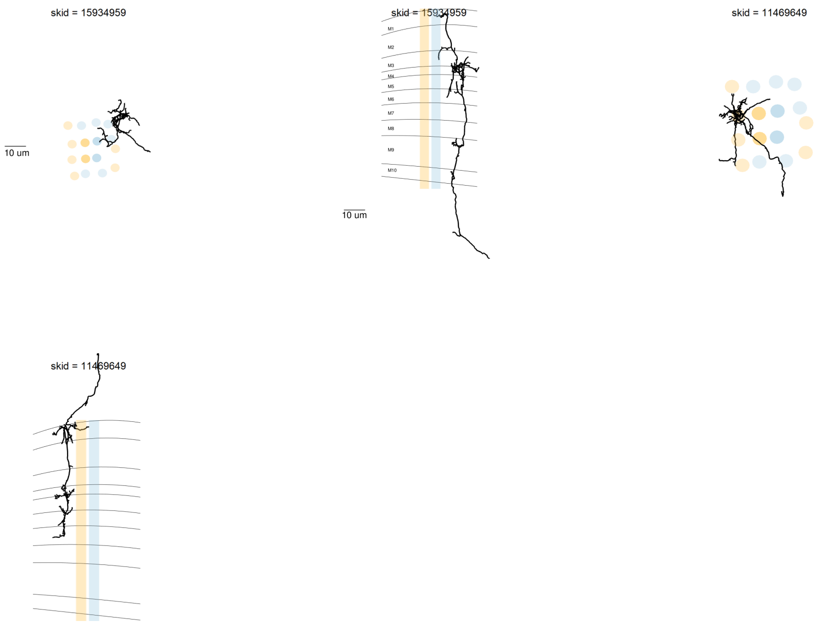

Central seed column Unidentified < 3 synapses (62 cells)

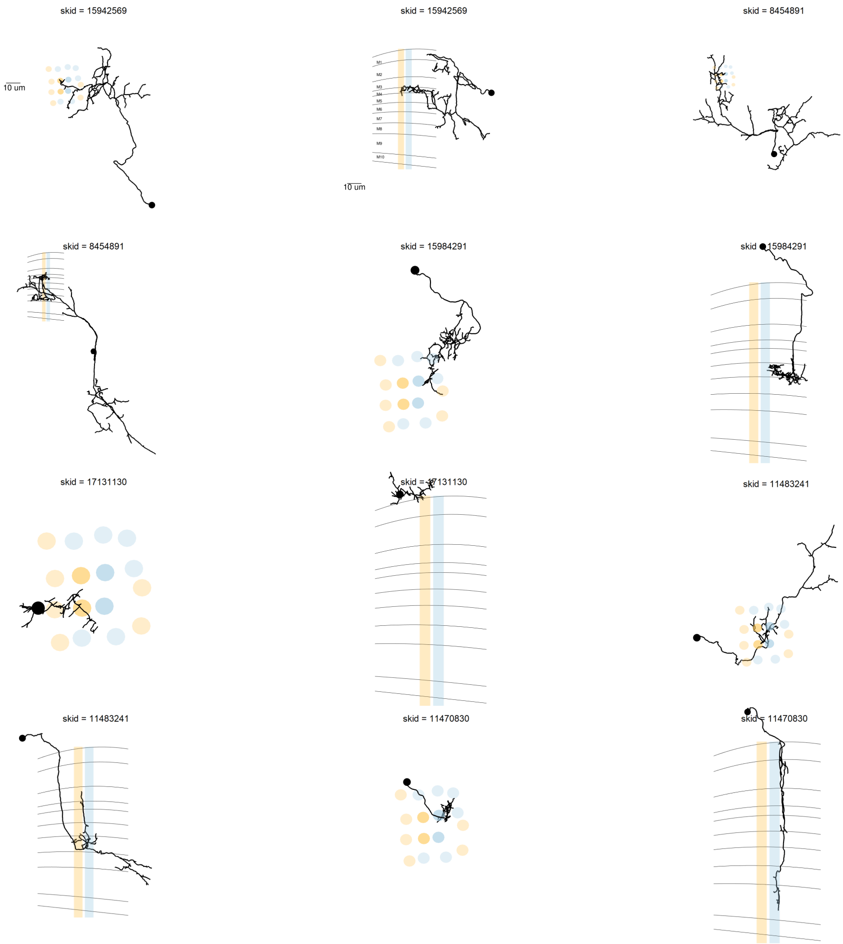

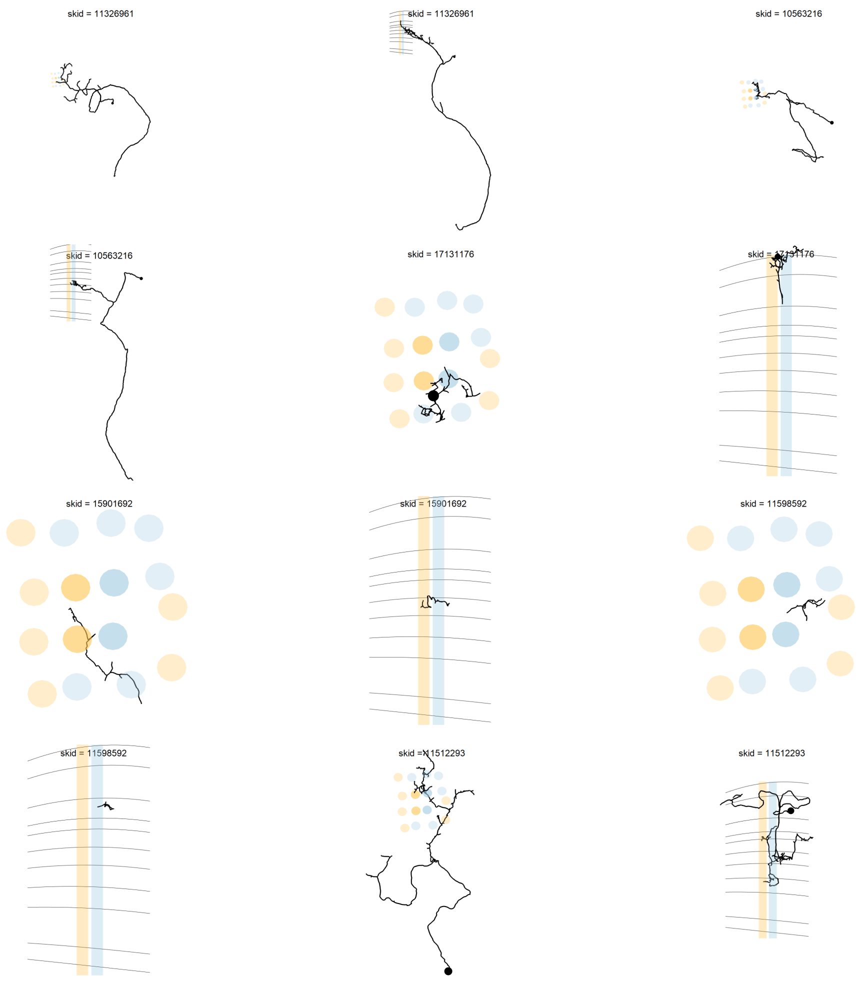
skid = 11326961
skid = 11326961
skid = 10563216
skid = 10563216
skid = 17131176
skid = 15901692
skid = 15901692
skid = 11598592
skid = 11598592
skid = 11512293

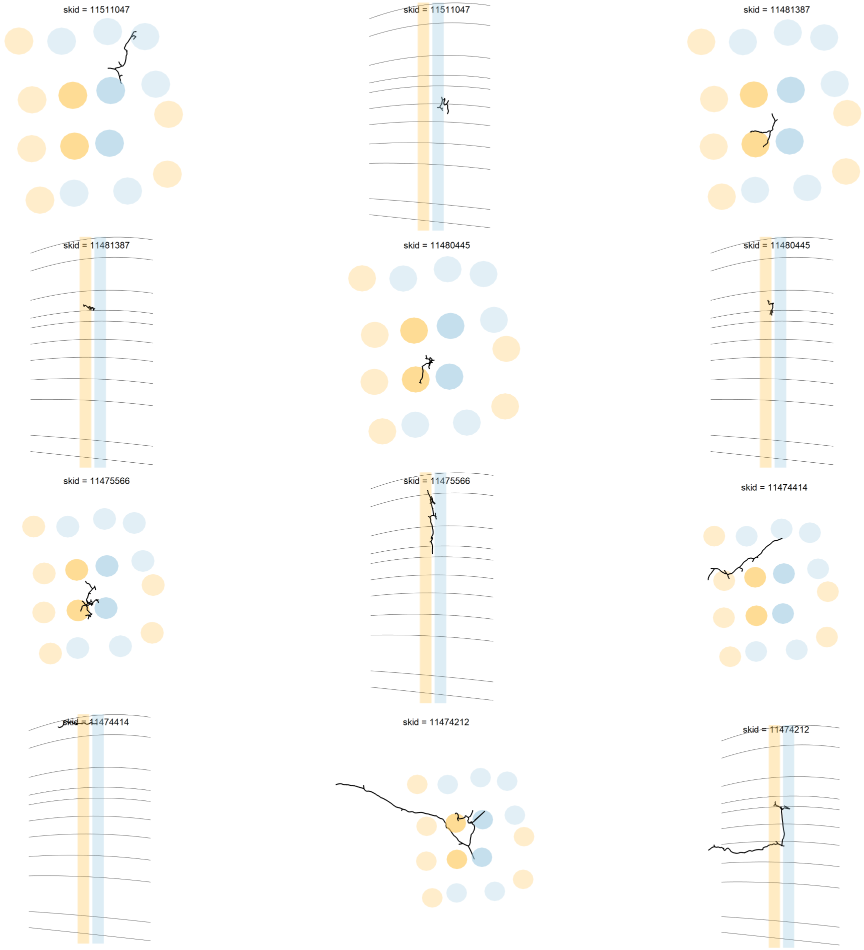
skid = 11511047
skid = 11511047
skid = 11481387
skid = 11481387
skid = 11480445
skid = 11480445
skid = 11475566
skid = 11475566
skid = 11474414
skid = 11474414
skid = 11474212
skid = 11474212

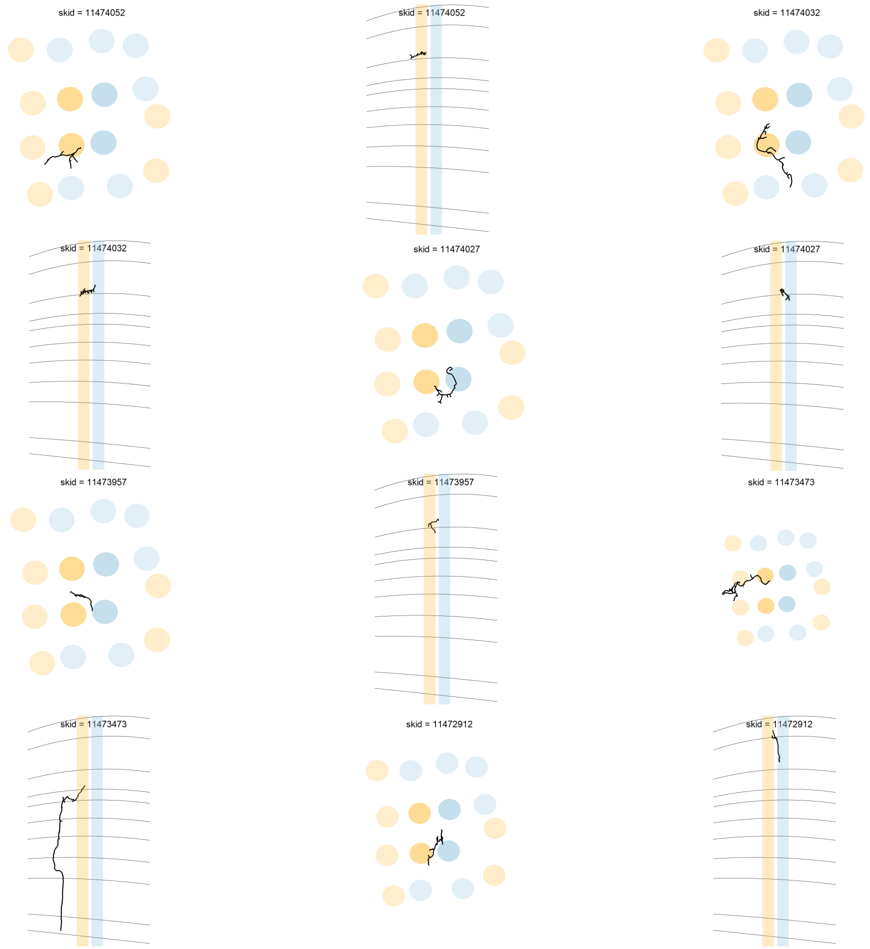
skid = 11474052
skid = 11474052
skid = 11474032
skid = 11474032
skid = 11474027
skid = 11474027
skid = 11473957
skid = 11473957
skid = 11473473
skid = 11473473
skid = 11472912
skid = 11472912

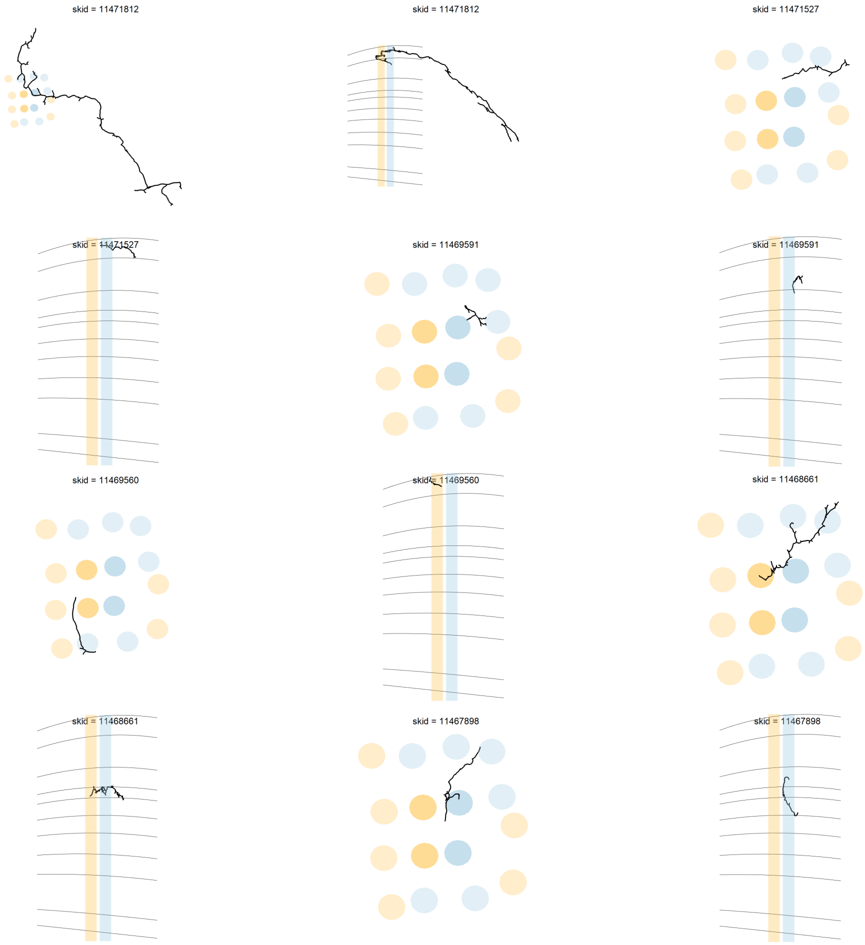
skid = 11471812
skid = 11471812
skid = 11471527
skid = 11471527
skid = 11469591
skid = 11469591
skid = 11469560
skid = 11469560
skid = 11468661
skid = 11468661
skid = 11467898
skid = 11467898

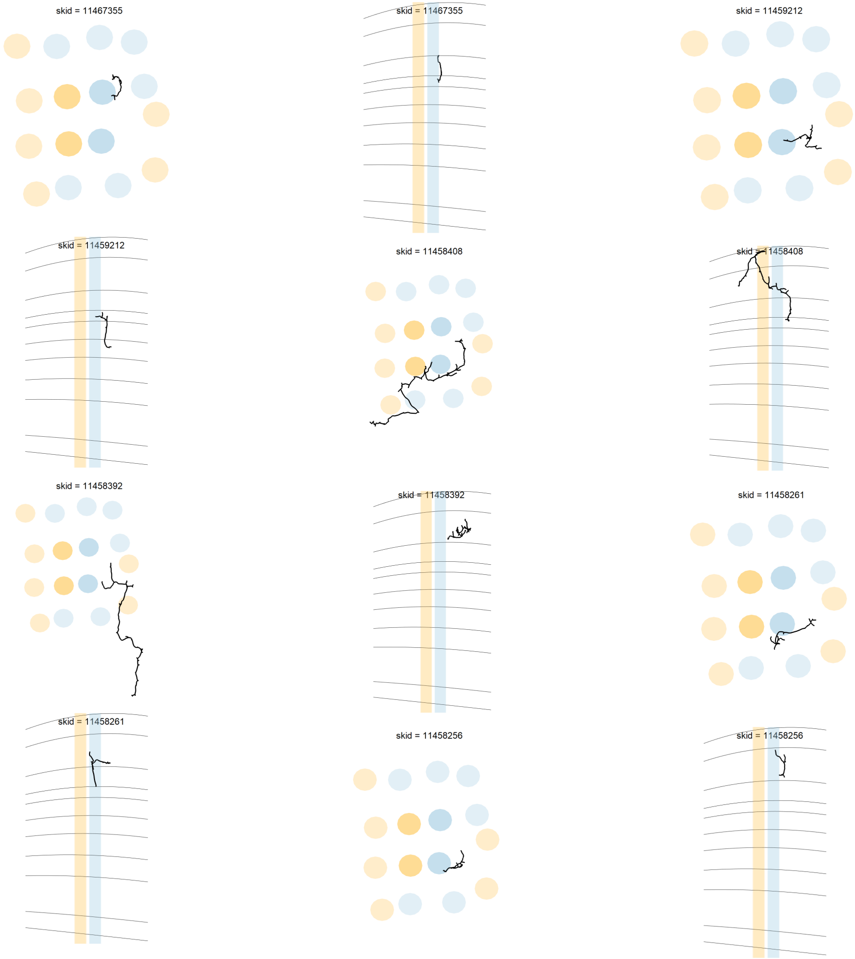
skid = 11467355
skid = 11467355
skid = 11459212
skid = 11459212
skid = 11458408
skid = 11458408
skid = 11458392
skid = 11458392
skid = 11458261
skid = 11458261
skid = 11458256
skid = 11458256

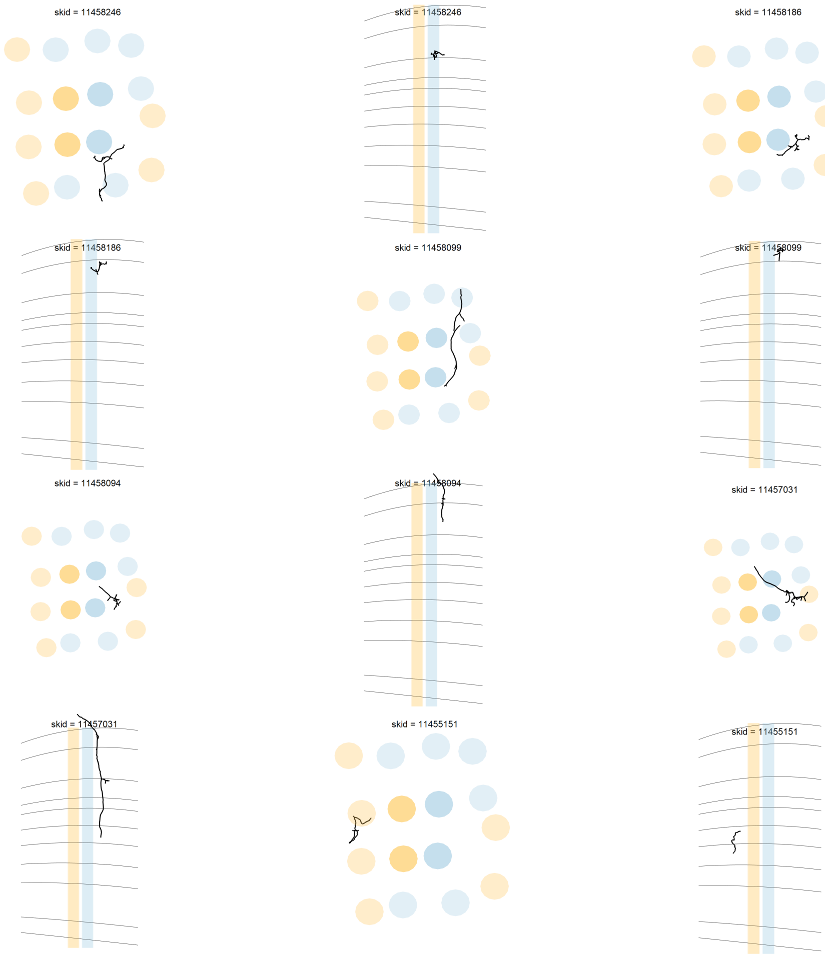
skid = 11458246
skid = 11458246
skid = 11458186
skid = 11458186
skid = 11458099
skid = 11458099
skid = 11458094
skid = 11458094
skid = 11457031
skid = 11457031
skid = 11455151
skid = 11455151

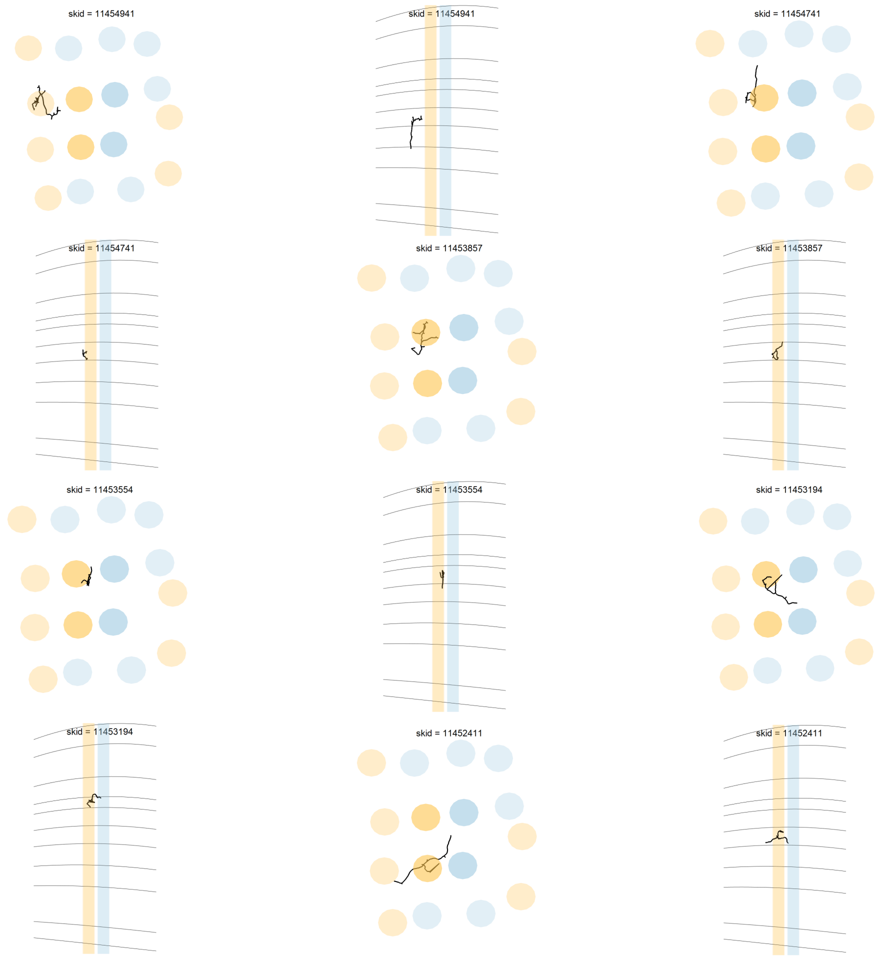
skid = 11454941
skid = 11454941
skid = 11454741
skid = 11454741
skid = 11453857
skid = 11453857
skid = 11453554
skid = 11453554
skid = 11453194
skid = 11453194
skid = 11452411
skid = 11452411

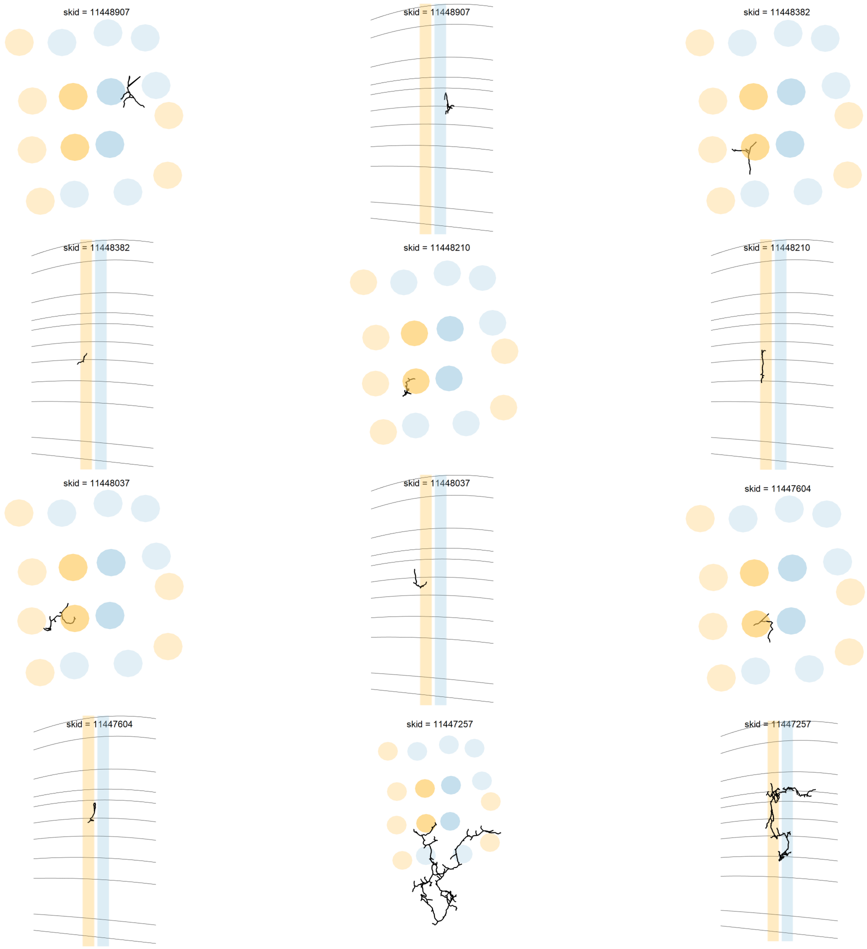
skid = 11448907
skid = 11448907
skid = 11448382
skid = 11448382
skid = 11448210
skid = 11448210
skid = 11448037
skid = 11448037
skid = 11447604
skid = 11447604
skid = 11447257
skid = 11447257

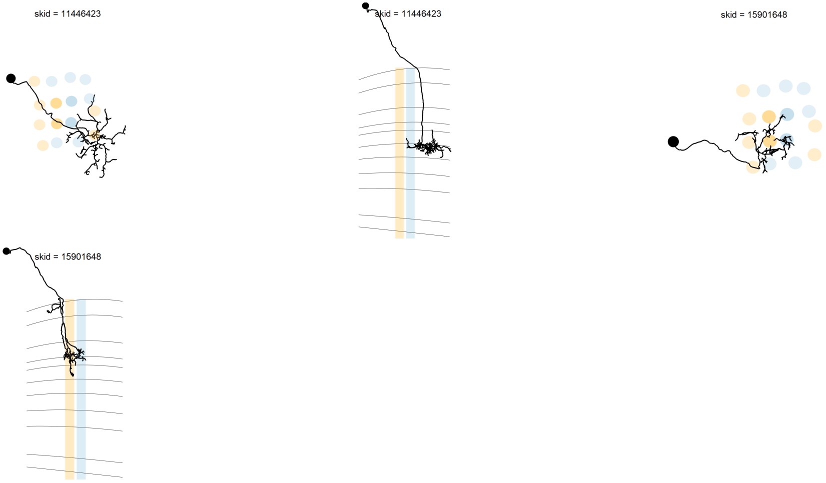
skid = 11446423
skid = 11446423
skid = 15901648
skid = 15901648

DRA seed column Dm-DRA1 (20 cells)

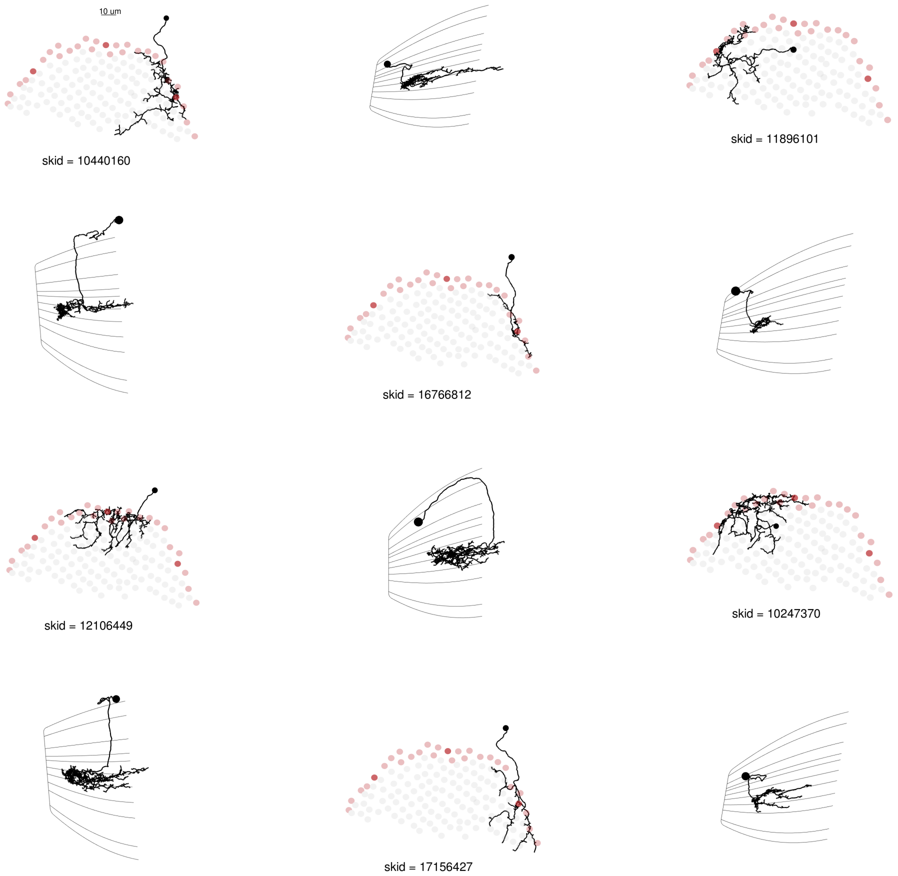

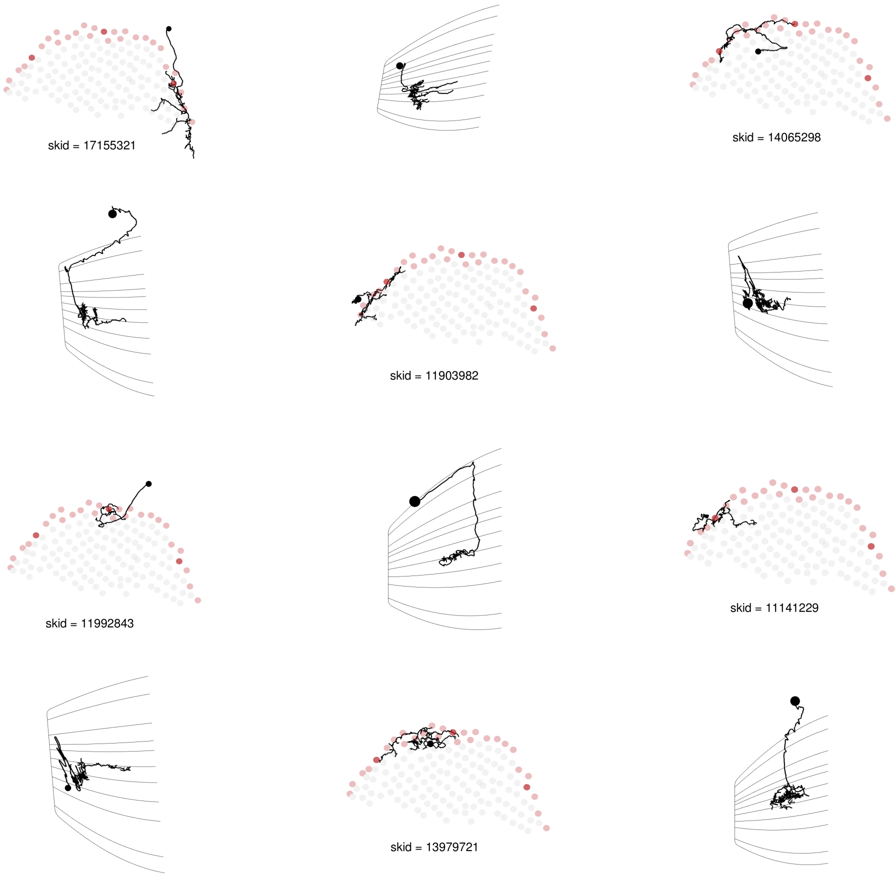
skid = 17155321
skid = 14065298
skid = 11903982
skid = 11992843
skid = 11141229
skid = 13979721

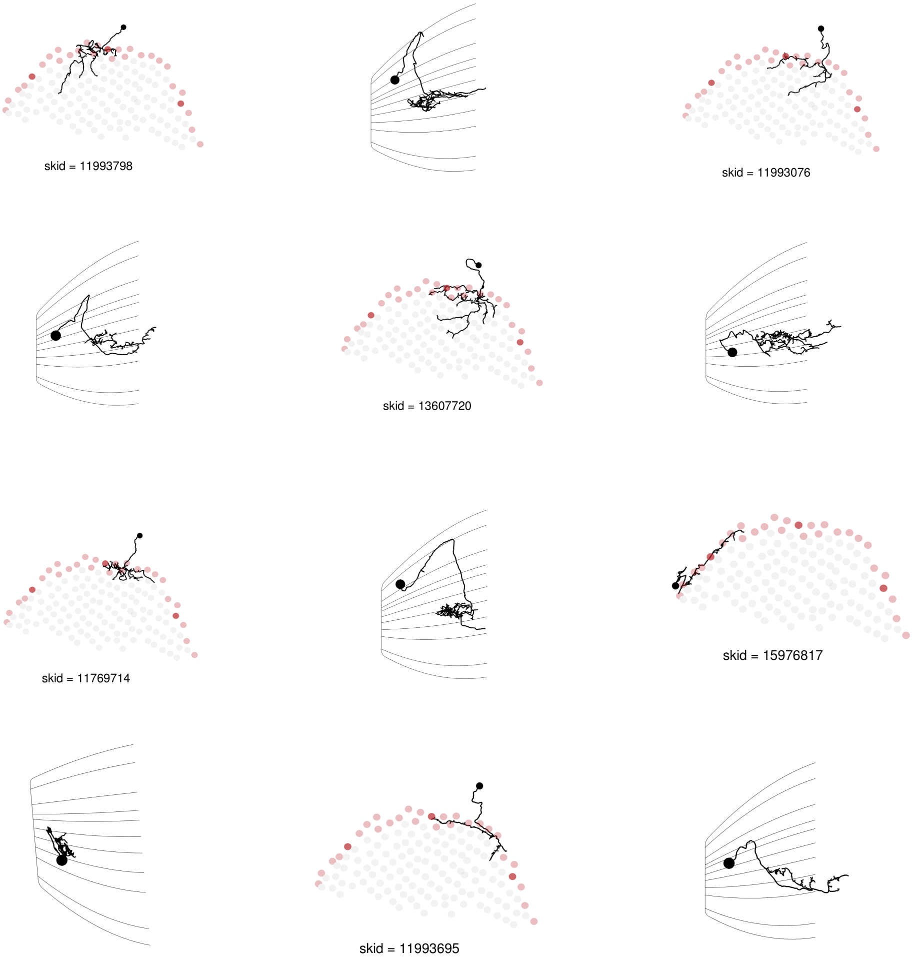
skid = 11993798
skid = 11993076
skid = 13607720
skid = 11769714
skid = 15976817
skid = 11993695

DRA seed column Dm9 (6 cells)

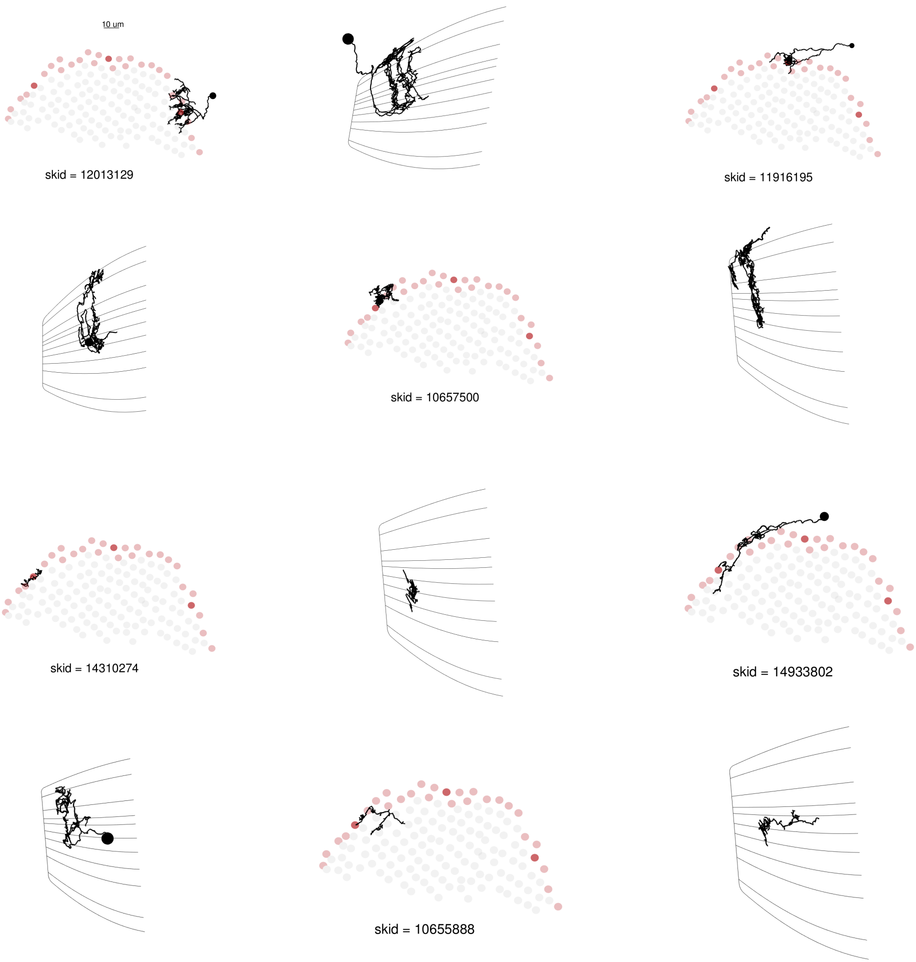

DRA seed column MeTu-DRA (30 cells)

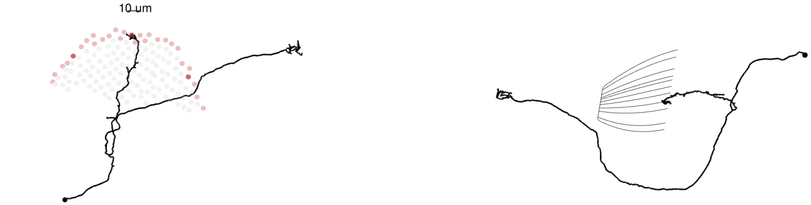

skid = 11995073

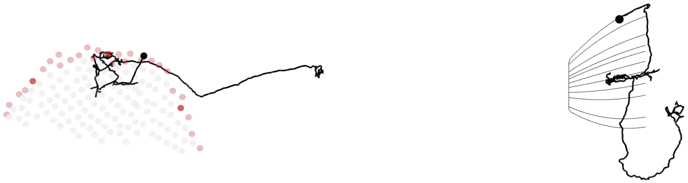

skid = 11994448

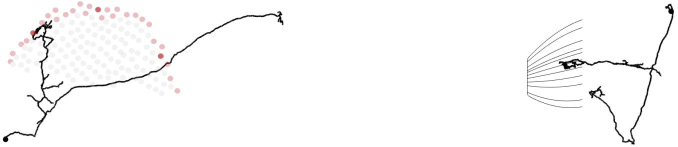

skid = 11908710

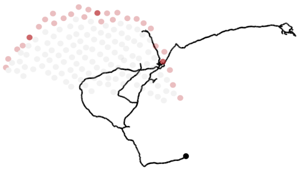

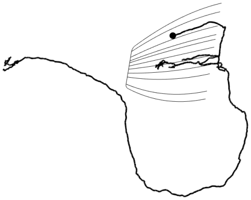

skid = 11695293

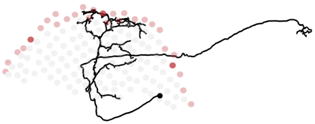

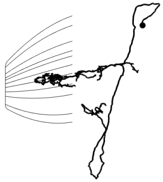

skid = 15749047

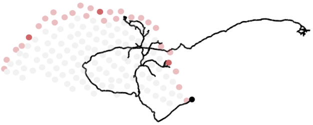

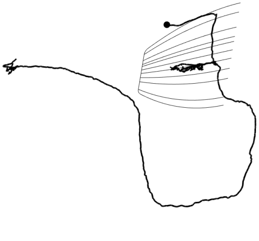

skid = 12106383

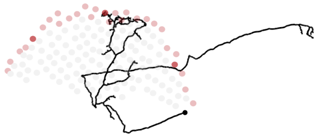

skid = 11994563

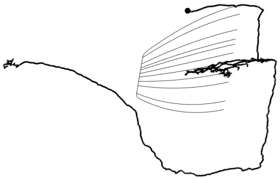

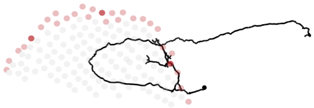

skid = 10438339

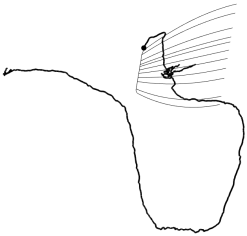

Lorem ipsum

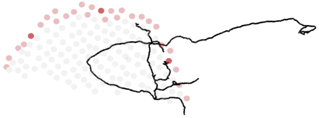

skid = 15952007

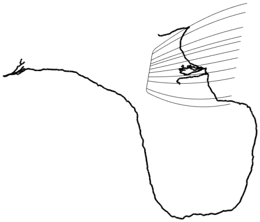

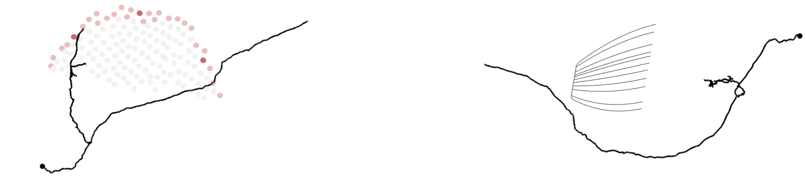

skid = 13136156

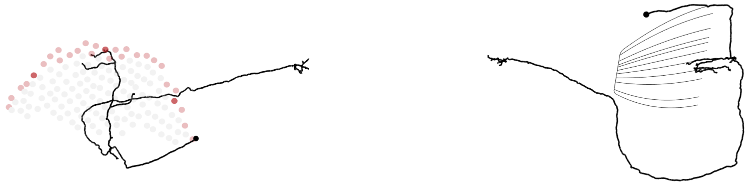

skid = 11995102

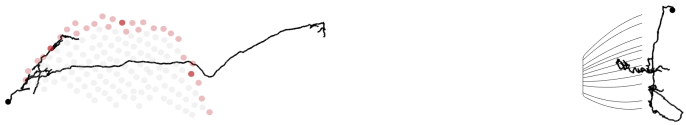

skid = 15616477

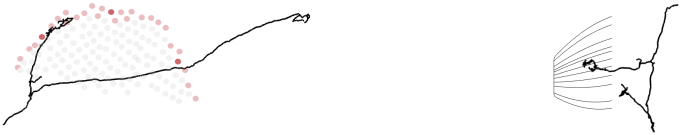

skid = 15600898

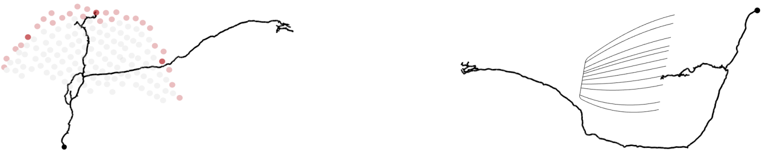

skid = 11993030

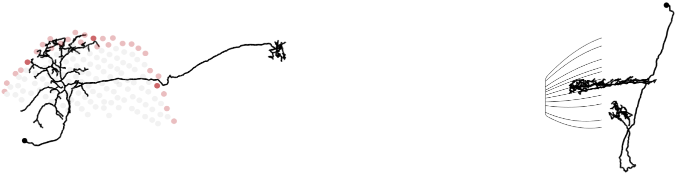

skid = 15698953

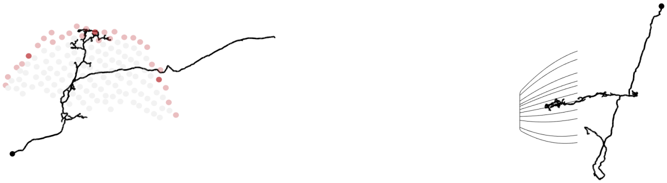

skid = 12127369

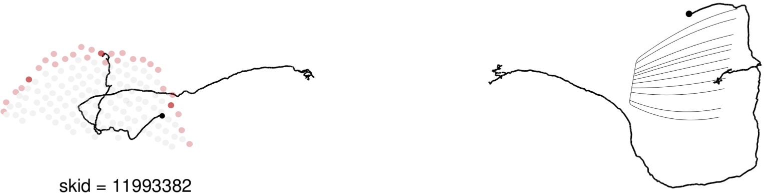

skid = 11993382

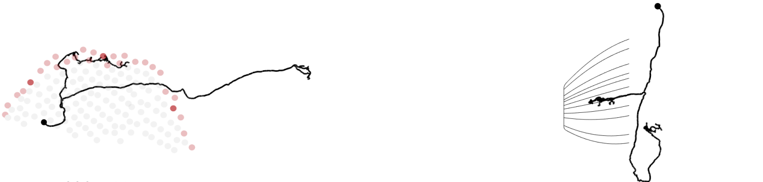

skid = 11992932

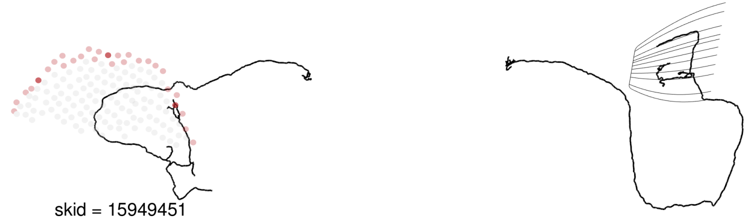
skid = 15949451

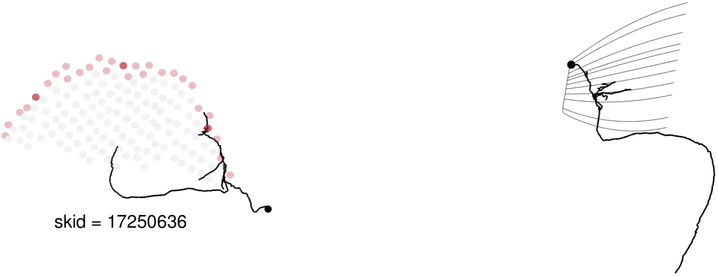
skid = 17250636

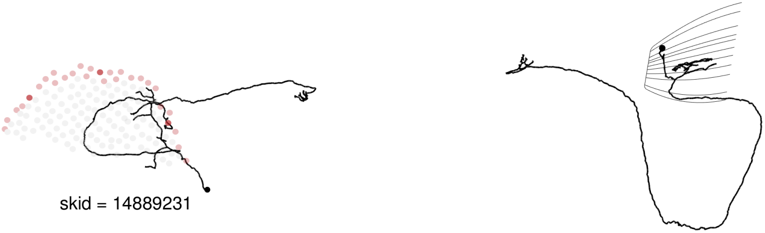
skid = 14889231

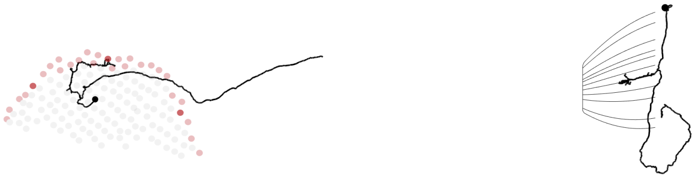

skid = 11995358

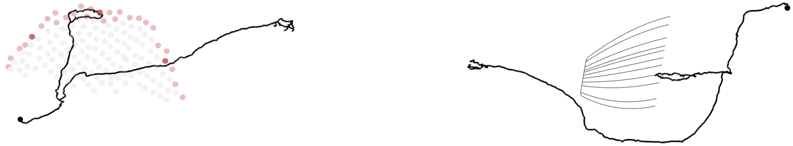

skid = 11993352

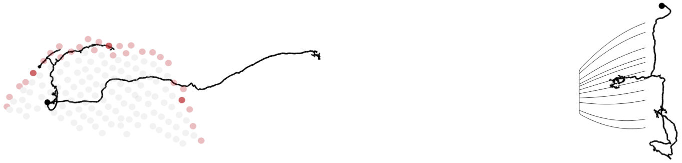

skid = 11993314

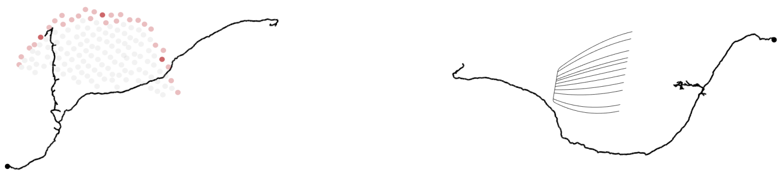

skid = 11908743

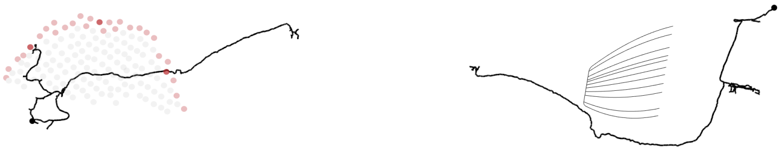

skid = 14864504

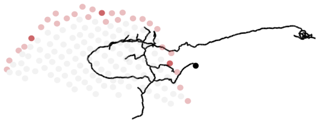

skid = 14936634

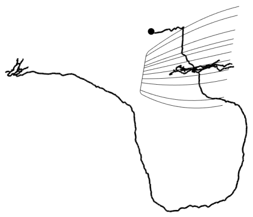

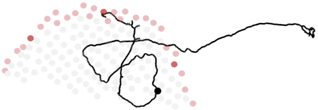

skid = 11993064

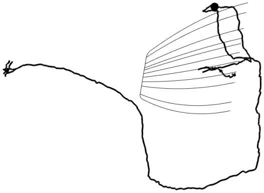

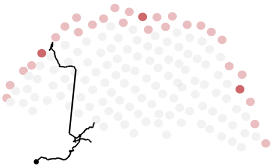

skid = 11908668

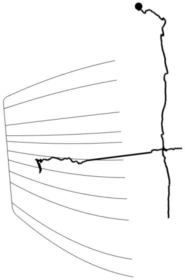

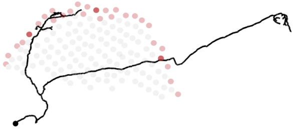

skid = 11903992

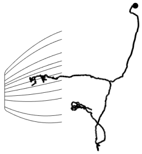

DRA seed column R7-DRA (3 cells)

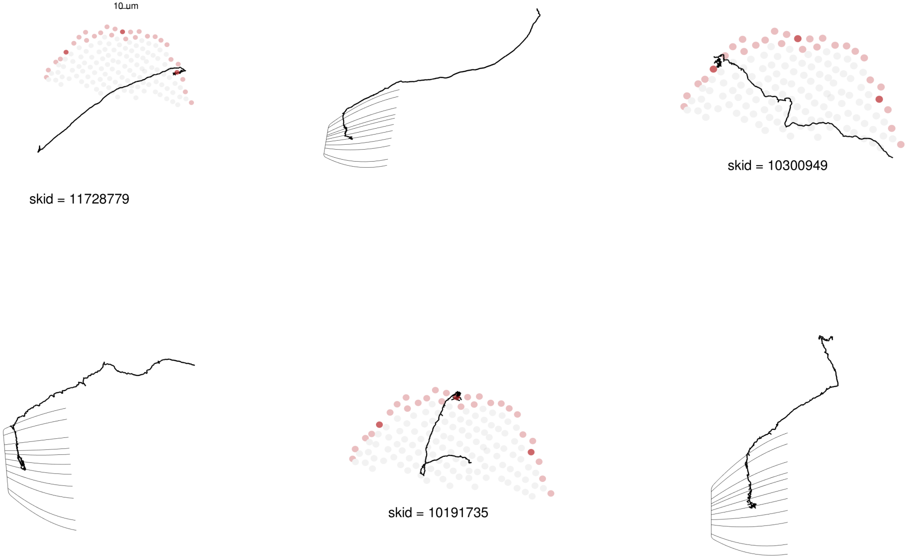

DRA seed column Dm-DRA2 (9 cells)

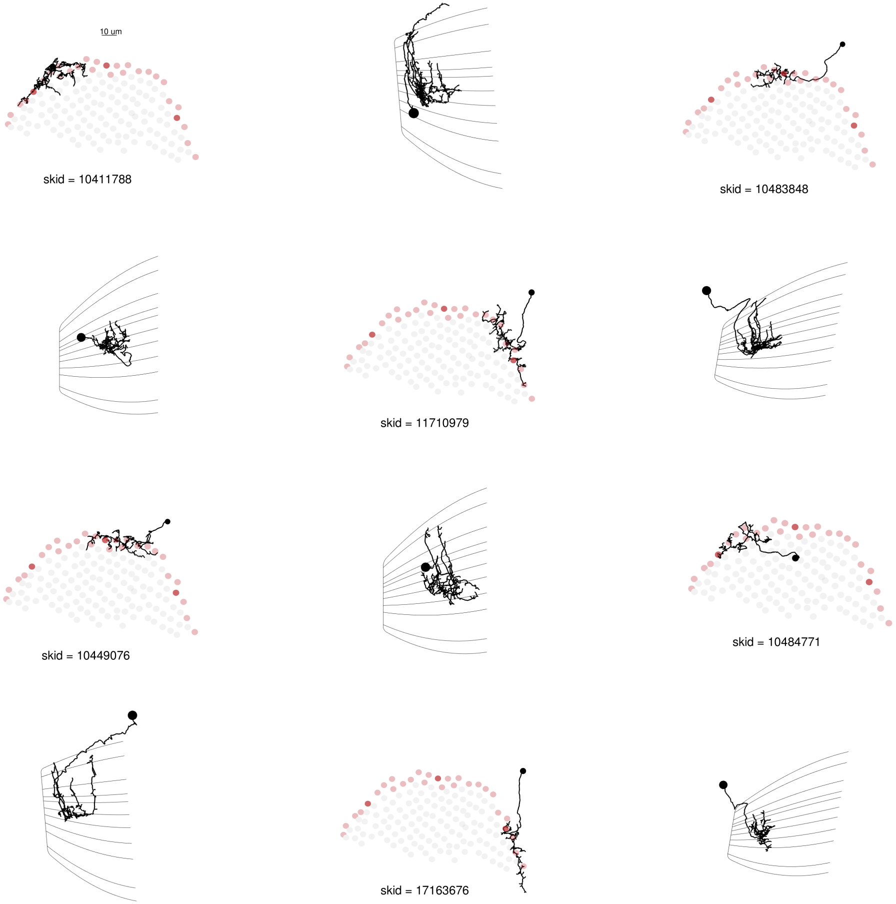

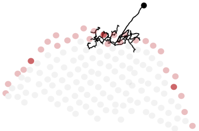

skid = 13262259

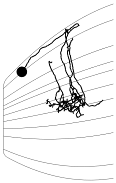

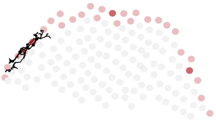

skid = 14311483

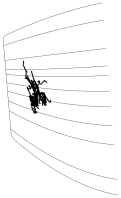

DRA seed column Dm2 (4 cells)

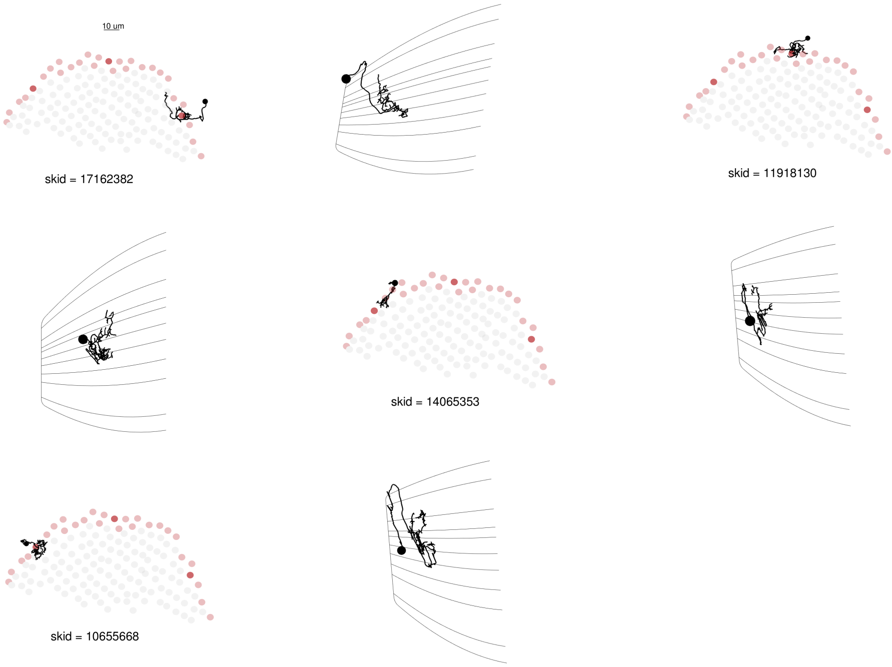

DRA seed column R8-DRA (3 cells)

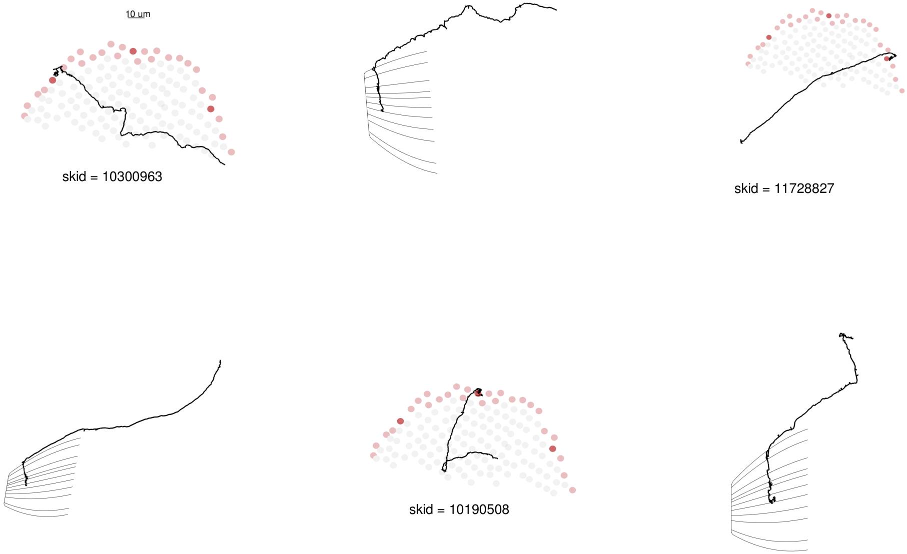

DRA seed column Mi15 (4 cells)

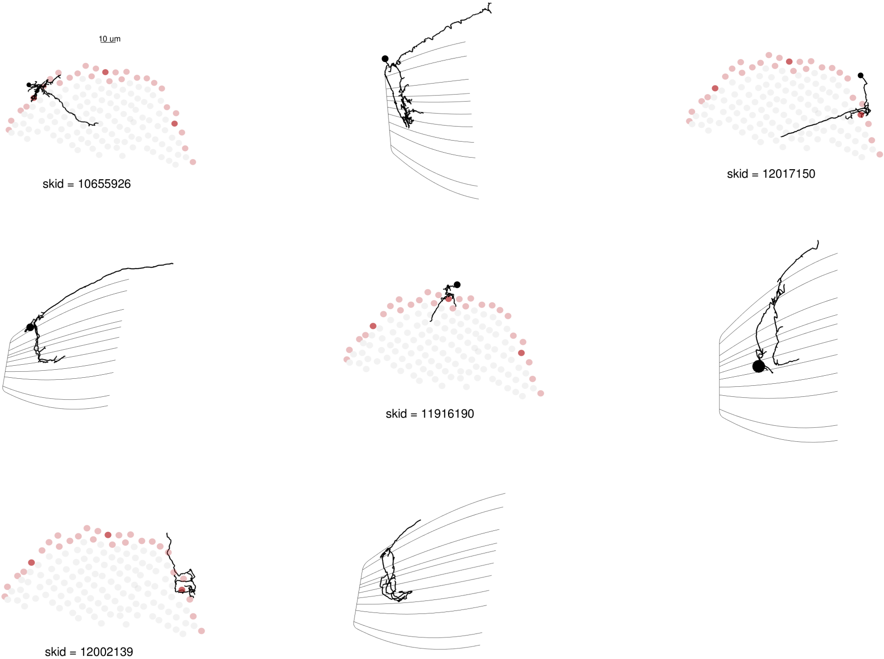

DRA seed column Mti-DRA-1 (6 cells)

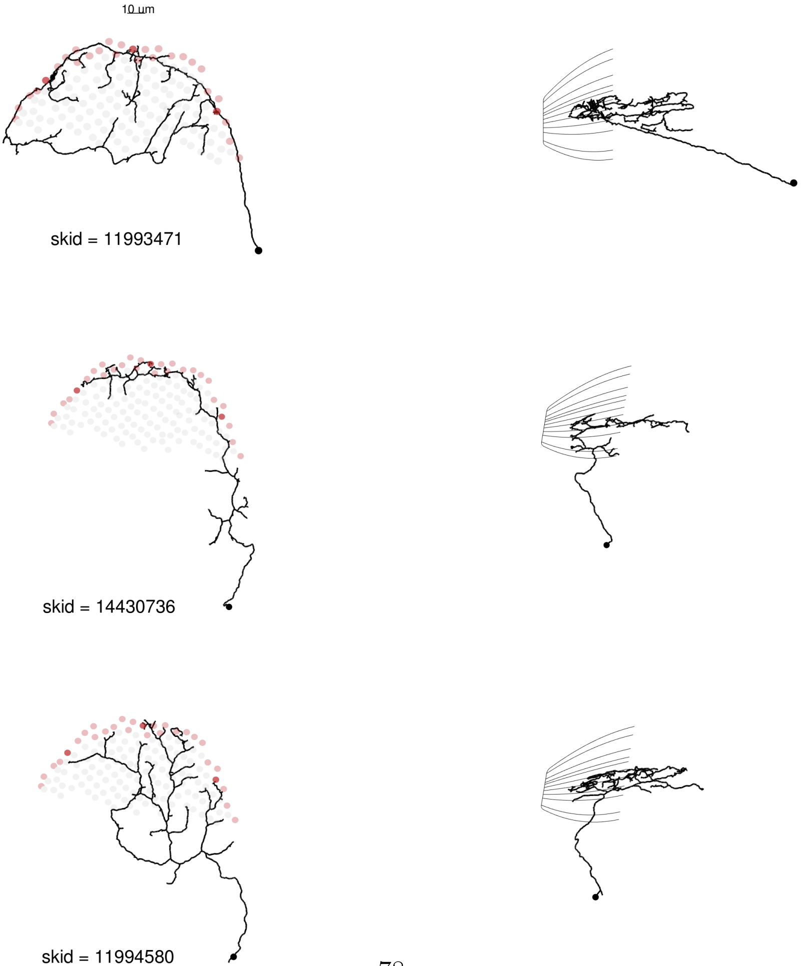

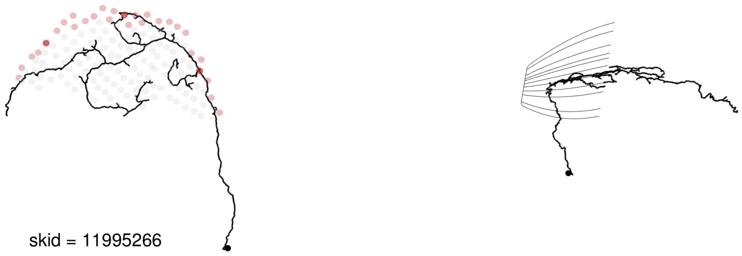
skid = 11995266

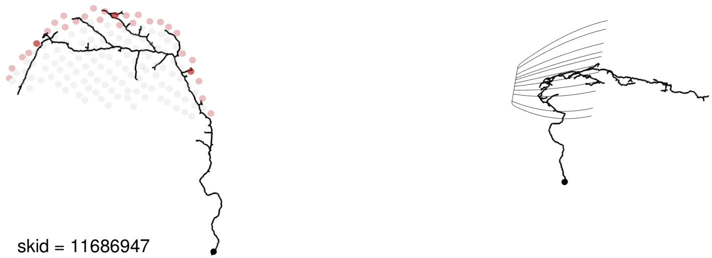
skid = 11686947

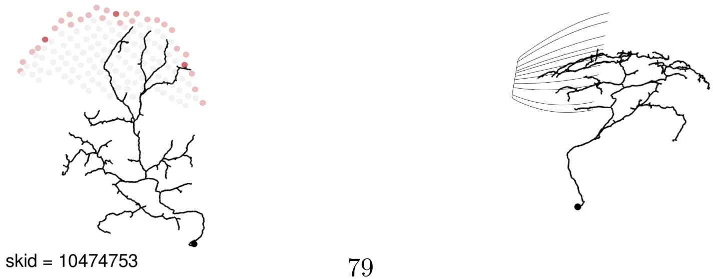
skid = 10474753

DRA seed column MeMe-DRA (2 cells)

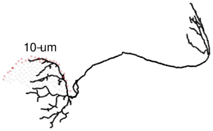

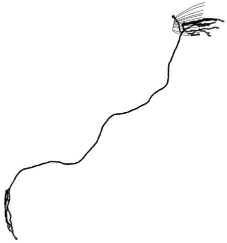

skid = 10439442

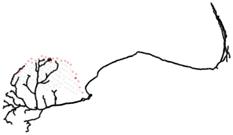

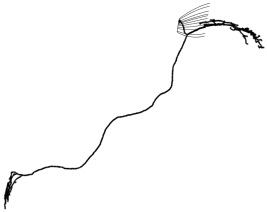

skid = 11993543

DRA seed column L3 (3 cells)

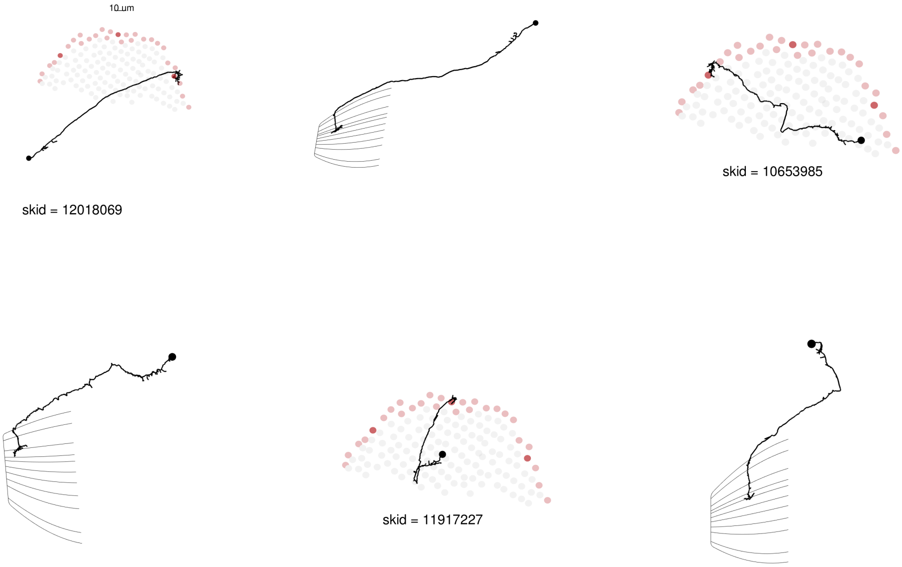

DRA seed column VPN-DRA (6 cells)

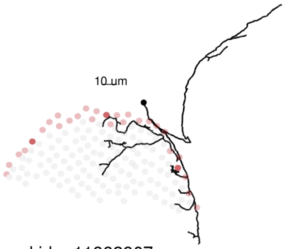

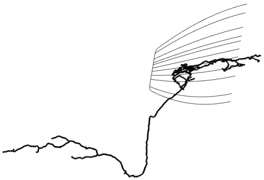

skid = 11992907

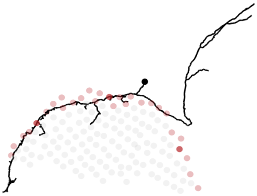

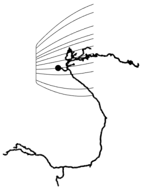

skid = 11904073

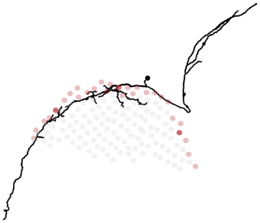

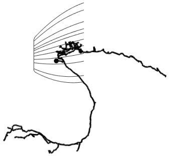

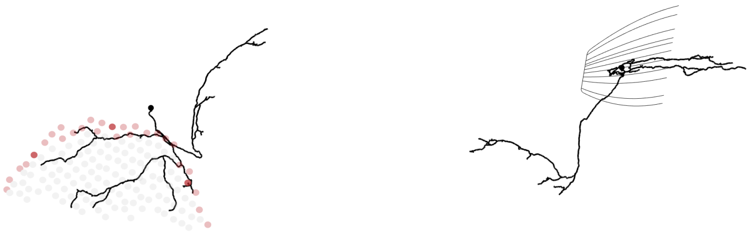

skid = 17165715

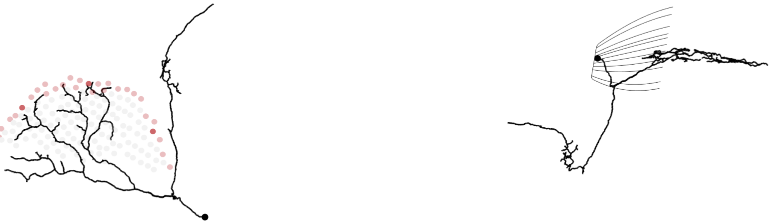

skid = 15969128

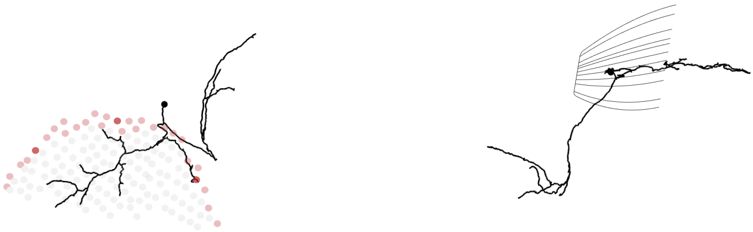

skid = 16886430

DRA seed column L1 (3 cells)

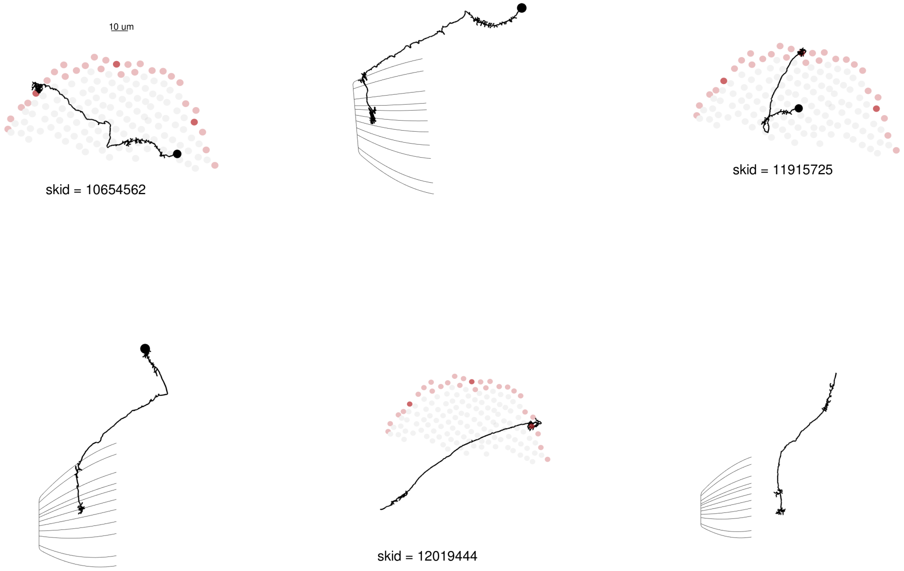

DRA seed column Tm20 (3 cells)

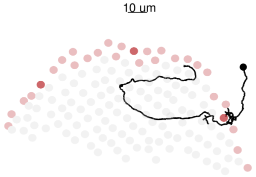

skid = 12018769

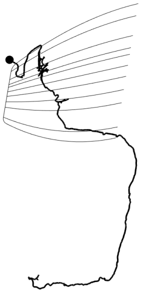

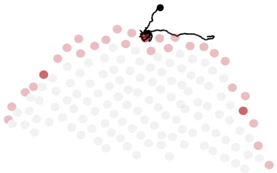

skid = 11918619

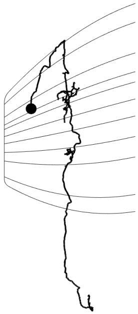

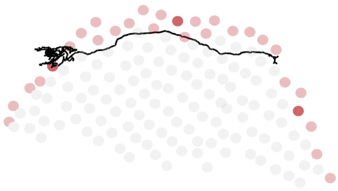

skid = 10655481

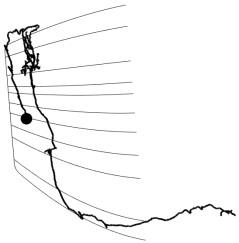

DRA seed column Mti-DRA-2 (4 cells)

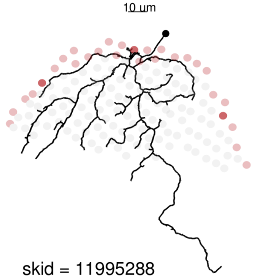

skid = 11995288

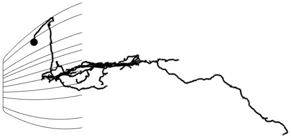

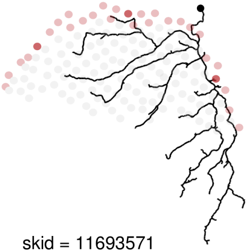

skid = 11693571

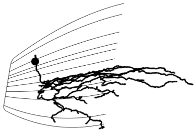

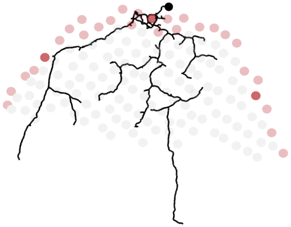

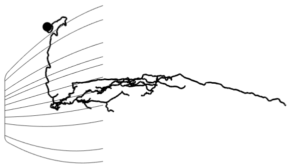

skid = 11993444

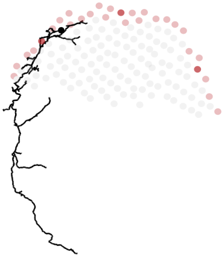

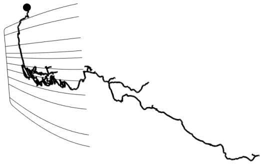

skid = 11903802

DRA seed column Mi1 (3 cells)

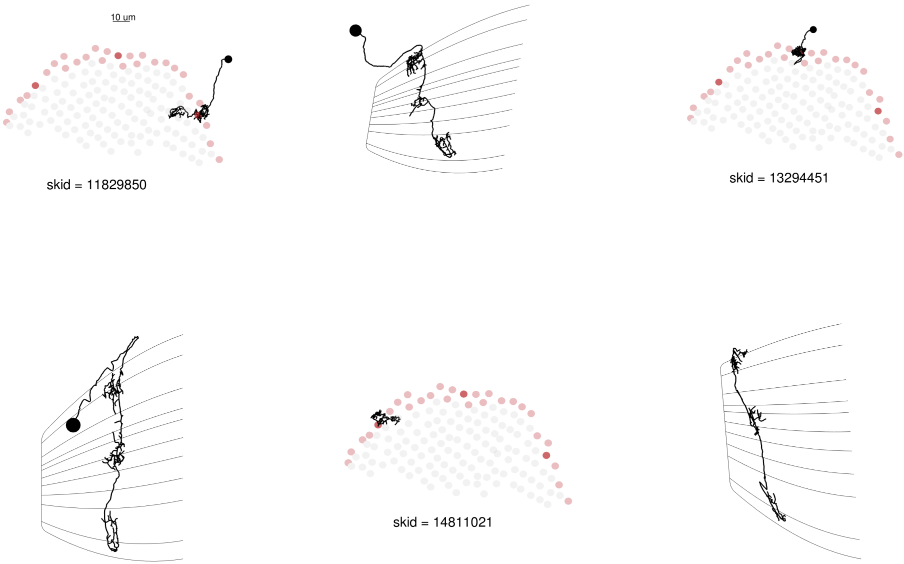

DRA seed column MeTu (2 cells)

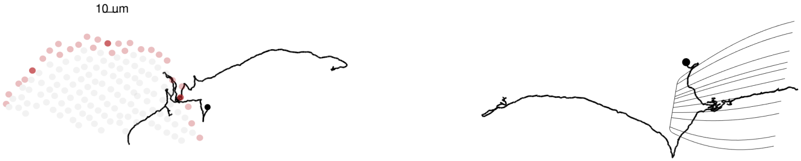

skid = 16730682

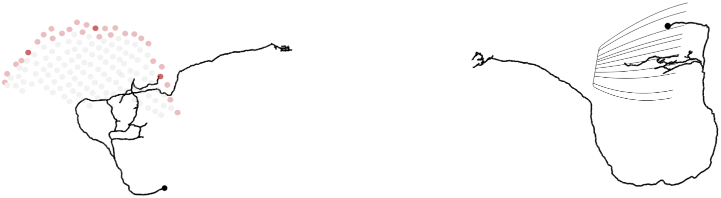

skid = 14888320

DRA seed column Tm5-like (1 cell)

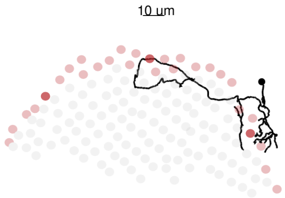

skid = 12016006

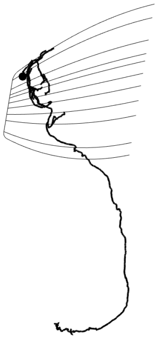

DRA seed column Mi9 (2 cells)

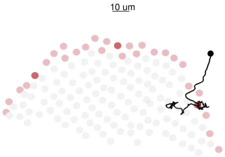

skid = 12015966

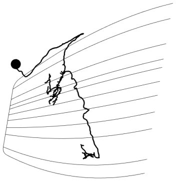

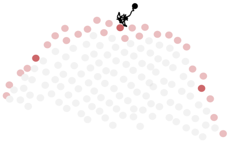

skid = 11918590

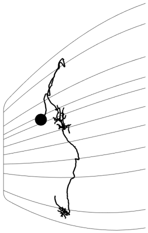

DRA seed column Dm11 (1 cell)

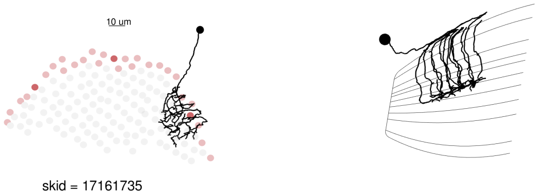

DRA seed column aMe12 (1 cell)

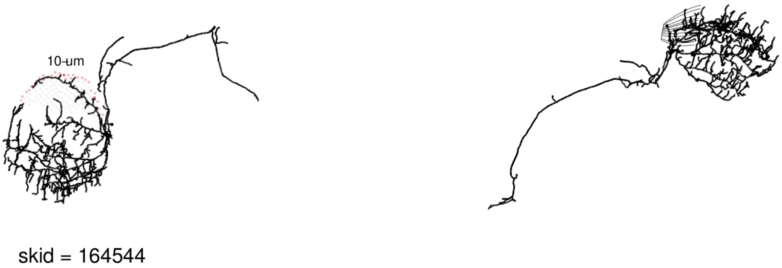

DRA seed column TmY (1 cell)

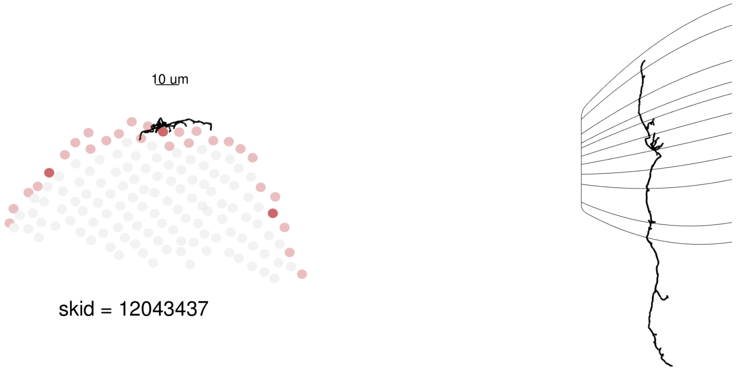

DRA seed column ML-VPN2 (1 cell)

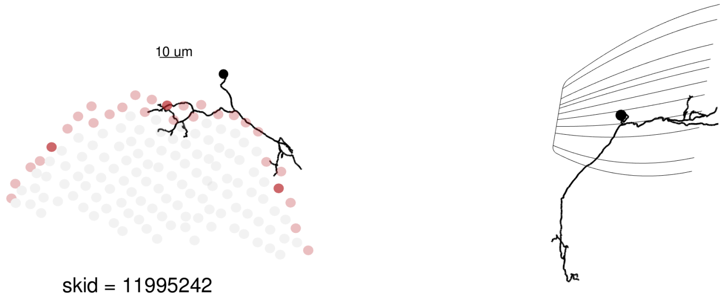

DRA seed column C2 (1 cell)

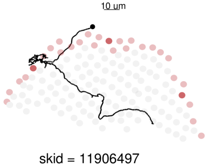

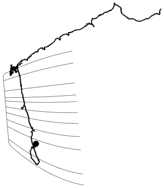

DRA seed column Identified < 3 synapses (33 cells)

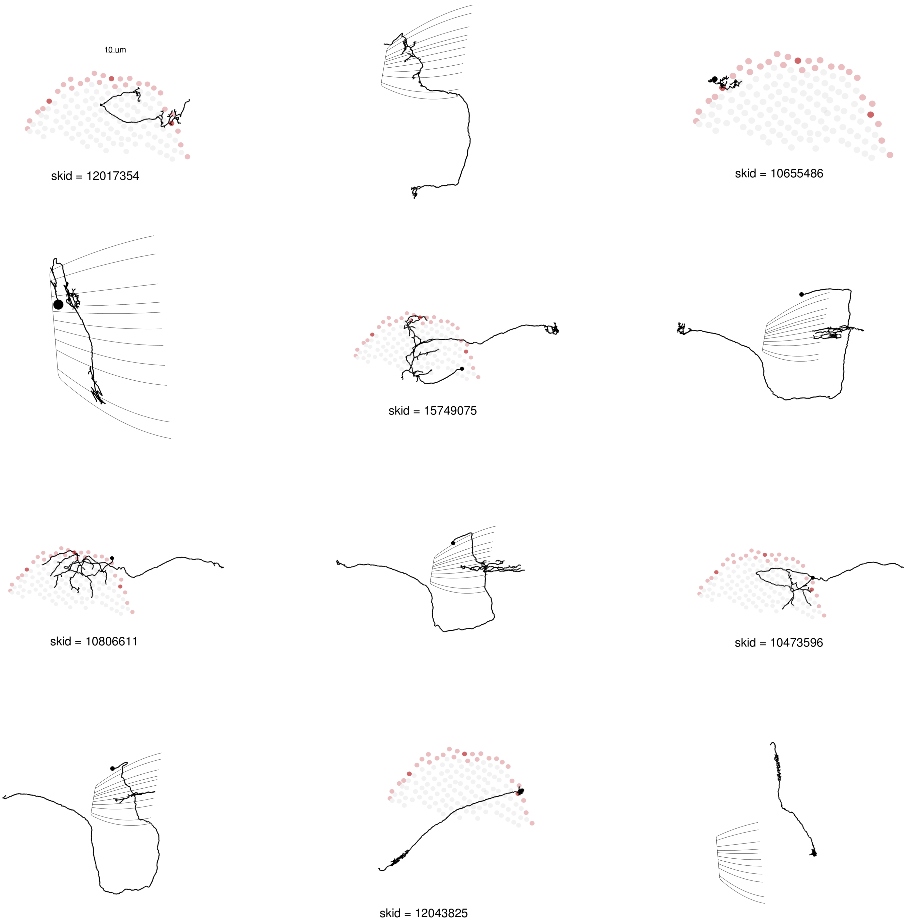

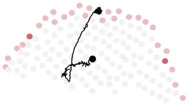

skid = 11915906

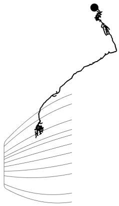

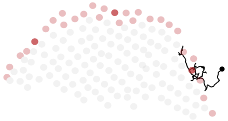

skid = 17254550

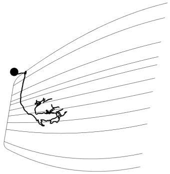

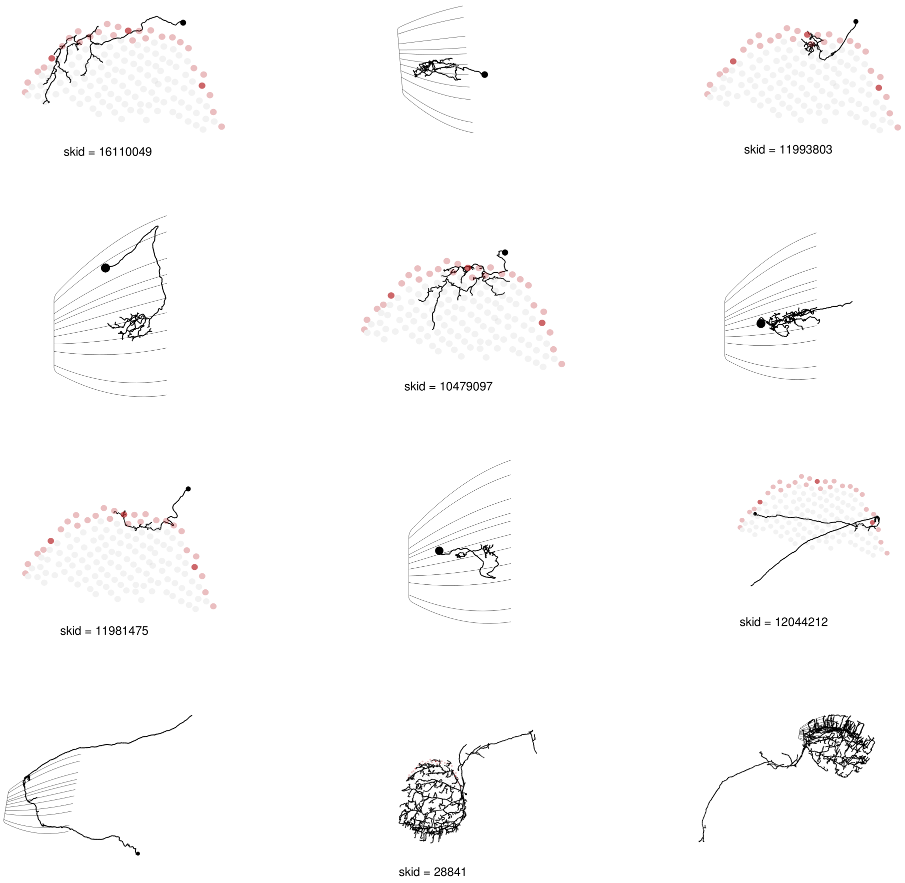
skid = 16110049
skid = 11993803
skid = 10479097
skid = 11981475
skid = 12044212
skid = 28841

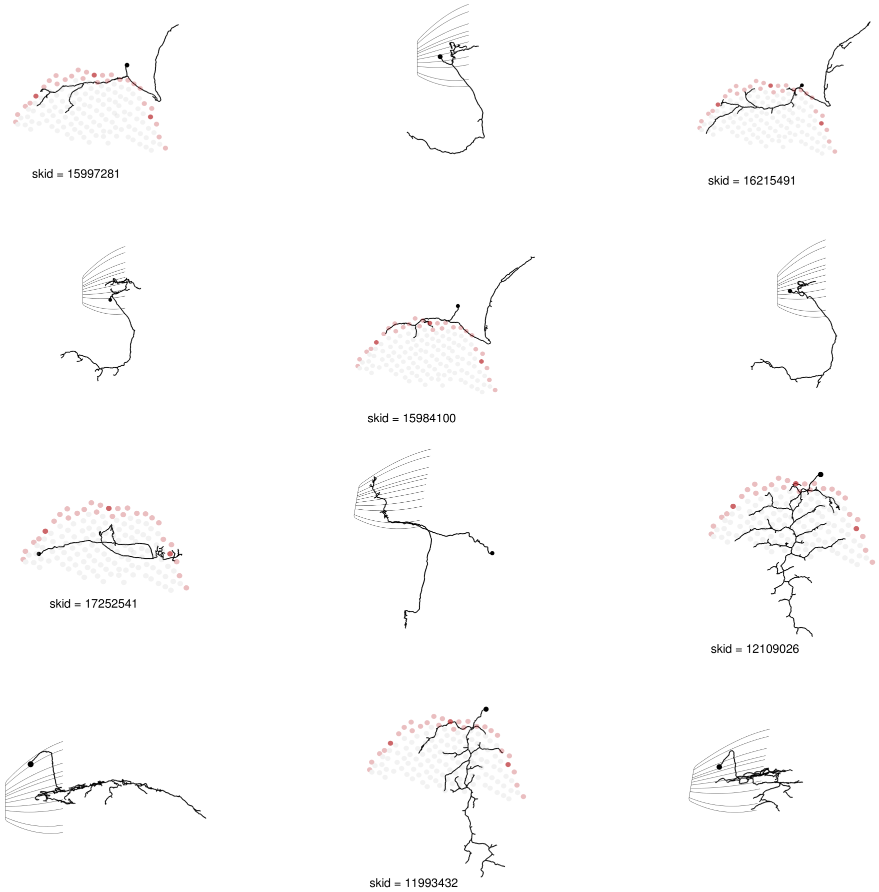
skid = 15997281
skid = 16215491
skid = 15984100
skid = 17252541
skid = 12109026
skid = 11993432

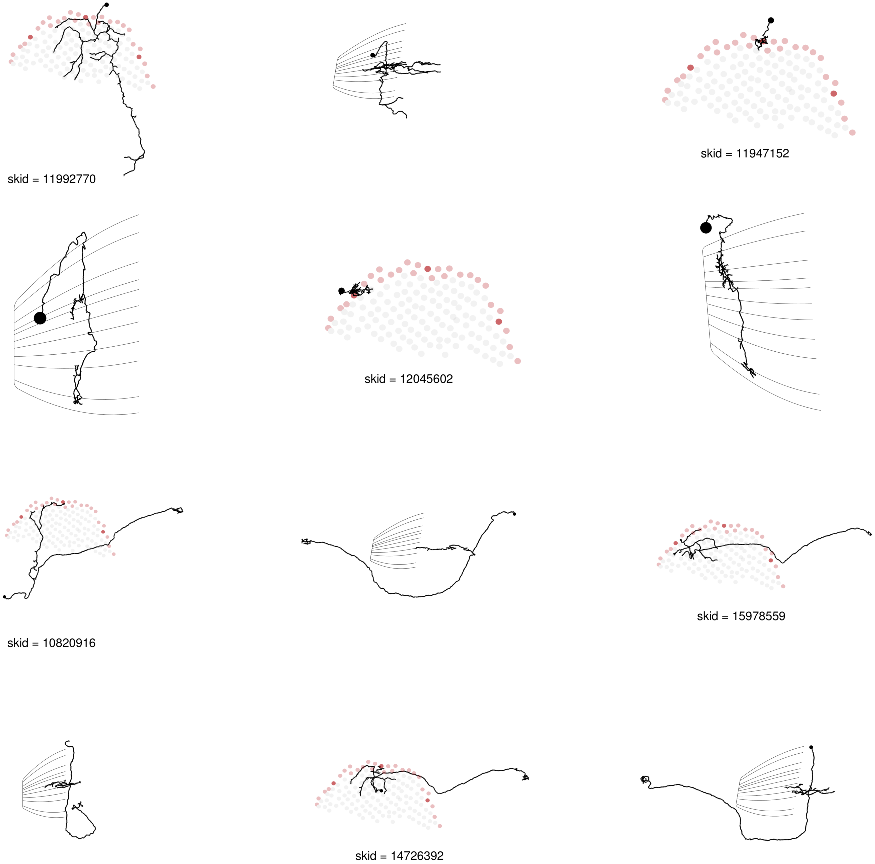

skid = 11992770
skid = 11947152
skid = 12045602
skid = 10820916
skid = 15978559
skid = 14726392

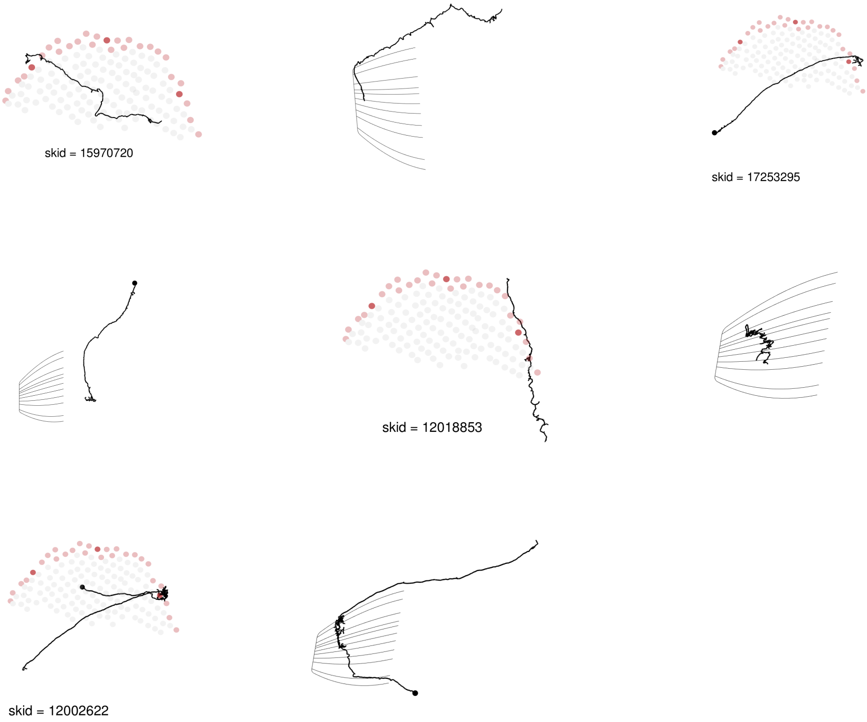
skid = 15970720
skid = 17253295
skid = 12018853
skid = 12002622

DRA seed column Unidentified ≥ 3 synapses (2 cells)

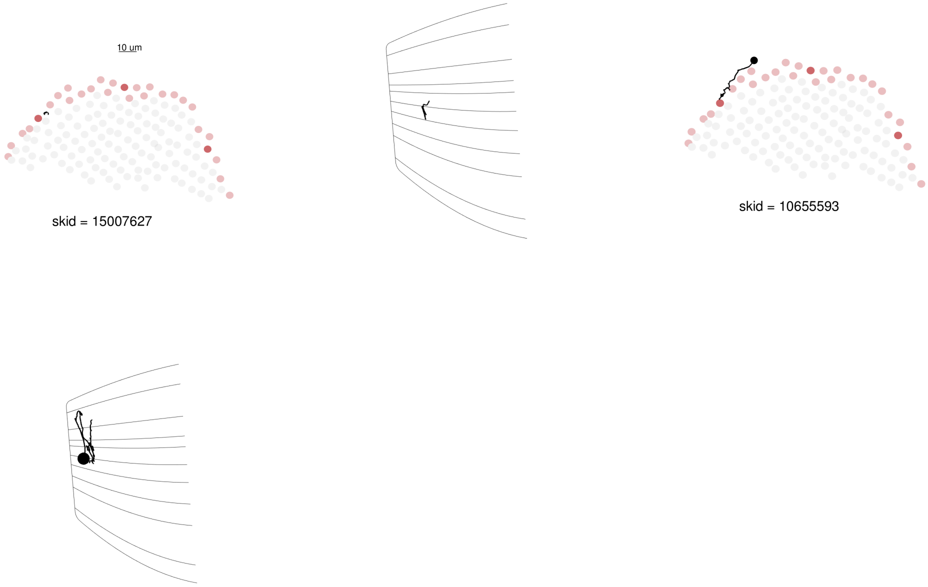

DRA seed column Unidentified < 3 synapses (57 cells)

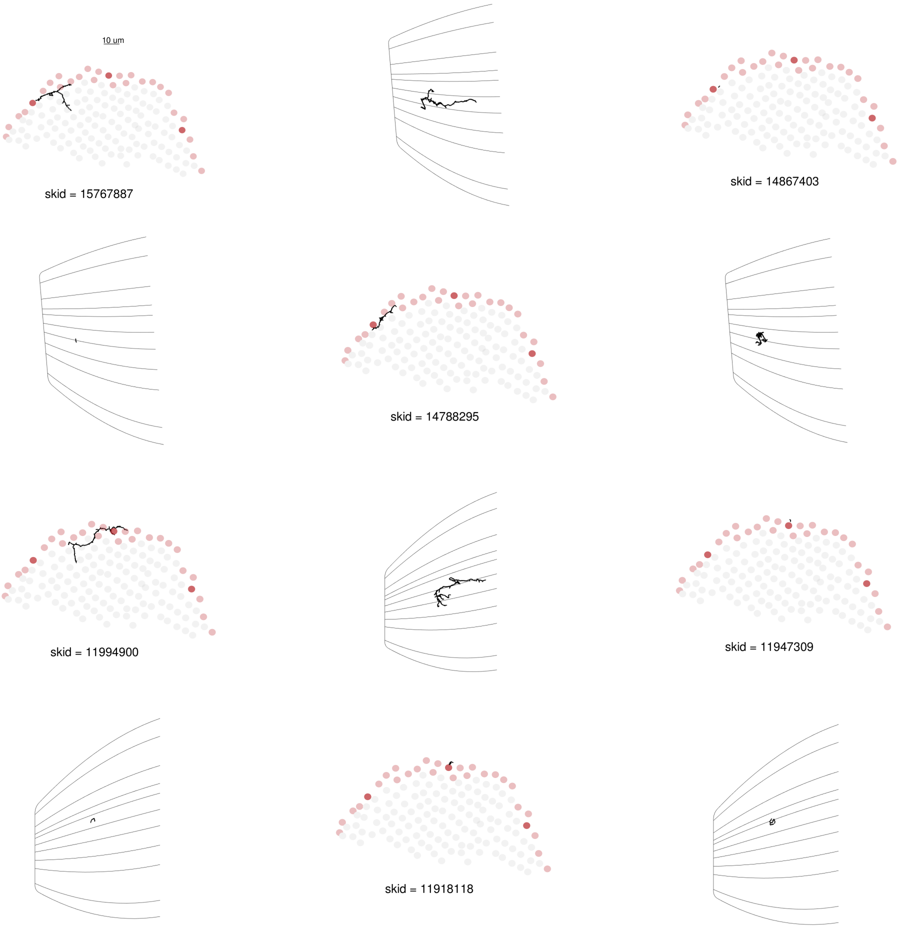

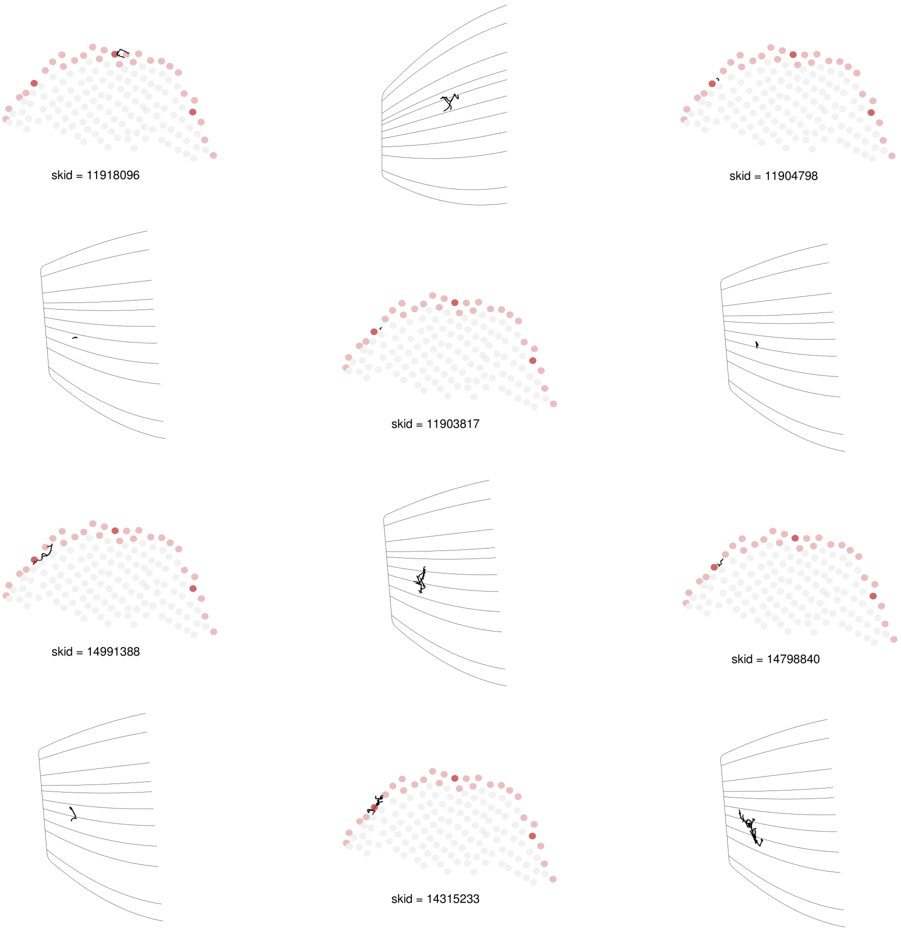
skid = 11918096
skid = 11904798
skid = 11903817
skid = 14991388
skid = 14798840
skid = 14315233

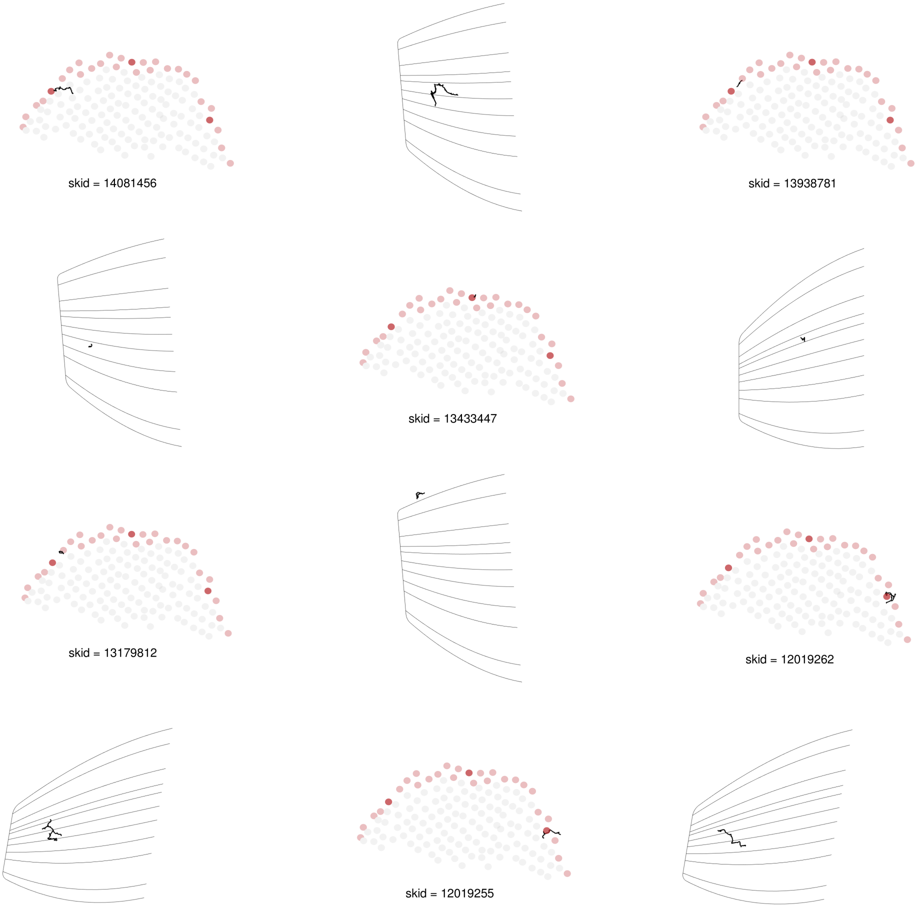
skid = 14081456
skid = 13938781
skid = 13433447
skid = 13179812
skid = 12019262
skid = 12019255

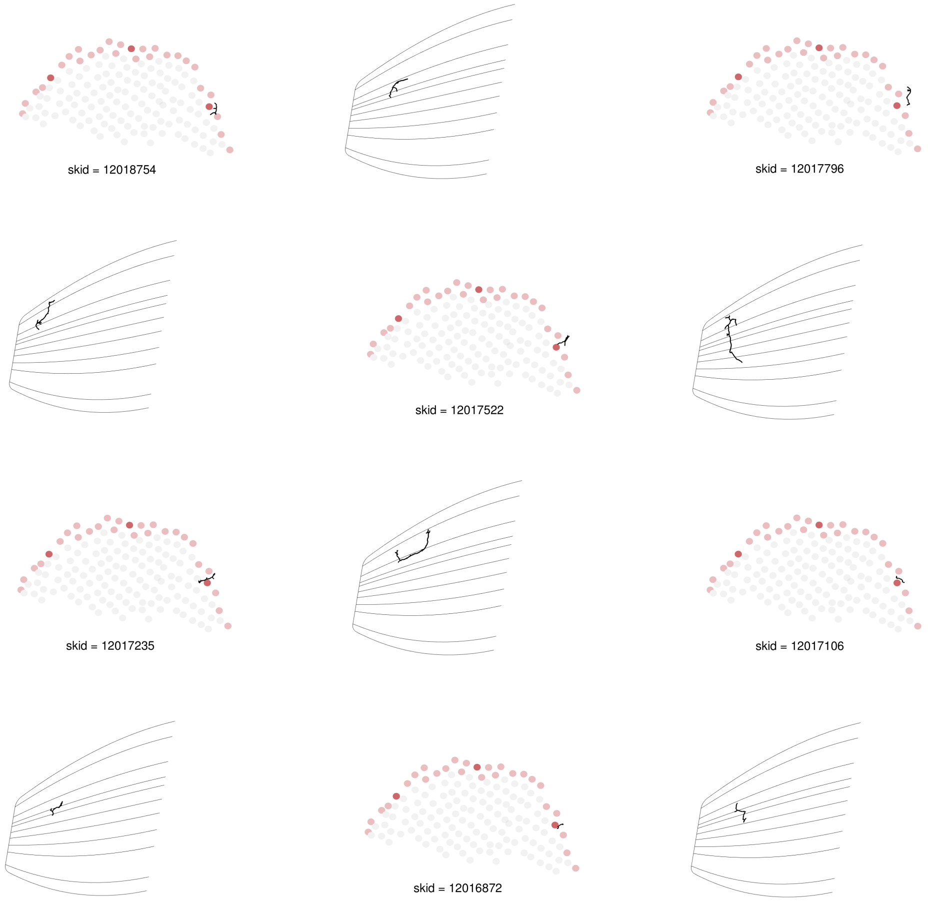
skid = 12018754
skid = 12017796
skid = 12017522
skid = 12017235
skid = 12017106
skid = 12016872

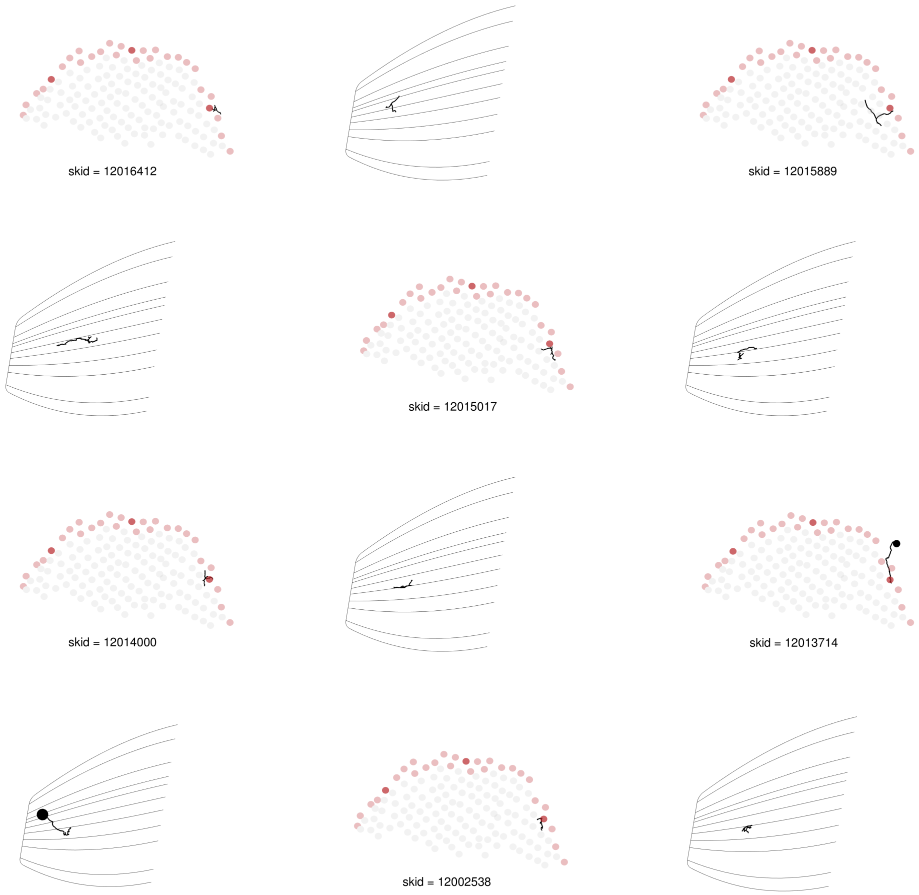
skid = 12016412
skid = 12015889
skid = 12015017
skid = 12014000
skid = 12013714
skid = 12002538

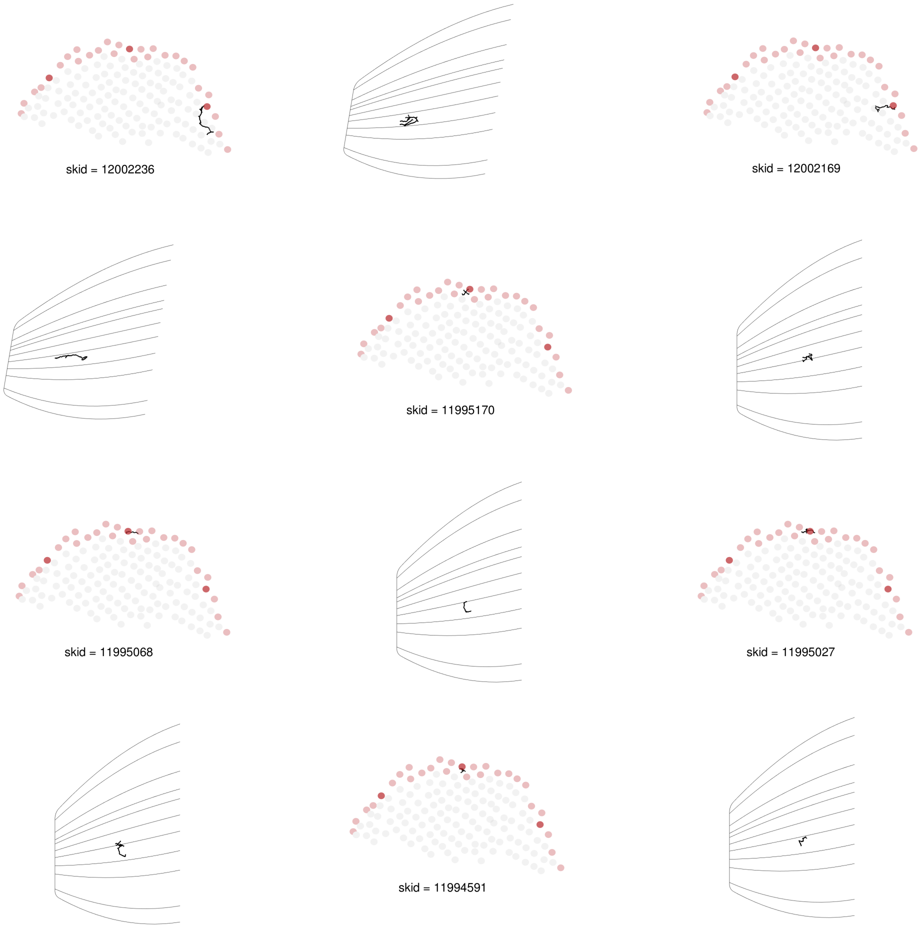
skid = 12002236
skid = 12002169
skid = 11995170
skid = 11995068
skid = 11995027
skid = 11994591

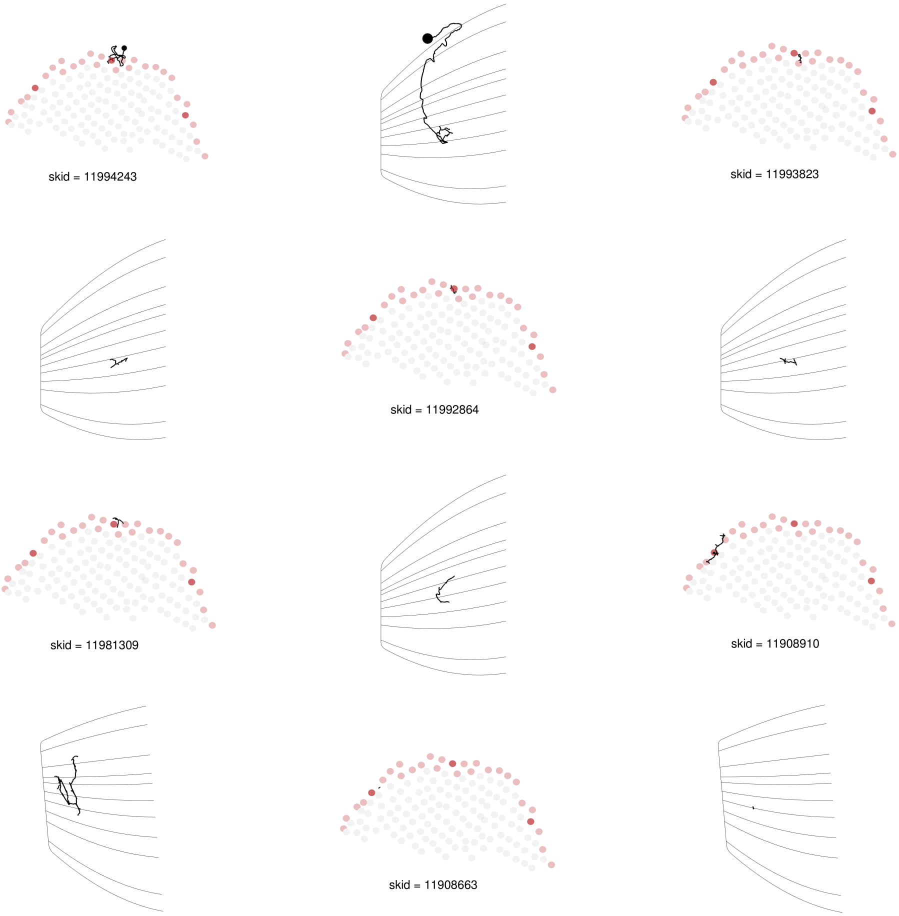
skid = 11994243
skid = 11993823
skid = 11992864
skid = 11981309
skid = 11908910
skid = 11908663

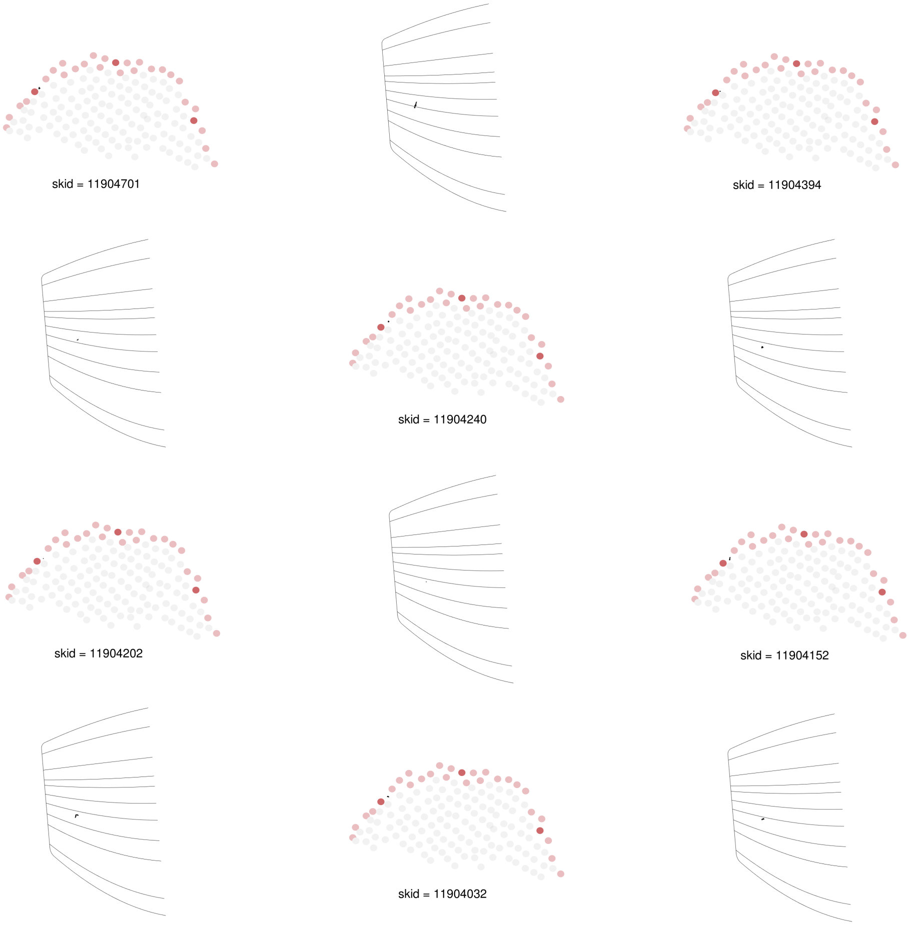
skid = 11904701
skid = 11904394
skid = 11904240
skid = 11904202
skid = 11904152
skid = 11904032

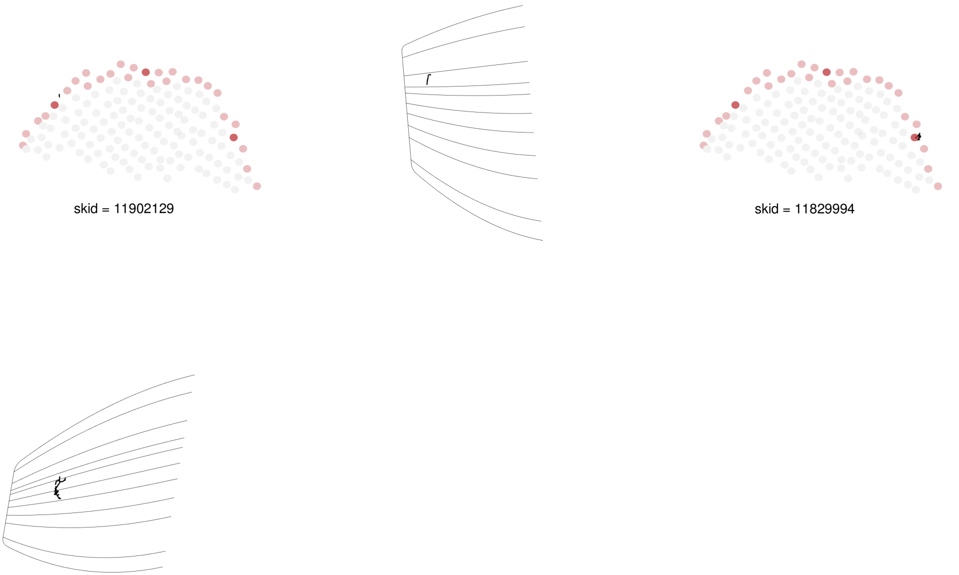
skid = 11902129
skid = 11829994
