# Supplementary material for: Synaptic targets of photoreceptors specialized to detect color and skylight polarization in Drosophila
Source: eLife. 2021 Dec 16;10:e71858. doi: 10.7554/eLife.71858 (PMC8789284; doi:10.7554/eLife.71858)
Supplement: Supplementary file 3. [file elife-71858-supp3.pdf]

Supplementary File 3: Tables of seed column synapses outside the medulla

Contents

R7 and R8 outgoing 2
R7 and R8 incoming 3
R7-DRA and R8-DRA outgoing 4
R7-DRA and R8-DRA outgoing 5

R7 and R8 outgoing

Type	No.	pR7	yR7	pR8	yR8	Sum
Dm9	6	13	9	6	6	34
Dm8	15	0	0	0	0	0
MeTu	7	0	0	0	0	0
R7	5	0	0	16	25	41
Tm5c	6	0	5	2	9	16
Tm20	4	0	1	0	1	2
Mi15	4	5	5	15	35	60
Mi4	4	0	0	0	4	4
ML1	4	0	0	0	0	0
Dm2	4	0	0	0	0	0
Dm11	2	16	23	1	4	44
L3	4	15	17	7	10	49
Mi1	4	0	0	1	1	2
R8	4	22	24	0	0	46
Tm5a	2	0	0	0	0	0
Tm5b	2	0	0	0	0	0
Tm	9	0	0	0	0	0
Tm5b-like	3	0	0	0	0	0
Mi9	4	0	0	0	0	0
L1	4	2	1	10	7	20
aMe12	2	0	0	0	0	0
Dm	5	0	0	0	0	0
ML-VPN1	3	0	0	0	0	0
C2	2	1	0	4	0	5
Mt-VPN	4	0	1	0	7	8
Mti	3	0	0	0	0	0
Tm5a-like	1	0	0	0	0	0
TmY10	1	0	0	0	0	0
Mi10	1	0	0	0	0	0
Mi	1	0	0	0	0	0
C3	1	0	0	0	0	0
Identified <3	26	0	0	2	0	2
Unidentified >=3	2	0	0	0	2	2
Unidentified <3	62	1	0	0	3	4
Total	211	75	86	64	114	339

R7 and R8 incoming

Type	No.	pR7	yR7	pR8	yR8	Sum
Dm9	6	8	4	5	1	18
R8	4	16	25	0	0	41
R7	4	0	0	22	24	46
Mt-VPN	1	0	2	0	3	5
C2	1	0	0	0	0	0
L3	1	0	0	0	0	0
Identified <3	11	1	0	2	5	8
Unidentified >=3	0	0	0	0	0	0
Unidentified <3	4	0	2	0	0	2
Total	32	25	33	29	33	120

R7-DRA and R8-DRA outgoing

Type	No.	R7-DRA	R8-DRA	Sum
Dm-DRA1	20	0	0	0
Dm9	6	1	1	2
MeTu-DRA	30	0	0	0
R7-DRA	3	0	16	16
Dm-DRA2	9	0	6	6
Dm2	4	0	0	0
R8-DRA	3	19	0	19
Mi15	4	12	8	20
Mti-DRA-1	6	0	0	0
MeMe-DRA	2	0	0	0
L3	3	10	8	18
VPN-DRA	6	0	0	0
L1	3	4	3	7
Tm20	3	0	0	0
Mti-DRA-2	4	0	0	0
Mi1	3	0	0	0
MeTu	2	0	0	0
Tm5-like	1	0	0	0
Mi9	2	0	0	0
Dm11	1	0	0	0
aMe12	1	0	0	0
TmY	1	0	0	0
ML-VPN2	1	0	0	0
C2	1	2	1	3
Identified <3	33	3	2	5
Unidentified >=3	2	0	0	0
Unidentified <3	57	0	0	0
Total	211	51	45	96

R7-DRA and R8-DRA outgoing

Type	No.	R7-DRA	R8-DRA	Sum
Dm9	4	0	0	0
R8-DRA	3	16	0	16
R7-DRA	3	0	19	19
C2	2	3	3	6
Mi15	1	2	2	4
Identified <3	9	0	0	0
Unidentified >=3	0	0	0	0
Unidentified <3	2	1	1	2
Total	24	22	25	47
