# Supplementary material for: Synaptic targets of photoreceptors specialized to detect color and skylight polarization in Drosophila
Source: eLife. 2021 Dec 16;10:e71858. doi: 10.7554/eLife.71858 (PMC8789284; doi:10.7554/eLife.71858)
Supplement: Supplementary file 4. [file elife-71858-supp4.pdf]

Figure(s)	Cross or Genotype	Additional information
Figure1-Di	pJFRC19-13XLexAop2-IVS-myr::GFP in su(Hw)attP8, pJFRC21-10XUAS-IVS-mCD8::RFP in attP18/ w1118; R21B12-p65ADZp in attP40/ Rh6-nlsLexAGADfl in attP40; R20E07-ZpGDBD in attP2/+	Split-GAL4 is SS01015<br>anti-dsRed, anti-GFP<br>SlowFade mounting<br>40x objective
Figure1-Dii	pJFRC19-13XLexAop2-IVS-myr::GFP in su(Hw)attP8, pJFRC21-10XUAS-IVS-mCD8::RFP in attP18/ w1118; R21B12-p65ADZpattP40/ Rh3-nlsLexAGADfl in attP40 in attP40; R20E07-ZpGDBD_attP2/+	Split-GAL4 is SS01015<br>anti-dsRed, anti-GFP<br>SlowFade mounting<br>40x objective
Figure2-Diii	S003159 (VT043162-p65ADZp in attP40; R20E07-ZpGDBD in attP2) crossed to MCFO-1<br>MCFO stock names are as in Nern et al (2015) Table S2.)	63x objective<br>DPX mounting<br>anti-Brp (mAb Nc82) reference
Figure3-Aii,Aiv	R41C05 crossed to MCFO-7	63x objective<br>anti-Brp (mAb Nc82) reference<br>DPX mounting
Figure3-Bii,Biv	VT014206 crossed to MCFO-7	63x objective<br>anti-Brp (mAb Nc82) reference<br>DPX mounting
Figure3-Ciii	SS02425 (R26H07-p65ADZp in attP40; VT010253-ZpGDBD in attP2) crossed to MCFO-1	63x objective<br>anti-Brp (mAb Nc82) reference<br>DPX mounting
Figure4-Dv	SS02978(R42E06-p65ADZp in attP40; VT064564-ZpGdbd-ZpGDBD in attP2) crossed to MCFO-1	63x objective<br>anti-Brp (mAb Nc82) reference<br>DPX mounting
Figure4-Dvi	SS02978 crossed to pJFRC51-3XUAS-IVS-Syt::smHA in su(Hw)attP1, pJFRC225-5XUAS-IVS-myr::smFLAG in VK00005	63x objective<br>anti-Brp (mAb Nc82) reference<br>DPX mounting
Figure5-Ci,Cii	SS28175 (R51E06-p65ADZp in attP40; R15D05-ZpGDBD in attP2) crossed to pJFRC51-3XUAS-IVS-Syt::smHA in su(Hw)attP1, pJFRC225-5XUAS-IVS-myr::smFLAG in VK00005	overlay of a registered image with the template used for registration<br>63x objective<br>DPX mounting

Figure5-Ciii	SS28175 crossed to MCFO-1	overlay of a registered image with the template used for registration<br>63x objective<br>DPX mounting
Figure5-Civ	SS28175 crossed to MCFO-1	overlay of a registered image with the template used for registration (the MCFO labeled cells are from the same optic lobe)<br>63x objective<br>DPX mounting
Figure5-Cv	ML-VPN1: SS28175 crossed to MCFO-1<br>and L2 neuron terminals in the medulla:<br>SS00801 (R53G02-p65ADZp in attP40; R29G11-ZpGDBD in attP2) crossed to pJFRC51-3XUAS-IVS-Syt::smHA in su(Hw)attP1, pJFRC225-5XUAS-IVS-myr::smFLAG in VK00005 (only synaptotagmin-HA pattern shown)	overlay of registered images showing a single ML-VPN1 cell and L2 terminals in the medulla<br><br>63x objectives<br>DPX mounting
Figure5-Cvi	pJFRC19-13XLexAop2-IVS-myr::GFP in su(Hw)attP8, pJFRC21-10XUAS-IVS-mCD8::RFP in attP18/ w1118; R51E06-p65ADZpattP40/ Rh6-nlsLexAGADfl in attP40; R15D05-ZpGDBD_attP2/+	Split-GAL4 is S28175<br>anti-dsRed, anti-chaoptin (mAb 24B10), native GFP for Rh6 marker<br>40x objective<br>SlowFade mounting
Figure5-figure supplement 1	pJFRC19-13XLexAop2-IVS-myr::GFP in su(Hw)attP8, pJFRC21-10XUAS-IVS-mCD8::RFP in attP18/ w1118; R51E06-p65ADZpattP40/ Rh5-nlsLexAGADfl in attP40; R15D05-ZpGDBD_attP2/+	Split-GAL4 is S28175<br>anti-dsRed, anti-chaoptin (mAb 24B10), native GFP for Rh5 marker<br>40x objective<br>SlowFade mounting
Figure7-Aiv	ortC2b<br>-Gal4;ortC2b<br>-Gal4 crossed to MCFO-1	63x objective<br>VECTASHIELD mounting
Figure7-Civ	ortC2b<br>-Gal4;ortC2b<br>-Gal4 crossed to MCFO-1	63x objective<br>VECTASHIELD mounting
Figure8-Aii	R56F07 crossed to MCFO-1	anti-cadherin (mAb DN-Ex #8) reference<br>63x objective<br>VECTASHIELD mounting
Figure8-Avi	R56F07 crossed to MCFO-1	Photoreceptor neurons labeled with anti-chaoptin (mAb 24B10)<br>anti-cadherin (mAb DN-Ex #8)

		63x objective<br>VECTASHIELD mounting
Figure8-Bii	OL0007B ((R35D04-p65ADZp in attP40; R65B05-ZpGDBD in attP2) crossed to MCFO-1<br><br>The image stack used is from Wu et al 2016.	20x objective<br>anti-Brp (mAb Nc82) reference<br>DPX mounting
Figure8-Bvi	OL0007B crossed to 20XUAS-CsChrimson-mVenus in attP18	Photoreceptor neurons labeled with anti-chaoptin (mAb 24B10)<br>40x objective<br>SlowFade mounting
Figure8-figure supplement 1	20XUAS-CsChrimson-mVenus in attP18/+; R24F06-p65ADZp in attP40/+; VT000770<br>-ZpGDBD in attP2/+	Single image of this AD/DBD combination from split-GAL4 screening<br>anti-Brp (mAb Nc82) reference<br>20x objective<br>DPX mounting
Figure9 -Av	R70E04 crossed to MCFO-7	anti-Brp (mAb Nc82) reference<br>20x objective<br>DPX mounting
Figure9 -Av inset	VT047171 crossed to MCFO-7	anti-Brp (mAb Nc82) reference<br>63x objective<br>DPX mounting
Figure9-Bv	R71A09 crossed to MCFO-7	anti-Brp (mAb Nc82) reference<br>20x objective<br>DPX mounting
Figure9-Cv	R72C08 crossed to MCFO-7	anti-Brp (mAb Nc82) reference<br>20x objective<br>DPX mounting
